# Synthesis and characterisation of novel composite sunscreens containing both avobenzone and octocrylene motifs†

**DOI:** 10.1039/d3ra02252h

**Published:** 2023-06-07

**Authors:** Adam M. Cowden, Abigail L. Whittock, Emily L. Holt, Vasilios G. Stavros, Martin Wills

**Affiliations:** a Department of Chemistry, University of Warwick Coventry CV4 7AL UK m.wills@warwick.ac.uk; b Molecular Analytical Science Centre for Doctoral Training, Senate House, University of Warwick Coventry CV4 7AL UK; c Analytical Science Centre for Doctoral Training, Senate House, University of Warwick Coventry CV4 7AL UK

## Abstract

Avobenzone and octocrylene are popular sunscreen active ingredients. Experiments that probe the stability of avobenzone in binary mixtures with octocrylene are presented, together with the synthesis of a class of novel composite sunscreens that were designed by covalently linking avobenzone and octocrylene groups. Spectroscopy, both steady-state and time-resolved, of the fused molecules was performed to investigate the stability of the new molecules and their potential function as ultraviolet filters. Computational results are detailed for truncated versions of a subset of the molecules to reveal the energy states underlying the absorption processes of this new class of sunscreen. The results indicate that the combination of elements of the two sunscreen molecules into one molecule creates a derivative with good stability to UV light in ethanol and in which the main degradation pathway of the avobenzone component in acetonitrile is reduced. Derivatives containing *p*-chloro substituents are particularly stable to UV light.

## Abstract 2

Compounds 10a–10e and 14a/b contain elements of the two established sunscreen molecules avobenzone and octocrylene. The compounds were prepared, characterised and were found to have broad-wavelength, UV-absorbing properties.

## Graphical abstract

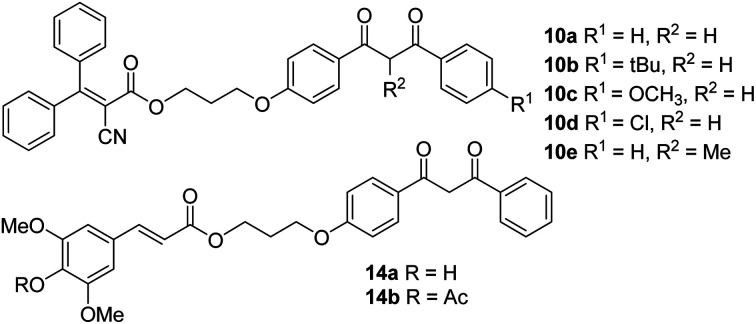

## Introduction

**Fig. 1:**
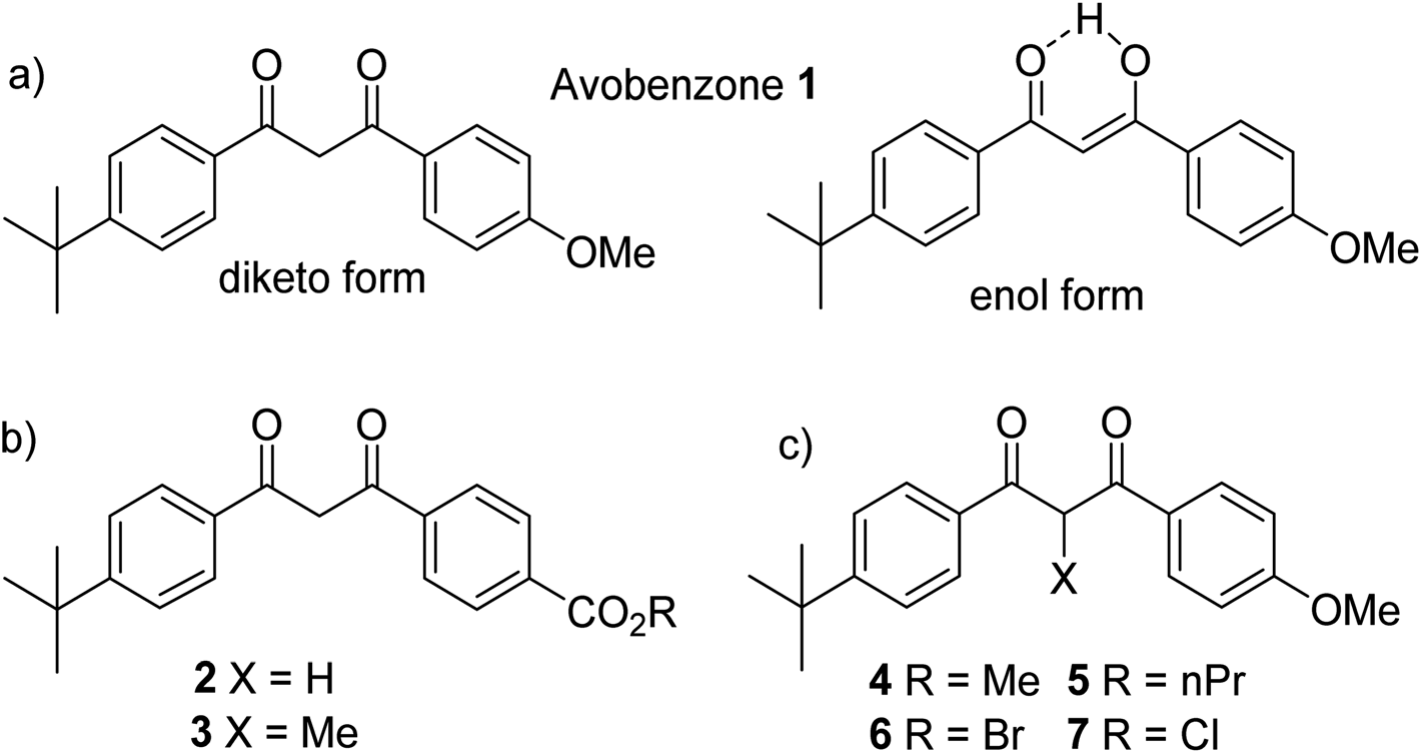
(a) Avobenzone 1, keto and chelated enol forms. (b) Ester and carboxylic acid derivatives. (c) α-Substituted dibenzoylmethanes which exist predominantly in the diketo form.

Avobenzone 1 (Fig. 1a) is a sunscreen active ingredient that is included in the Federal Drug Administration's (FDA) 1999 monograph of Sunscreen Drug Products for Over-the-Counter Human Use and in the European Chemical Agency's allowed UV filters under the Cosmetics Products Regulation.^1,2^ In International Nomenclature of Cosmetic Ingredients (INCI) terms, 1 is referred to as butyl methoxydibenzoylmethane (BM-DBM) but it is also found under a number of commercial names including Parsol® 1789, Eusolex® 9020 and Neo Heliopan® 357. Avobenzone is approved under EU REACH regulations and, in 2010, apart from TiO_2_ and oxybenzone, was the only approved UVA filter world-wide.^3^

A review of the evolution of sunscreen products in the USA notes that protection against UVA irradiation (315–400 nm) was specifically addressed by the introduction of dibenzoylmethane derivatives, with avobenzone being the most prominent member of this class.^4^ The proportion of products containing a known UVA filter such as avobenzone (using sample sizes between 60 (in 1997) and 330 (in 2009)) increased from 5% to 70% in 12 years.

Holt and co-workers extended the work of previous researchers on avobenzone photochemistry^5–16^ (for details of avobenzone photofragmentation see the ESI†) by investigating the ultrafast dynamics of avobenzone in ethanol, cyclohexane and two common emollients; di-isopropyl adipate and lauryl lactate.^17^ In all cases, after laser pulse excitation at 350 nm, the evolution of the chelated enol form out of the initially excited Franck–Condon region was described by a time constant, *τ*_1_ on the order of 100s of fs.^17^ While it is possible that there are additional ultrafast processes occurring within this time for an excited state of the molecules, such as excited-state intramolecular proton transfer, or rotation around the C–O single bond of the hydroxyl group to form non-chelated enol avobenzone species, the closeness of *τ*_1_ to the instrument response function meant that other ultrafast branching pathways could not be defined in this experiment. Decay of the initially excited S_1_ state by stimulated emission (SE) then occurred with second time constant, *τ*_2_; with a faster decay in the non-polar solvent relative to the polar solvent. It is noted that the 1–2 ps duration of this SE is probably too short to describe fluorescence (*i.e.*, natural release of a photon), as the latter typically occurs on a nanosecond or longer timescale; but is rather a photon-catalysed process (*i.e.*, SE). A positive offset at ∼550 nm that persisted beyond the window of the experiment was attributed, in part, to the formation of a triplet state of enolic avobenzone (*i.e.*, ^3^nπ* ← ^1^ππ*) which agreed with time-dependent density functional theory (TD-DFT) calculations. The relaxation of the vibrationally hot chelated enol form to the ground state was described by a third time constant, *τ*_3_ (∼8 ps); and incomplete ground state recovery was described by a fourth time constant, *τ*_4_ (>2.5 ns). The observed incomplete ground state recovery was attributed to the formation of photoisomers or photoproducts. A final significant observation was that changes in concentration (between 1 mM and 10 mM) and changes in temperature (room (18–20 °C) *vs.* skin temperature (33–37 °C)) had no significant impact on the observed photoprotection mechanism. The strong absorption in the UVA by 1 supports its use as a sunscreen but the possibility of side-reactions evidenced by incomplete ground state recovery suggests that there is opportunity for improvement.

Substituted dibenzoylmethane derivatives may be prepared by condensation of a ketone with an ester in the presence of a strong base. As well as avobenzone, isopropyl-dibenzoylmethane and butylmethoxydibenzoylmethane are typical commercial dibenzoylmethane derivatives.^18^ In a synthetic study by Moi *et al.*, the –OMe group of avobenzone was replaced with an acid (in 2) and an ester (in 3) (Fig. 1b) *via* condensation of 4-*t*-butyl acetophenone and 4-formyl benzoic acid to form a chalcone.^19^ The chalcone was submitted to bromination and then hydrolysis to yield 2, and esterification of yielded 3. These modifications were chosen with the purpose of retaining UV activity as they are distant (and therefore presumably do not disrupt) the essential 1,3-dicarbonyl unit that absorbs UVA in the enol form.

The experimental UV spectra of 2 and 3 are broadly similar to avobenzone, allowing for a shift in the enol maximum absorbance peak slightly to 352 nm, from the maximum at 356 nm for avobenzone (measurements in ethanol).^19^ The avobenzone ketone form absorbance is known to be at 265 nm,^8^ whereas both 2 and 3 absorb at 255 nm.^19^ The authors also calculated values which closely matched those observed. An increase in the keto forms during extended irradiation of both 2 and 3 was observed in acetonitrile, which is consistent with the known behaviour of avobenzone.^5–17^ Photodegradation in all molecules, using the concentration of the enol form as the initial concentration with natural sunlight as source, was observed and attributed to Norrish type 1 cleavage (CO–C) products, *i.e.* producing carbon-based radicals after ketonization.^19^ After a total of 6 hours' irradiation, 1 and 3 had degraded completely, but 2 had not yet reached 50% degradation; indicating that the latter has a half-life longer than 6 hours. Further analysis indicated that in all solvents tested (ethanol, acetonitrile, dimethyl sulfoxide and ethyl acetate) acid 2 was more stable than avobenzone 1, and apart from in ethanol, avobenzone-ester 3 was also more stable than avobenzone 1. Computational evidence suggested that the bonds next to the carbonyls are strengthened in the excited state by both substitutions, and therefore experience increased stability to α-cleavage. This conclusion opens the possibility of investigating a range of modifications that could explore the electronic and steric effects of different substituents. Other studies have investigated how blocking the keto–enol tautomerisation can facilitate the study of the diketo form.^20^ Wang and co-workers prepared molecules 4–7, with a range of substituents on the C2-position (Fig. 1c). In all cases, keto–enol tautomerisation was blocked and the diketo form predominated, presumably due to the effect of steric hindrance. Extended studies of avobenzone degradation have been reported and are discussed in the ESI,†^21–25^ as is the mechanism of photostabilisation by avobenzone and derivatives.^26–34^

**Fig. 2:**
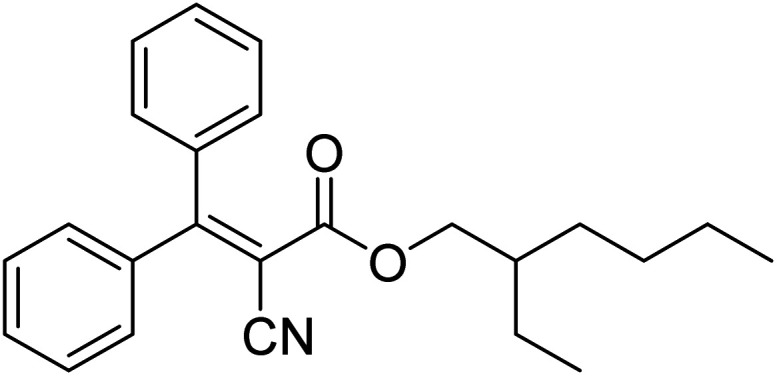
Octocrylene 8.

Another commonly used UV filter, octocrylene 8 (Fig. 2), a UVB (280–315 nm)/short-UVA absorber, is formed by the condensation of diphenylcyanoacrylic acid with 2-ethylhexanol.^35^ It is currently permitted in up to 10% by weight in a formulation under Cosmetics Products Regulation. Octocrylene has excellent photostability and thus it is included in sunscreens to increase the overall stability of the formulation.^36^ The combination of avobenzone and octocrylene has seen a rapid increase in popularity in recent years.^37,38^ A survey revealed that the avobenzone/octocrylene combination was not present in any of 48 chosen products in 1999. However this increased to 12% in 118 products in 2003; then further to 54% of 141 products in 2009.^4^

As octocrylene shows no significant fluorescence or phosphorescence, this suggests that ultrafast processes outcompete spontaneous emission.^39^ In other cinnamates related to octocrylene, the dominant relaxation pathway has been shown to be isomerisation around the C=C double bond.^40^ A synthetic study confirmed that the lipophilicity can be reduced by making a series of octocrylene analogues in two steps from cyanoacetic acid.^41^ Removing one of the phenyl rings adjacent to the double bond and functionalising the remaining ring produced a series of promising UVA and UVB filters.^41^

In quantum chemical computational analysis of interactions between avobenzone and octocrylene,^33^ singlet–singlet energy transfer between the relaxed singlet excited state of the keto form of avobenzone and ground state octocrylene is shown to be unlikely due to the large difference in energies (295 kJ mol^−1^*vs.* 351 kJ mol^−1^, respectively). In contrast, triplet-to-triplet energy transfer is more likely between the relaxed triplet state of the keto form of avobenzone and the ground-state of octocrylene (253 kJ mol^−1^*vs.* 260 kJ mol^−1^). To support the hypothesis of a quenching effect between avobenzone and octocrylene, emulsions in which the molecules were combined showed a significant increase in photostability; even when the background effect of spectral overlap was considered. Further details of the photochemical properties of octocrylene^42,43^ are described in the ESI.†

Given the known UV-protective properties of avobenzone and octocrylene, we have designed and prepared a series of molecules which contain a covalently linked combination of the essential structural components of each of these molecules. The targeted molecules were prepared in a short synthetic sequence, studied by a range of spectroscopic and laser-based analytical techniques, and were found to benefit from the combination of the UV-absorbing properties of each structural component. Our results indicated that the novel compounds absorb UV light over a broad spectrum and have good stability to UV light in ethanol with improved stability in acetonitrile compared to avobenzone alone.

## Results and discussion

**Fig. 3:**
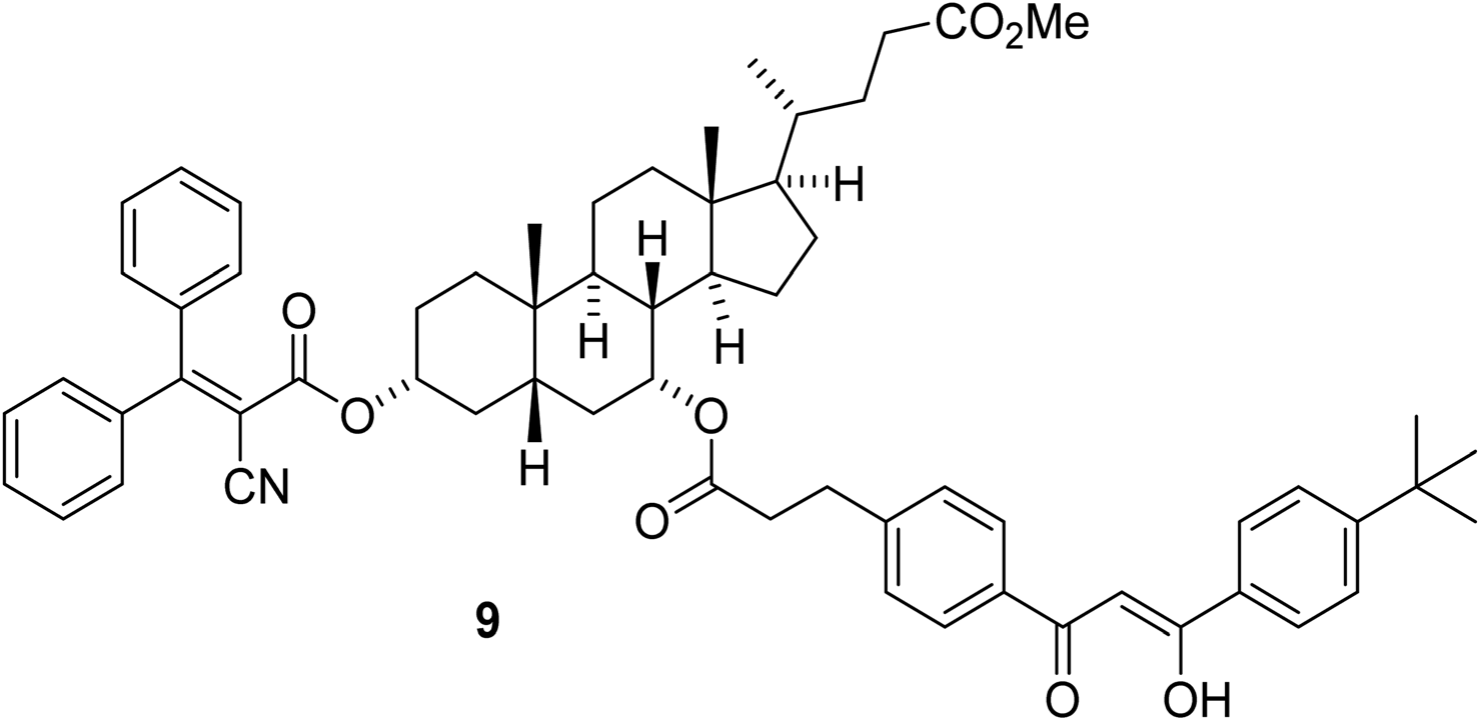
Sunscreen molecule 9 with the methyl ester of chenodeoxycholic acid linked to the UVB chromophore from octocrylene and the UVA chromophore from avobenzone.

The covalent binding of avobenzone and octocrylene *via* a cholic acid molecule, in 9 (Fig. 3) has been reported.^44^

**Fig. 4:**
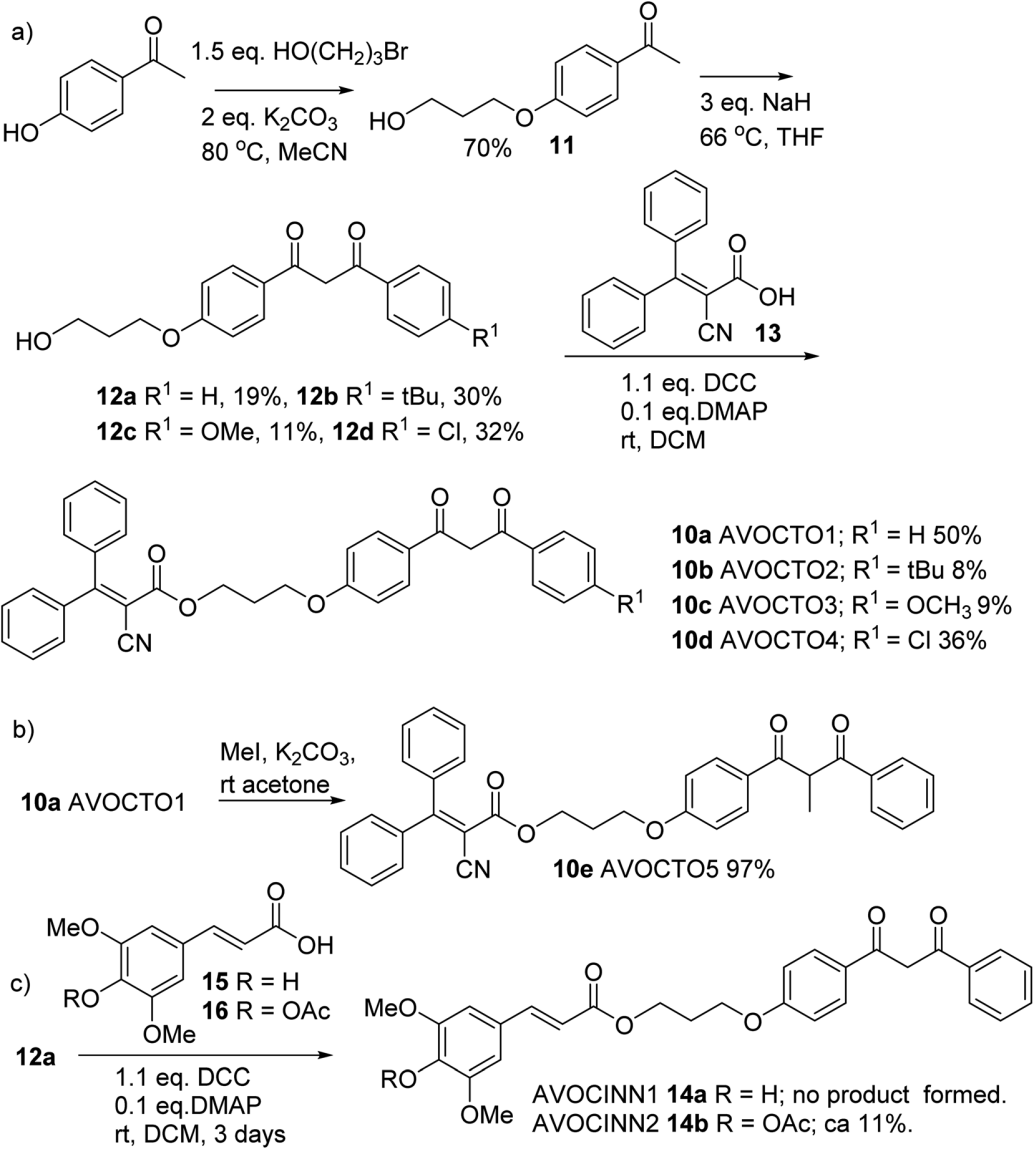
Synthesis of 10a–10e. (a) Synthetic route to 10a–10d. (b) Step from 10a to 10e. (c) Synthesis of 14b. A 2 : 1 mixture of 14a : 14b was formed through partial hydrolysis of 14b.

The photostability of 9 was measured in the presence of UV radiation (5.5 mW cm^−3^), irradiated on plates and quantified by ultraviolet absorption densitometry analysis. It showed a significant improvement in stability *versus* the avobenzone and octocrylene controls, although there was no significant change to the peak maxima of each chromophore compared to the parent molecules. The peak maxima of the composite molecule 9 are reported as 358 nm and 290 nm. This study suggested that a simpler inert linker could produce a similar improvement in stability. Hence, our series of molecules 10a–10e (‘AVOCTO1–5’) was designed with a 3-C linker to investigate the effect of linking these chromophores. The route to these is illustrated in Fig. 4.

4-Hydroxyacetophenone was reacted with 3-bromoethanol in the presence of a base to afford phenyl ether alcohol 11 (Fig. 4a),^45^ which was then reacted with a substituted ester in NaH/THF to afford 1,3-dicarbonyl compounds 12a–12d, of which 12a is known.^46^ Each of 12a–12d was then reacted with the free carboxylic acid 13 derived from octocrylene. Typical reactants for this transformation are coupling agent such as dicyclohexyl carbodiimide (DCC), a base and a catalyst such as 4-dimethylaminopyridine (DMAP) or the coupling additive 1-hydroxybenzotriazole (HOBt).^47^ We found that the Steglich conditions of DCC/DMAP provided 10a–10d (Fig. 4a).^48^ To synthesise 10e, α-methylation of AVOCTO1 10a was required, which was achieved using MeI/K_2_CO_3_ in diethyl ether in 97% yield (Fig. 4b).

Derivatives with a cinnamic acid derived part, 14a and 14b (AVOCINN1 and 2) were also prepared; sinapate ester derivatives are a class of plant-derived UV filters.^49^ Direct condensation of sinapinic acid 15 (Fig. 4c) did not produce the desired product 14a, likely due to the free phenol group in the *para*-position to the double bond. An alternative route required acylation of 15 to give 16, condensation with 12a and deprotection to yield 14a, was proposed. This was inspired by a total synthesis by Allais *et al.* where an analogous method was used to make 2-*O*-sinapoyl-l-malate.^50^ Acetylation of 15 was achieved using acetic anhydride in pyridine, to form 16. The condensation of 12a and 16 was achieved under similar conditions to the esterification step described for the 10a–10d. Deacetylation of 14b in acid solution was only partially successful, yielding a mixture of 14a/14b in a *ca.* 2 : 1 ratio (Fig. 4c).

**Fig. 5:**
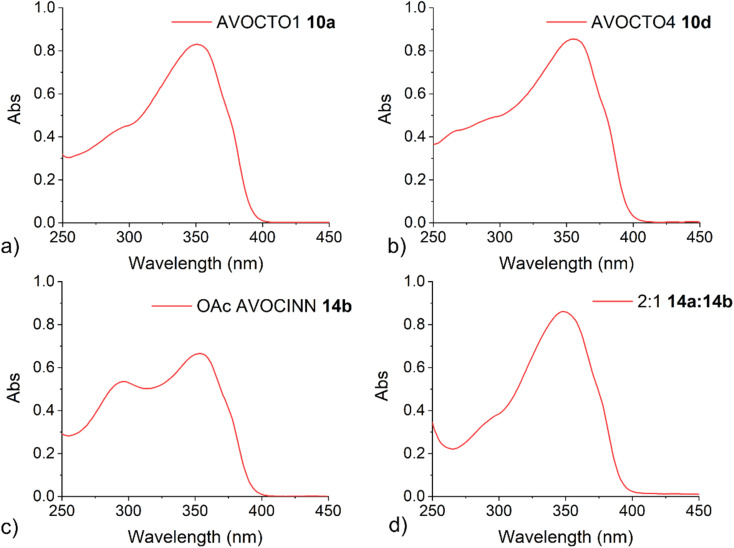
UV-vis spectra of; (a) 10a and (b) 10d. (c) 14b. (d) The partially deacylated product mixture of 14a : 14b in an approximate 2 : 1 ratio. All spectra were recorded in ethanol. Absorbance is given in arbitrary units.

The UV/vis spectra of 10a, AVOCTO 10d and AVOCINN2 14b, were recorded in ethanol (Fig. 5). AVOCINN2 14b had two distinct peaks, presumably due to the presence of two chromophores. Compounds 10a and 10d demonstrated broad protection in one molecule. A notable difference is that the shorter wavelength peak (<300 nm) is closer in intensity to the longer wavelength peak (>350 nm) in 14b than is the case for 10a and 10d. This suggests that the molar absorption coefficient (*ε*) for sinapinic acid is greater than octocrylene and closer in value to that of avobenzone.^3,51^ The UV-vis spectrum of the partially *O*-acyl deprotected product mixture (14a, 14b), was recorded (Fig. 5d) and this contained a reduced peak at the lower wavelength, indicated that deacetylation reduced the UV absorbance at this wavelength.

The effect of the stability of binary mixtures of avobenzone and octocrylene was first investigated to provide a baseline for comparison, using ethanol (Table 1) and acetonitrile as solvents (Table 2). In each case, the degradation was measured as the percentage decrease at the wavelength of peak absorbance, but also as the area under the curve ratio (AUC *R*) in order to provide a broader measure. The ESI† contains detailed results. The binary mixtures showed minimal degradation in ethanol, however in acetonitrile, the degradation of avobenzone was more rapid, even in ambient light. In ethanol, there was slightly more avobenzone decomposition when octocrylene was added, however the level was low in both cases. In acetonitrile, where avobenzone was even degraded in visible light, its combination with octocrylene appeared to reduce the level of decomposition, when the AUC *R* measurement was considered, although there was little difference at the specific point of highest absorbance.

**Table 1:** Avobenzone stability in ethanol (average of three experiments). AUC *R* is the area under the curve ratio, integrated for UVA wavelengths (315–400 nm)

Molecule/mixture	Average degradation per hour at peak absorbance in ethanol	Degradation per hour using AUC *R* (UVA)
Avobenzone 1	1.5%	1.1%
Avobenzone 1/octocrylene 8 mixture	3.0%	2.6%

**Table 2:** Avobenzone stability in acetonitrile. AUC *R* is the area under the curve ratio, integrated for UVA wavelengths (315–400 nm)

Molecule/mixture	Average loss of activity per hour at peak absorbance in acetonitrile	Degradation per hour using AUC *R* (UVA)
Avobenzone 1	−73%	−71%
Avobenzone 1 in ambient light	−6.8%	−4.5%
Avobenzone 1/octocrylene 8 mixture	−72%	−58%

As discussed, the AVOCTO compounds 10a–10e were designed as potential new sunscreen molecules, containing two chromophores in a single molecule; *i.e.*, from octocrylene and avobenzone respectively. Since the chromophores are covalently linked *via* an inert alkane (–C_3_H_6_–) linker, electronic conjugation is not expected to occur between the two chromophores. This is reflected in the broad UV-vis absorption profiles of the 10a–10e which arises due to the combined effect of the octocrylene chromophore of the molecule, covering the shorter wavelength UVB range, while the avobenzone chromophore absorbs in the UVA. It was anticipated that electronic modifications to the avobenzone chromophore by *para*-substitution could be used to tune the stability of the dicarbonyl under irradiation.

The photostability of the synthesised molecules was assessed by steady-state irradiation in ethanol and acetonitrile, using the same methodology as in the preceding avobenzone photostabilisation studies. None of the molecules degraded significantly in ethanol over the time of the experiment, which is consistent with the stability of both parent molecules in ethanol (Table 1). However, there was a significant drop in UVA absorption in some molecules in acetonitrile (Table 3, graphs are given in the ESI†), reflecting the results in Table 2, and this was the subject of further studies. Compound 10b, which is similar to avobenzone, degraded the most, with almost 50% loss of activity at the peak maximum in 40 minutes. In contrast, 10c exhibited the second highest loss of activity. For 10a, containing an unsubstituted phenyl ring, the loss of activity at the peak maximum was significantly less (−34%). Finally, *para*-substitution with a chlorine atom in 10d, resulted in significantly lower loss of activity at the peak maximum; only 5% over 40 minutes. This is reflected in the increased photostability of *para*-chloroavobenzone over avobenzone 1, which was also measured. Hence the addition of a *para*-chloro group appears to be a relatively simple method to increase the photostability of avobenzones. The absorption peak maximum of the methylated derivative 10e, in contrast, is shifted due to the predominance of the diketo form and it is not directly comparable to the other molecules; nonetheless, it demonstrated excellent photostability.

**Table 3:** Summary of the UV-vis activity of AVOCTO compounds 10a–10e in acetonitrile after 40 minutes (see Fig. S12) simulated solar irradiation using the ‘ultraslow’ set-up described in the ESI

Compound	Degradation in 40 min at peak absorbance (*λ*_max_)	Degradation in 40 min by AUC *R* (UVA, 315–400 nm)	*Para*-substituent (R^1^)
AVOCTO1 10a	−34% (350 nm)	−30.7%	H
AVOCTO2 10b	−49% (356 nm)	−43.3%	*t*Bu
AVOCTO3 10c	−41% (361 nm)	−35.7%	OCH_3_
AVOCTO4 10d	−5% (354 nm)	<−1%	Cl
AVOCTO5 10e	Peak max shifted to shorter wavelength	−10.6%	H
Avobenzone	−72% (357 nm)	−72.2%	*t*Bu
Chloroavobenzone	−14% (357 nm)	−13.1%	Cl

### Computational and ultrafast spectroscopy results of AVOCTO compounds 10a, 10d and 10e

**Fig. 6:**
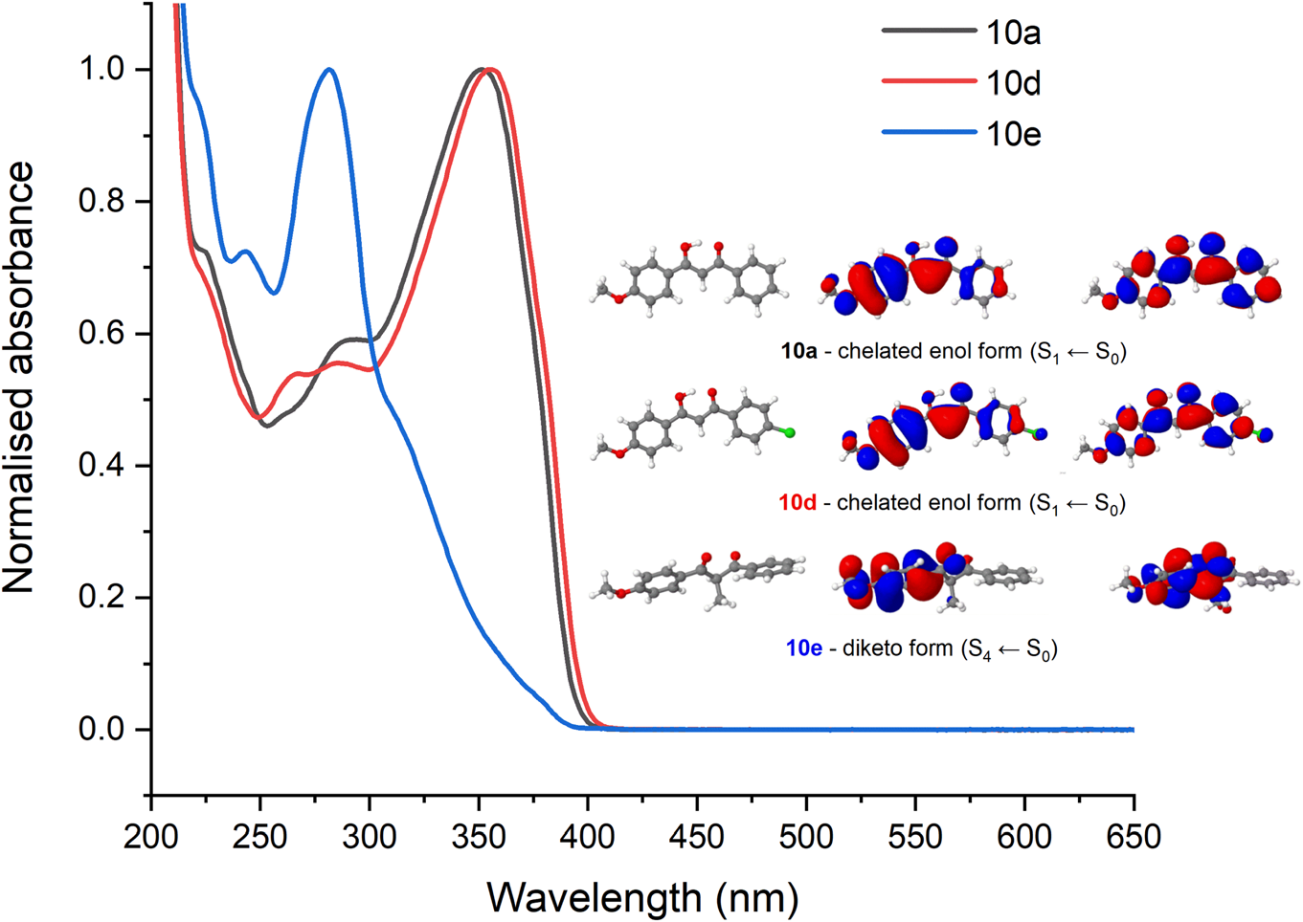
Pump wavelengths selected for the TEAS experiments on 10a and 10d are taken as the respective maxima of the absorbance spectra. The absorbance spectrum for 10e is shown for comparison but does not have significant absorbance in the 350 nm region. Inset are the computed transitions with greatest oscillator strength at the relevant optimised geometries.

To assist with the assignment of UV-visible spectra, computational studies have been conducted within this work. Geometry optimisations and vertical excitations were performed on the truncated models for 10a, 10d and 10e in both enol and diketo form as it was not possible to achieve convergence for the complete structures. Our assertion that photodynamics following UVA excitation are predominately due to the avobenzone-derived moiety allows us to truncate the molecule to only include the avobenzone-derived part in our initial geometry optimisation. Following on from the previous discussion, our calculations support the prediction that 10a and 10d are predominantly in the enol form and 10e is predominantly in the diketo form based on the vertical excitation agreement with the UV-visible absorption spectra. Specifically, the values obtained for 10a (enol, 342 nm) agrees well with experiment (350 nm) as do the values for 10d (enol, 347 nm; experimental value, 354 nm). Furthermore, our calculations have accounted for the slight redshift in the absorbance between 10a and 10d. This absorption is attributed to an allowed transition (S_1_ ← S_0_) with ππ* character. For 10e, the first bright state corresponds to the S_4_ ← S_0_ transition with ππ* character and the vertical excitation was predicted as 270 nm which is in agreement with the experimental UV-vis spectrum. The optimised geometries and the orbitals corresponding to the transition for 10a, 10d and 10e in their most dominant form are presented as an inset in Fig. 6, which also illustrates the transient electronic absorption spectroscopy (TEAS) results for each molecule. For computational details including results tables, basis set and functional, see the ESI.†

**Fig. 7:**
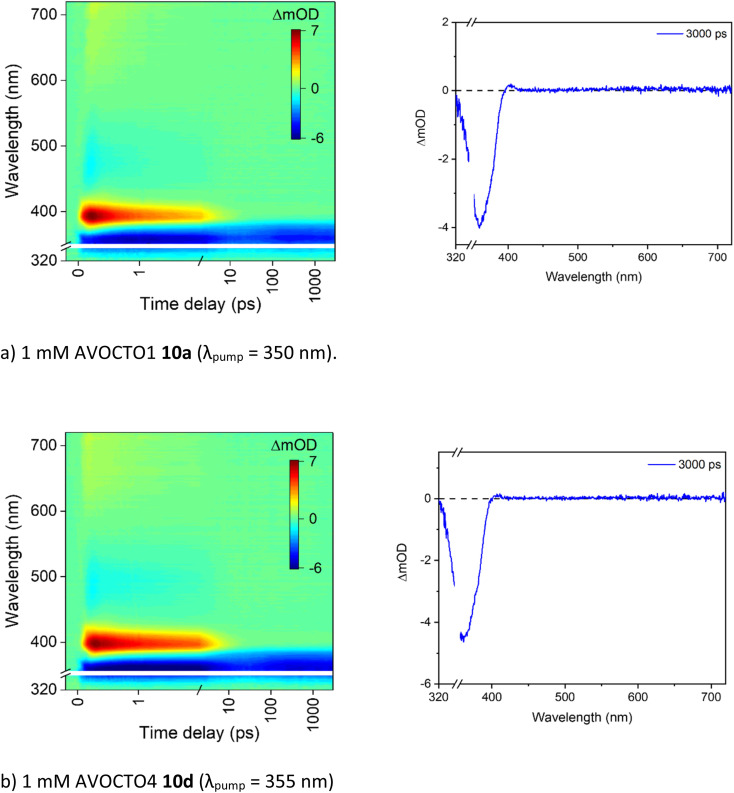
(a and b) TAS for 10a and 10d presented as a false colour heat map (left) and the 3000 ps transient (right) in acetonitrile, at the pump wavelength indicated in each case. For further experimental setup details, see the ESI.† The transient displayed is due to a ground state bleach, a feature assigned to the fourth time constant, *τ*_4_ in Table 4; and demonstrates that not all molecules have returned to the ground state within the experimental timeframe. The other processes identified in the same table, *τ*_1_ to *τ*_3_, have completed within the first 10 ps after photoexcitation.

To attempt to elucidate where the improved stability of 10d arises, the ultrafast spectroscopy analysis of molecules 10a and 10d were conducted at the Warwick Centre for Ultrafast Spectroscopy. Excitation (*i.e.*, pump) wavelengths in the UVA were chosen based on the absorption maximum of each spectrum which corresponds to the enol form of the molecule (Fig. 6). The resultant transient absorption spectra (TAS) are displayed in Fig. 7.

As both spectra are very similar, they will be described together. There is an excited state absorption (ESA) centred around 400 nm, a SE centred around 500 nm and a ground state bleach (GSB) centred around 360 nm. This is consistent with what has been observed previously for avobenzone.^17^ The TAS extracted at longer time delays presented above demonstrated that the stability of 10d in the UVA is higher than 10a. To establish whether this improved stability can be observed from the TEAS experiments, we estimated the extent of GSB recovery. No difference was observed for 10a and 10d which both showed a GSB recovery within the TEAS experimental time window of ∼20%. As a result, we conclude that the improved stability observed in the steady-state irradiations must arise from recovery of the ground state beyond 3000 ps for 10d. To further support this, an NMR study on 10d before and after TEAS measurement (Fig. 7b) revealed no degradation and is featured in the ESI.†

### Assigning the features in the TAS at pump wavelength > 350 nm

Previous ultrafast work on avobenzone conducted in our laboratory can be used to interpret the features present in the spectra above.^17^ In that report, avobenzone was studied in ethanol and cyclohexane (Table 4). A parallel global kinetic fit model was used to assign four time constants (*τ*_*n*_) to the spectral features.

**Table 4:** Extracted time constants and their assignment for avobenzone in ethanol.^17^ A plot of the best fits to the experimental data (*i.e.* evolution-associated difference spectra, EADS) is included in Fig. S15 in the ESI

Time constant	Lifetime	Description
*τ*_1_	150 ± 140 fs	The evolution of the chelated enol form of avobenzone from the Franck–Condon region^a^
*τ*_2_	1.2 ± 0.1 ps	The decay of the initially excited S_1_ state of the chelated enol form of avobenzone *via* SE^b^
*τ*_3_	8.2 ± 0.1 ps	The vibrationally hot chelated enol form of avobenzone in the ground electronic state (S_0_)
*τ*_4_	>2.5 ns	Incomplete GSB recovery due to possible formation of long-lived photoisomers

a There may be additional ultrafast processes occurring within this time on a proportion of the excited molecules, such as excited-state intramolecular proton transfer, or rotation around the C–O single bond of the hydroxyl group to form non-chelated avobenzone species.

b Given this short-lived SE, significant long-lived radiative decay due to fluorescence (which would be a barrier for sunscreen development) is not observed in the TAS spectra. See Section 7 in the ESI for further details.

The spectra obtained for AVOCTO1 10a and AVOCTO4 10d excited at 350 nm and 355 nm respectively in acetonitrile (Fig. 7) were fitted (Table 5). Avobenzone was also studied under the same conditions for comparison.

**Table 5:** Time constants obtained from fitting the TAS in acetonitrile at the longer pump wavelength. Quoted errors are from the fit in Glotaran for time constants > *τ*_2_. Experimental errors are half of the IRF in *τ*_1_ and *τ*_2_

Time constant	AVOCTO1 10a	AVOCTO4 10d	Avobenzone 1
*τ*_1_	453 ± 50 fs	513 ± 50 fs	450 ± 50 fs
*τ*_2_	3.03 ± 0.05 ps	4.05 ± 0.05 ps	2.7 ± 0.05 ps
*τ*_3_	48.3 ± 1.17 ps	73.7 ± 1.9 ps	60.9 ± 1.70 ps
*τ*_4_	>3 ns	>3 ns	>3 ns

The results for avobenzone in these experiments produce lifetimes that are longer than those of the comparison work above but the results for avobenzone are closely comparable to the results for 10a and 10d. Thus, it is believed that in AVOCTO compounds (except for 10e), excitation at ∼350 nm and the subsequent photodynamics are due to excitation of the avobenzone-derived chromophore. The lifetimes do not seem to be significantly affected by the other parts of the molecule. In keeping with the conclusion of Holt *et al.*^17^ the lifetimes are assigned to the same processes with the discrepancy due to solvent and instrumental effects.

## Conclusions

While avobenzone 1 is one of the most widely used sunscreen active ingredients, it is known to degrade over time, in particular in polar aprotic environments such as in acetonitrile. Experiments conducted using binary mixtures can indicate whether there are interactions between both molecules in solution at low concentrations but the mechanisms are often not fully understood and are beyond the scope of this paper. Composite sunscreens that are a hybrid of known efficient sunscreen molecules may provide advantages over multi-component mixtures. The new composite sunscreen molecules (AVOCTOX 10a–10e) have good stability to UV light in ethanol and address the main degradation pathway of avobenzone in acetonitrile while also providing new broadband molecules to add to the sunscreen library. The esterification reaction that forms the final ester bond can be applied to other acids and this can allow adjustment of properties that could confer further benefits and broaden the scope of the reaction scheme proposed in this paper. Further research could explore the effect of modifying the linker atoms in the AVOCTO class, with the possibility of introducing other chromophores for enhanced spectral coverage. It is also believed that the body of literature already published on avobenzone can be drawn on to understand the photodynamics of new molecules of this class given that our ultrafast spectra and computational results demonstrate that the behaviour is comparable to avobenzone itself, a result that suggests that hybrid sunscreen molecules could be imagined from other state-of-the-art sunscreens.

More studies are required to ascertain the exact mechanism of the photostability as a GSB in our ultrafast spectra suggests that the full recovery of 10d occurs at longer timescales than the ultrafast TAS experiments but at shorter times than can be observed reliably using UV-vis spectroscopy. Additionally, the larger molecular weight of a composite sunscreen such as AVOCTO or AVOCINN may also be useful in formulations as higher molecular weights are less likely to be absorbed through the skin and enter systemic circulation.^52^

## Data availability

The research data (and/or materials) supporting this publication can be accessed at https://wrap.warwick.ac.uk/.

## Conflicts of interest

The authors declare no conflicting interests.

## Supplementary Material

RA-013-D3RA02252H-s001
