# Supplementary material for: Synthesis and characterisation of novel composite sunscreens containing both avobenzone and octocrylene motifs
Source: RSC Adv. 2023 Jun 7;13(25):17017–27. doi: 10.1039/d3ra02252h (PMC10245224; doi:10.1039/d3ra02252h)
Supplement: RA-013-D3RA02252H-s001 [file RA-013-D3RA02252H-s001.pdf]

Electronic Supplementary Material (ESI) for RSC Advances.
This journal is © The Royal Society of Chemistry 2023

Supporting information

Synthesis and Characterisation of Novel Composite Sunscreens Containing both Avobenzone and Octocrylene Motifs

Adam M. Cowden,[a,b] Abigail L. Whittock,[a,c] Emily L. Holt, [a] Vasilios G. Stavros [a,d] and Martin Wills.[a*]

a. Department of Chemistry, University of Warwick, Coventry, CV4 7AL, United Kingdom.

b. Molecular Analytical Science Centre for Doctoral Training, Senate House, University of Warwick, Coventry, CV4 7AL, United Kingdom.

c. Analytical Science Centre for Doctoral Training, Senate House, University of Warwick, Coventry, CV4 7AL, United Kingdom.

d. Current address: School of Chemistry, The University of Birmingham, Edgbaston, Birmingham, B15 2TT, UK.

Contents:

1) Background information on Avobenzone and Octocrylene S2

2) Materials and Methods (Synthesis) and NMR spectra S10

3) Experimental Details: Spectroscopy S46

4) Stabilisation results of avobenzone and binary mixtures with octocrylene. S49

5) Further ultrafast laser spectroscopy results S57

6) Computational AVOCTO calculations S60

7) EADS of AVOCTO1 10a and AVOCTO4 10d S63

8) References S64

1) Background information on Avobenzone and Octocrylene.

Avobenzone: The photochemistry of dibenzoylmethane UVA filters was studied by Schwack and co-workers.[1] While UVA photodegradation was low in polar solvents such as isopropanol and methanol, avobenzone **1** was sensitive to UVA light in the non-polar solvents cyclohexane and isooctane. This significant to sunscreens as high screening efficiency cannot be guaranteed if photochemical pathways can lead to loss of UV absorbing activity. Several photoproducts were identified by HPLC and GC-MS during an 8-hour solar-simulated irradiation in cyclohexane (Figure S1).

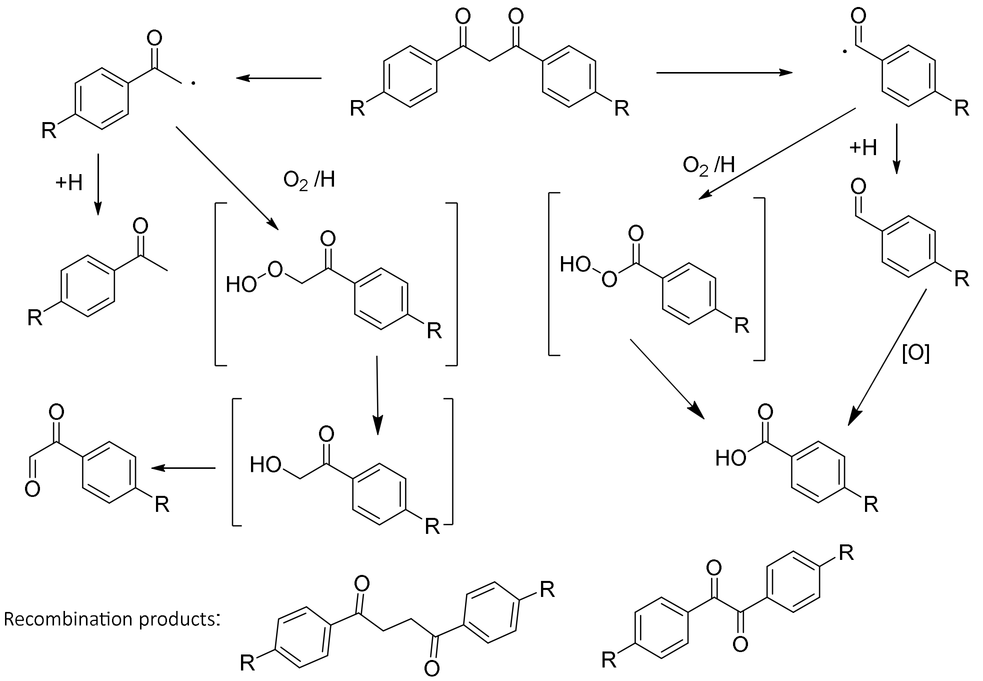

Figure S1. Photodegradation pathways of dibenzoylmethane.[1]

It was suggested that keto/enol tautomerisation in avobenzone **1** precedes fragmentation in non-polar solvents. Evidence for this can be found in complementary NMR studies that demonstrated that while the tautomerisation equilibrium lies towards the enol form, around 3.5% was detected in the diketo form in cyclohexane-d_{12} (degradation is more rapid in cyclohexane) while none was observed in deuterated polar solvents. A study by Roscher and co-workers identified only *p*-*tert*-butylbenzoic acid and *p*-methoxybenzoic acid as products from an extended irradiation, and the study suggests that the benzoic acids were

the most stable photoproducts observed.[2] The carbon-centred benzoyl and phenacyl free radicals proposed by Schwack as the key intermediates in the fragmentation pathway, were observed experimentally as electron-spin resonance (ESR) spectroscopy signals that persist for several minutes after cessation of irradiation.[3]

In an ethanolic solution of avobenzone **1**, a UV absorption band around 356 nm with ππ* character is assigned to the convolution of two degenerate *cis* enols that are stabilised by an intramolecular hydrogen bond between the 1,3-dicarbonyl moieties. Steady-state irradiation of solution phase avobenzone **1** leads to the appearance of the tautomeric keto form with an absorption of ππ* character at 265 nm, supportive of the previous inference that the diketo form is a key intermediate in degradation.[4,5] A full characterisation of the chemistry of avobenzone **1** is complicated by the presence of various tautomers and conformers. Laser (nanosecond) flash photolysis studies by Cantrell and co-workers revealed that the ground-state chelated enol form is in equilibrium with the diketo form (as discussed above) as well as two non-chelated enols that can be accessed by 355 nm laser light and rotation of the C-C bond to form the Z-isomer or the C=C bond to form the E-isomer, possibly involving an excited state intramolecular proton transfer.[6] The decay rates of the two non-chelated enols demonstrates a strong solvent dependence which is reflected in the variation in recovery time of the ground-state in different solvents. Generally, the experimental recovery rate of the chelated enol is slowest in acetonitrile, intermediate for cyclohexane and fastest in polar, protic solvents methanol and ethanol.

An excited state of the diketo form can also be accessed directly by 266 nm light.[7] A proposed wavelength-dependent mechanistic sequence based on Cantrell's findings is reproduced in Figure S2, beginning with the chelated enol form (top left).

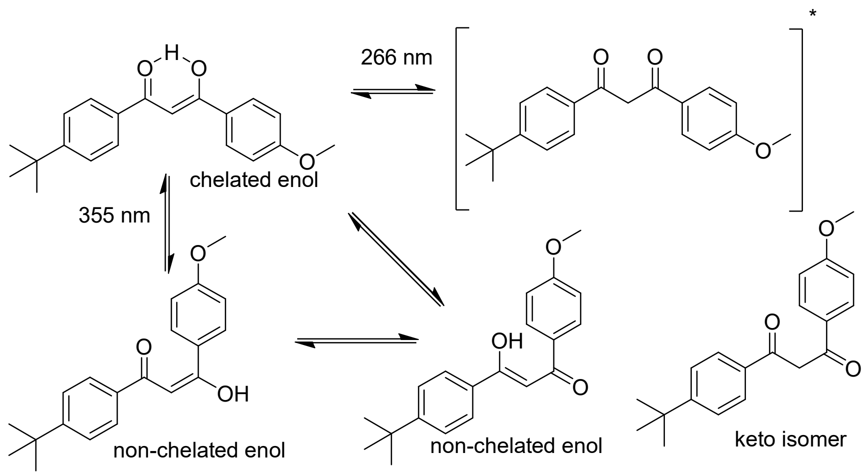

Figure S2. Mechanistic sequence based on Cantrell's findings.[6] *triplet excited state of the diketo form.

From Figure S2, it may be concluded that stabilisation of the ground-state chelated enol form to avoid formation of the excited triplet state keto will also stabilise the molecule against photodegradation. This could be by means of formulation in emollients of appropriate stabilising polarity, encapsulation, by including antioxidant additives such as glutathione or by modifying the structure of the molecule itself to shift the equilibrium towards the enol form; supported by a number of studies.[8-12] The diketo form of avobenzone that can be excited to form a triplet and/or the carbon-based radicals discussed above are potentially reactive towards biological substrates and the photosensitizing ability of avobenzone **1** has been illustrated in a number of studies where it is observed alongside increased cytotoxicity in human keratinocytes,[13] lipid peroxidation[14] and direct strand breaks in plasmid DNA.[15]

Avobenzone is thought to have synergistic effects with other sunscreen additives and could potentially react with other components in a formulation. Photo-generated fragments from avobenzone led to DNA-damaging photoproducts when irradiated in the presence of cinnamate filters such as octinoxate or EHMC, with concurrent loss of UV activity.[16] The identified mechanism followed a [2+2] cycloaddition of the enolic form of the diketone to the electrophilic alkene, followed by ring-opening (Figure S3).[17]

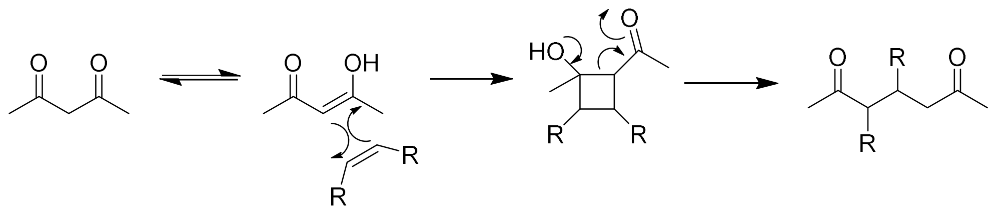

Figure S3. Reactive pathway identified for avobenzone fragmentation.

Octocrylene: Baker and co-workers identified a minor relaxation pathway that suggested the presence of a long-lived triplet state in the ultrafast transient electronic absorption spectra of octocrylene in both methanol and cyclohexane.[18] It is thought that this triplet state, which can be prepared through collision with avobenzone in the excited state, can thus improve avobenzone stability.[19] Baker et al. also do not rule out the possibility of a long-lived charge transfer state due to the -CN substituent. Their working hypothesis for a description of the excited state dynamics in the pump-probe experiment is a $\pi\pi^*$ transition caused by the pump pulse; and then an ensemble of close-in-energy $n^1\pi\pi^*$ states decay to a lower excited state with time constants τ_1 and τ_2. A third time constant, τ_3 describes further relaxation of the population in the $^1\pi\pi^*$ state. Finally, almost full recovery of octocrylene ground-state is described by τ_4 in a process mediated by a combination of intramolecular vibrational energy redistribution and vibrational energy transfer to the surrounding solvent molecules and/or isomerisation around the C=C double bond. While there are complexities around the assigning of absolute values for τ_n, an overall picture of the relaxation dynamics in octocrylene emerged: UVB photoexcited octocrylene undergoes ultrafast non-radiative relaxation which repopulates the ground state with high efficiency. The excited state dynamics are complete in the first ~5 ps after photoexcitation, with most of the processes having occurred within the first 2 ps of photoexcitation.

Photostabilisation by avobenzone.

It is thought that the majority of photostabilisers associated with avobenzone function as quenchers of avobenzone's diketo triplet state.[20] Energy can transfer efficiently between a triplet donor and acceptor when the respective energies are within 1-2 kcal (i.e. 4.18-8.37 kJ) of each other.[21] Triplet state energies can be

directly measured using oxygen perturbation, under 2000 psi oxygen, to allow for direct absorption from the ground-state to the first triplet state.[22,23] The result can be compared with phosphorescence data to obtain a good estimate of the triplet state energy (E_T). Gonzenbach and co-workers determined an E_T of 62 kcal/mol (260 kJ/mol) from phosphorescence data and an E_T of 59.5 kcal/mol (248.7 kJ/mol) with the oxygen enhanced spectrum for the enolic form of avobenzone. While these are in good comparison, the value of 248.7 kJ/mol is thought to be more precise. A literature search revealed the energy of the lowest singlet excited state (E_S) of avobenzone (enol form) to be 73.2 kcal/mol (306.2 kJ/mol)[24] and the energy of the lowest excited triplet state of avobenzone (diketo form) to be 72 kcal/mol (301 kJ/mol).[2] The energy level of the T_1 state of the keto form is much higher than that of the T_1 state of the enol form, implying that the active triplet is the diketo form (Figure S4). In a study by Kikuchi and co-workers, the excited states of avobenzone and a bis-alkylated analogues were reported. The single state energy (E_S) was obtained from the intersection point of the UV absorption and fluorescence spectra and the triplet state energy (E_T) was obtained from the first peak of phosphorescence in ethanol at 77 K.[25]

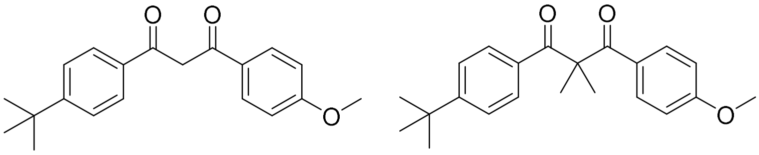

E_S(enol) 306.2 kJ/mol	-
E_S(keto) ND	E_S(keto) 322.0 kJ/mol*
E_T(enol) 248.7 or 244.0* kJ/mol	-
E_T(keto) 301.0 or 291.9* kJ/mol	E_T(keto) 293.1 kJ/mol*

Figure S4. Energies of the singlet and triplet states (of the two main forms) as summarised in the text. Energies* from Kikuchi et al.[25] were converted to kJ/mol from cm^{-1} by multiplying by hcN_a. ND – Not determined.

The key process is the interaction of the singlet and triplet states. The S_1 state of the keto form of avobenzone possesses mainly a $^1n\pi^*$ character, whereas that of the enol form possesses a $^1\pi\pi^*$ character. The intersystem crossing (ISC) between

[the singlet] $^1n\pi^*$ and [the triplet] $^3\pi\pi^*$ states should be much faster than that between $^1\pi\pi^*$ and $^3\pi\pi^*$ states, as suggested by El-Sayed's selection rule.[26]

A further prerequisite for a transfer process to occur is a limit of intermolecular distance between a donor and acceptor molecule. For triplet-triplet interaction this distance is about 1 nm, compared with the longer distance of ~10 nm for singlet-singlet processes.[27] To assess whether the mean free path (*p*) of the molecules in a given solution is on the order where such processes can occur, equation (1):

$$p = \frac{1}{\pi \cdot n \cdot d^2} \qquad (1)$$

can be used, where *n* is the number of molecules per volume (mols/L) and *d* is the molecular diameter (nm).[28] If an approximate diameter of a typical sunscreen molecule is 1 nm then a lower limit on the concentration for a triplet-triplet process could be approximated by equation (2):

$$n = \frac{1}{\pi} = 0.3183 \text{ mols/L} \qquad (2)$$

It is noted here that this concentration is much higher than the concentration used in our steady-state irradiation studies. In contrast, the concentrations used in real formulations exceed this lower limit by many orders of magnitude. Using the maximum concentration threshold from the 'Cosmetic Products Regulation, Annex VI - Allowed UV Filters' for avobenzone (=5%, molecular weight = 310.39 g/mol) and octocrylene (=10%, molecular weight = 361.48 g/mol), and an estimate for the average body surface area of an adult (ca. 1.79 m^2)[29], and applying the NHS recommended two tablespoons (ca. 30 g) gives approximate concentrations of avobenzone and octocrylene, respectively, as defined in equations (3) and (4) below:

$$n = 5\% \times \frac{30\ g}{179\ L\ x\ 310.39\ g/mol} \times \frac{6.022\ x\ 10^{23}}{mol} = 1.63 \times 10^{19}\ mols/L \qquad (3)$$

$$n = 10\ \% \times \frac{30\ g}{179\ L\ x\ 361.48\ g/mol} \times \frac{6.022\ x\ 10^{23}}{mol} = 2.79 \times 10^{19}\ mols/L \quad (4)$$

As a starting point for any discussion for the difference in stabilities between the variously substituted AVOCTO compounds in the following sections, reference can be made to the effect of para-substitution with electron donating groups (EDGs) on aromatic carbonyls (Figure S5) as studied by phosphorescence, optically detected magnetic resonance, and other optical techniques by Kikuchi et al.[25]

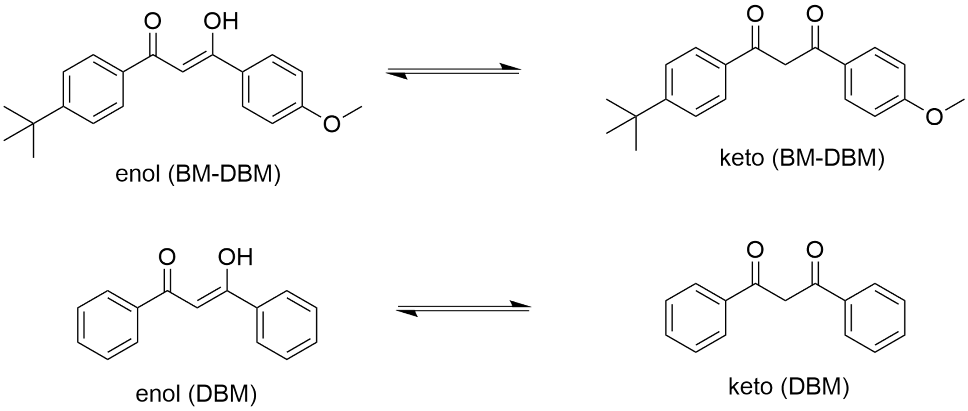

Figure S5. Molecules studied by Kikuchi et al.[25]

There is near degeneracy between the T_1 and T_2 states in many studied aromatic carbonyls which may be explained by the large spin-orbit coupling constant of the carbonyl oxygen and its important role in the mixing between the T_1 $^3\pi\pi^*$and T_2 $^3n\pi^*$ states and singlet states. The S_1 state of the keto form of BM-DBM possesses mainly $^1n\pi^*$ character, whereas that of the enol form possesses mainly $^1\pi\pi^*$ character. The intersystem crossing (ISC) between [keto] $^1n\pi^*$ and $^3\pi\pi^*$ states should be much faster than that between [enol] $^1\pi\pi^*$ and $^3\pi\pi^*$ states, as suggested by El-Sayed's rule. The energy of the T_1 state (mostly $^3\pi\pi^*$) is reduced by the effect of para-substitution with an electron donating group while the energy of the T_2 state (mostly $^3n\pi^*$) is increased; as observed experimentally when benzaldehyde is compared to p-methylbenzaldehyde and p-methoxybenzaldehyde.[30] Kikuchi and co-workers[25] observed an increase in the T_1 lifetime with avobenzone-like molecules substituted with *tert*-butyl and methoxy groups (i.e. BM-DBM vs. DBM)

and offered an explanation for the effect of para-substitution in lengthening the triplet state lifetimes: the purity of the T_1 state is recovered by an electron-donating substituent because the $^3\pi\pi^*$ excitation energy decreases. A longer triplet lifetime means that the excited state molecule remains in a high energetic state for longer and thus has a greater probability of reacting further, a property that is unfavourable to its use as a sunscreen. In this work, the effect of an electron-withdrawing group (EWG, i.e. -Cl) is studied along with EDGs (-OMe,-*t*Bu).

2) Materials and Methods (Synthesis) and NMR spectra.

Open-access LRMS and HRMS

Lower resolution mass spectra were recorded on a time of flight (TOF) mass spectrometer (Agilent 6130B single Quad) by the electrospray ionisation (ESI) method with a potential mass range of 50 - 3,000 m/z. This is coupled with a coupled with an isocratic Agilent 1100 HPLC (without column) as an automatic sample delivery system. Higher resolution mass spectra were submitted for analysis via the Departmental MS Service and recorded on a Bruker Compact Q-TOF mass spectrometer.

Open-access NMR Spectrometry

^{1}H nuclear magnetic resonance (NMR) spectra were recorded using $CDCl_3$ (δ_H = 7.26 ppm) or CD_3OD (δ_H = 4.87 ppm) as the solvent at ambient temperature on a 300 or 400 MHz spectrometer (Bruker Avance III HD) in the NMR facility at Warwick University. Data are presented as follows: chemical shift (in ppm), integration, multiplicity (s = singlet, d = doublet, t = triplet and m = multiplet), coupling constant (J/Hz) and interpretation. ^{13}C NMR spectra were recorded by broadband spin decoupling for $CDCl_3$ (δ_C = 77.2 ppm) at ambient temperature on 101 MHz. Chemical shift values are reported in ppm.

General Synthetic Details.

All solvents and reagents used were used as received by the chemical supplier without further purification. Dry solvents were used without additional drying in oven-dried glassware. Room temperature (rt) refers to ambient temperature between 20-22 °C. Overnight implies at least 12 hours. Reactants are used in 1 equivalent unless otherwise stated. “In ice” or “in an ice bath” indicates an ice bath with some added brine at a temperature <0 °C. Experiments that involve heating at a specific temperature were thermostatically controlled (±2 °C) using a hot-plate and thermostat. Reactions were monitored by thin layer chromatography (TLC) with aluminium backed silica gel 60 (F254) plates using a solvent system of equal volumes ethyl acetate and hexane (i.e., EtOAc:Hexane (1:1)). Where the solvent system used for TLC was different, it is stated. TLC spots were visualised under a short-range UV lamp (254 nm) and in most cases stained with potassium

permanganate dip dye for visualisation. Flash column chromatography was conducted on a packed silica column using 60 Å silica gel (Merck) in standard laboratory glassware. Low resolution mass spectra were obtained using an Agilent 6130B single Quad (Electrospray Ionisation, EI) mass spectrometer in either positive (+) or negative mode (-). High resolution mass spectra (HRMS) were obtained using the Mass Spectrometry service at Warwick University and are presented as a calculated and experimental mass. Melting points (MP) were determined using a Stuart Scientific SMP 1 instrument. NMR spectra (^{1}H, ^{13}C) were recorded on an in-house open-access Bruker Avance III HD (300, 400 or 500 MHz for ^{1}H NMR) spectrometer. Chemical shifts are reported in δ units, ppm relative to a solvent signal (deuterated chloroform, $CDCl_3$, deuterated water, D_2O or deuterated methanol, MeOD). *J* values are given in Hz. In the annotation of ^{13}C NMR spectra, Cipso refers to a carbon that is not bonded to a hydrogen atom. FTIR spectra were obtained on a Bruker IR spectrometer in the Warwick Teaching Laboratories, Chemistry Department.

Synthesis of precursors to avobenzone/ octocrylene (AVOCTO) compounds.

Preparation of 4-(3-hydroxypropoxy)phenylethan-1-one 11.

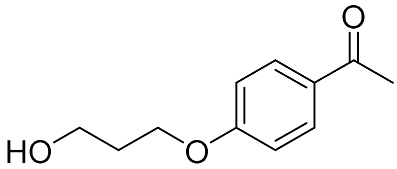

This compound was reported previously.[31] 4-Hydroxyacetophenone (544 mg, 4.0 mmol), 3-bromoethanol (0.84 g, 6.0 mmol, 1.5 eqs.) were added to acetonitrile (ca. 10 cm^3) and K_2CO_3 (1.12 g, 8.0 mmol, 2.0 eqs.) was added. The resulting mixture was stirred at 80_°C under a N_2 atmosphere overnight. At the end of this time the reaction was allowed to cool to rt and the K_2CO_3 was filtered off using gravity filtration. The filter paper was rinsed with DCM (~20-30 cm^3) to wash the product into the flask. The liquid phase was dried ($MgSO_4$), filtered and the solvent removed by high vacuum pump for 2-3 hours minimum after rota has removed the solvent to yield the product as a yellow oil (543 mg, 2.80 mmol, 70%). The product used in the next step without further purification. The data matched that reported.

TLC: R_f ~0.2; in EtOAc:Hexane (1:1), visualised by UV.

ν_{max} : 3400 (*br*, O-H stretch), 2941 and 2880 (intramolecular C=O---H-OH bond or C-H stretching), 1665 (C=O stretch) cm^{-1};

LRMS (ESI): m/z: Found [M+H]+ 195.0, $[M+Na]^+$ 217.1;

HRMS (ESI) m/z: [M + H]+ Calc'd for $C_{11}H_{15}O_3$ 195.1016; Found 195.1018 (error 1.0 ppm);

δ_H (400 MHz, $CDCl_3$): 7.94 (2H, d, J = 9.0 Hz, ArH), 6.96 (2H, d, J = 9.0, ArH), 4.21 (2H, t, J = 6.0 Hz, OCH_2), 3.90 (2H, t, J = 6.0 Hz, OCH_2), 2.58 (3H, s, CH_3), 2.10 (2H, pent, J = 6.0 Hz, CH_2), 1.76 (1H, *br*, OH) ppm.

Preparation of 4-(3-hydroxypropoxy)phenyl-3-phenylpropane-1,3-dione 12a.

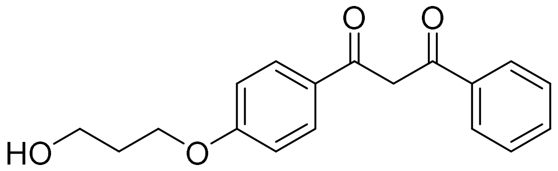

This compound was reported previously.[32] Sodium hydride (60% suspension in oil, 1.2 g, 30.0 mmol, 3 eq.) was dissolved in dry THF (10 cm^3) in a dry round bottom flask under nitrogen. The suspension was stirred for 10 mins at rt. 1-(4-(3-hydroxypropoxy)phenyl)ethan-1-one **11** (2.00 g, 10.3 mmol) was added by syringe and the mixture was stirred for 15 mins. Methyl benzoate (2.52 cm^3, 2.72 g, 20.0 mmol, 2 eq.) was added dropwise with stirring along with dry THF (10 cm^3). The solution was heated to 66 °C overnight under reflux. After cooling to rt, distilled water (ca. 5 cm^3) was added, and the solution was extracted with EtOAc (3 x 20 cm^3). The combined organic layers were washed with brine (~10 cm^3) and reduced on rotary evaporator to a crude residue. The product was isolated as a white solid (0.57 g, 1.91 mmol, 19%) using a silica column with EtOAc:Hexane (1:1) as the mobile phase. The data matched that reported.

TLC: R_f ~0.5; in EtOAc:Hexane (1:1); visualisation by UV, $KMnO_4$;

ν_{max} 3311 (O-H stretch), 3058, 2957, 2924, 2877 (C-H stretches), 1714 (C=O stretch), 1600 (enol C=C) cm^{-1};

LRMS (ESI) m/z: Found $[M+H]^+$ 299.1;

HRMS (ESI) m/z: $[M + H]^+$ Calc'd for $C_{18}H_{19}O_4$ 299.1278; Found 299.1279 (error 0.4 ppm);

δ_H (400 MHz, $CDCl_3$): 7.97 (4H, d, *J* = 7.9 Hz, ArH), 7.54-7.52 (1H, m, ArH), 7.50-7.46 (2H, m, ArH), 6.98 (2H, d, *J* = 8.6 Hz, ArH), 6.79 (1H, s, CH enol), 4.25 (2H, t, *J* = 6.0 Hz, CH_2), 3.88 (2H, t, *J* = 6.0 Hz, CH_2), 2.11-2.05 (2H, m, CH_2) ppm. OH was not observed;

δ_C (101 MHz, $CDCl_3$): 186.2 (C=O), 184.0 (C=O), 162.61 (ArC), 135.5 (ArC), 132.2 (ArC), 129.4 (ArCH), 128.7 (ArCH), 127.0 (ArCH), 114.5 (ArCH), 92.4 (CH, enol), 65.7 (CH_2), 60.0 (CH_2), 32.0 (CH_2).

Preparation of 1-(4-(*tert*-butyl)phenyl)-3-(4-(3-hydroxypropoxy)phenyl)propane-1,3-dione 12b.

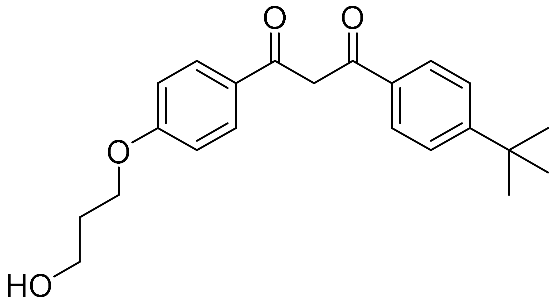

This compound is novel**.** Sodium hydride (60% dispersion in oil, 247 mg, 6.19 mmol, 3 eq.) was added to dry THF (3 cm^3) under nitrogen in a round bottom flask. A solution of (4-(3-hydroxypropoxy)phenyl)ethenone **11** (400 mg, 2.06 mmol) in dry THF (5 cm^3) was added to this suspension. The suspension was stirred for 30 mins at rt. Methyl 4-(*tert*-butyl)benzoate (793 mg, 0.80 cm3, 4.1 mmol, 2 eq.) was added slowly via syringe. The mixture thickens and so further portions of THF were added until stirring returned. The reaction was stirred at rt overnight. Distilled water (3 cm^3) was added and the product was extracted with EtOAc (3 x 10 cm^3). The combined extracts were dried under vacuum to a crude that was observed by NMR to be mostly the product contaminated with mineral oil. The product was purified by flash chromatography on silica column with a 1:1 EtOAc:Hexane mobile phase to isolate the product as an oil (220 mg, 0.62 mmol, 30%, *eluted as the second spot of two visible spots*).

TLC: R_f ~ 0.85; (visualisation by UV, $KMnO_4$);

ν_{max} 2955 and 2871 (intramolecular C=O---H-OH bond or C-H stretching), 1699 (C=O stretch), 1592 (C=C-C=O of enol form) cm^{-1};

LRMS (ESI) m/z: Found [M-] 353.2 m/z;

HRMS (ESI) m/z: $[M + H]^+$ Calc'd for $C_{22}H_{27}O_4$ 355.1904; Found 355.1901 (error 0.9 ppm);

δ_H (400 MHz, $CDCl_3$): 7.96 (2H, d, *J* = 8.6 Hz, ArH), 7.91 (2H, d, *J* = 8.1 Hz, ArH), 7.50 (2H, d, , *J* = 8.6 Hz, ArH), 6.98 (2H, d, *J* = 8.2 Hz, ArH), 6.77 (1H, s, CH enol), 4.20 (2H, t, *J* = 8.6 Hz, CH_2), 3.88 (2H, t, *J* = 8.6 Hz, CH_3), 2.10-2.04 (2H, m, CH_2), 1.36 (9H, s, *t*Bu) ppm;

δ_C (101 MHz, $CDCl_3$): 185.8 (C=O), 184.3 (C=O), 162.4 (ArC), 156.0 (ArC), 132.8 (ArC), 129.3 (2 x ArCH), 128.4 (ArC), 114.4 (ArCH), 92.1 (CH, enol), 65.6 (CH_2), 60.0 (CH_2), 31.9 (CH_2), 31.2 (3 x CH_3) ppm.

Preparation of 1-(4-(3-hydroxypropoxy)phenyl)-3-(4-methoxyphenyl)propane-1,3-dione 10c.

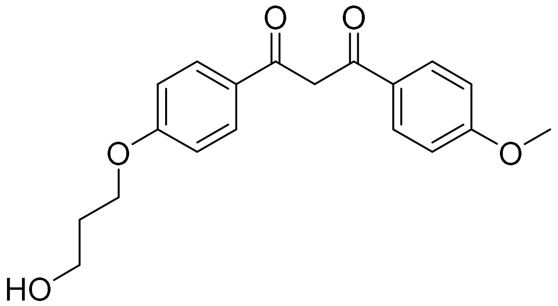

This compound is novel. Sodium hydride (60% suspension in oil, 234 mg, 5.85 mmol, 3 eq.) was dissolved in dry THF (5 cm^3) in a dry round bottom flask and placed under nitrogen. The suspension was stirred for 10 mins at rt. 1-(4-(3-Hydroxypropoxy)phenyl)ethan-1-one **11** (380 mg, 1.95 mmol, 1 eq.) was added by syringe and the mixture was stirred for 15 mins. Methyl 4-methoxybenzoate (650 mg, 3.91 mmol, 2 eq.) was added dropwise with stirring along with dry THF (5 cm^3). The solution was stirred at rt overnight. Distilled water (~5 cm^3) was added, and the solution was extracted with EtOAc (2 x 15 cm^3). The combined organic layers were washed with brine (~15 cm^3) and reduced on rota to a crude orange residue with a complex TLC (>4 spots). The product was isolated as an oil (70 mg, 0.21 mmol, 11%) using a silica column with 1:1 EtOAc:Hexane as the mobile phase.

TLC: R_f ~0.75; (visualisation by UV, $KMnO_4$);

ν_{max} 2926 and 2854 (intramolecular C=O---H-OH bond or C-H stretching), 1667 (C=O stretch);

LRMS m/z: $[M+H]^+$ Found 329.1;

HRMS (ESI) m/z: $[M + H]^+$ Calc'd for C19H20NaO5 351.1203; Found 351.1197 (error 1.8 ppm);

δ_H (400 MHz, $CDCl_3$): 7.96-7.92 (4H, m, ArH), 6.96 (4H, d, *J* = 8.8 Hz, ArH), 6.71 (1H, s, CH enol), 4.18 (2H, t, *J* = 6.0 Hz, CH_2), 3.88-3.85 (5H, m, CH_2 + OCH_3), 2.07 (2H, pent, J= 6.0 Hz, CH_2) ppm;

δ_C (101 MHz, $CDCl_3$): 184.7 (C=O), 163.1 (ArC), 162.4 (ArC), 131.4 (ArC), 129.1 (ArCH x2), 128.2 (ArC), 114.43 (ArCH), 114.0 (ArCH), 91.5 (CH, enol), 65.6 (CH_2), 60.0 (CH_2), 55.5 (CH_3), 31.9 (CH_2) ppm.

Preparation of 1-(4-Chlorophenyl)-3-(4-(3-hydroxypropoxy)phenyl)propane-1,3-dione 10d.

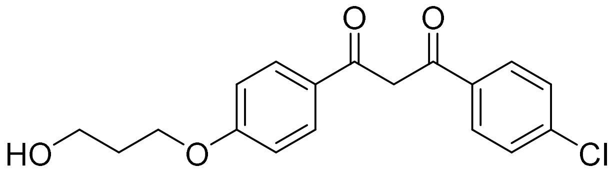

This compound is novel. Sodium hydride (60% suspension in oil, 698 mg, 17.5 mmol, 3 eq.) was suspended in dry THF (10 cm^3) in a dry round bottom flask and placed under nitrogen. The suspension was stirred for 10 mins at rt. 1-(4-(3-Hydroxypropoxy)phenyl)ethan-1-one **11** (1.13 g, 5.81 mmol, 1 eq.) was added by syringe and the mixture was stirred for 15 mins. Ethyl 4-chlorobenzoate (3.98 cm^3, 3.95 g, 21.4 mmol, 3.7 eqs.) was added dropwise with stirring along with dry THF (5 cm^3). The solution was stirred at RT overnight. Then distilled water (~5 cm^3) was added and the solution was extracted with EtOAc (2 x 20 cm^3). The combined organic layers were washed with brine (10 cm^3) and reduced under vacuum to give a crude residue. The product was isolated as a white solid (620 mg, 1.86 mmol, 32%) using a silica gel column with 1:1 EtOAc:Pet. ether as the mobile phase.

TLC: R_f ~0.8; (visualisation by UV, $KMnO_4$);

ν_{max} 3258 (O-H stretch), 2951, 2865 (C-H stretches), 1624 (C=O stretch), 1586 (enol C=C-C=O stretch) cm^{-1};

LRMS (ESI) m/z: $[M]^-$ 331;

HRMS (ESI) m/z: $[M + H]^+$ Calc'd for $C_{18}H_{18}{}^{35}ClO_4$ 333.0888; Found 333.0884 (error 1.2 ppm);

δ_H (400 MHz, $CDCl_3$): 7.98 (2H, d, *J* = 8.4 Hz, ArH), 7.90 (2H, d, *J* = 8.4 Hz, ArH), 7.45 (2H, d, *J* = 8.3 Hz, ArH), 6.98 (2H, d, *J* = 8.6 Hz, ArH), 6.74 (1H, s, enol), 4.20 (2H, t, *J* = 6.0 Hz, CH_2), 3.88 (2H, t, *J* = 6.0 Hz, CH_2), 2.11-2.05 (2H, m, CH_2) ppm;

δ_C (101 MHz, $CDCl_3$): 186.2 (C=O), 182.9 (C=O), 162.7 (ArC), 129.4 (ArC), 129.0 (ArCH x2), 128.4 (ArC), 114.4 (ArCH), 92.3 (CH, enol), 65.6 (CH_2), 60.0 (CH_2), 31.9 (CH_2) ppm.

Preparation of 2-Cyano-3,3-diphenylacrylic acid 13.

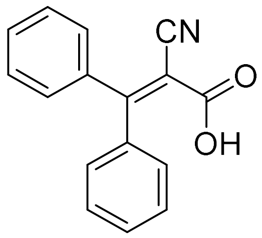

This compound has been reported and characterised.[33] Octocrylene (5.72 g, 16.4 mmol) was suspended in MeOH (20 cm^3) and LiOH (923 mg, 33 mmol, 2 eq.) was added at rt. The solution was stirred overnight at rt, after which the MeOH was removed under vacuum and water (30 cm^3) was added. The aqueous solution was extracted with EtOAc (3 x 30 cm^3), then 37% HCl was added until the aqueous solution was at pH <1. This solution was then extracted with DCM (3 x 30 cm^3), the combined extracts were dried over $MgSO_4$, filtered and the solvent removed under vacuum to give the product (2.26 g, 9.08 mmol, 55%) as a white solid. The data matched that reported.

LRMS (ESI) m/z: Found $[M]^-$ 248.25;

δ_H (500 MHz, $CDCl_3$) 7.56-7.39 (8H, m, ArH), 7.19 (2H, d, J = 7.5 Hz, ArH), acid OH was not observed;

δ_C (126 MHz, $CDCl_3$) 172.27 (CO or CN), 166.18 (CO or CN), 138.4 (ipso), 138.2 (ipso), 132.0 (ArCH), 139.9 (ArCH), 130.5 (ArCH), 129.6 (ArCH), 128.6 (ArCH), 128.2 (ArCH), 116.7 (C=C), 102.4 (C=C).

Preparation of methyl 4-methoxybenzoate.

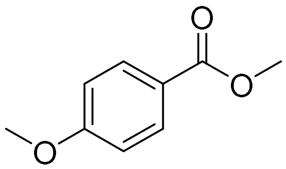

This compound has been reported.[34] 4-Methoxybenzoic acid (1.00 g, 8.20 mmol) was added to dry DMF (5 cm^3). K_2CO_3 (1.37 g, 10.0 mmol, 1.2 eq.) was added and mixture was stirred at rt for 5 mins under nitrogen. Methyl iodide (0.62 cm^3, 1.41 g, 10.0 mmol 1.2 eq.) was added dropwise by syringe and the mixture was stirred overnight at RT. Distilled water (5 cm^3) was then added and the solution was extracted with EtOAc (3 x 15 cm^3). The combined organic extracts were washed with distilled water (3 x 15 cm^3) and then with brine (15 cm^3). The organic layer was dried to yield the product (650 mg, 3.61 mmol, 48%). The data matched that reported.

TLC: R_f ~0.7; (visualisation by UV, $KMnO_4$);

δ_H (400 MHz, $CDCl_3$): 7.98-7.96 (2H, d, *J* = 8.8 Hz, ArH), 6.90-6.88 (2H, d, *J* = 8.8 Hz, ArH), 3.86 (3H, s, CH_3), 3.83 (3H, s, CH_3) ppm.

Preparation of ethyl 4-chlorobenzoate.

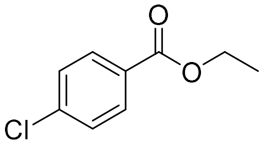

This is a known compound.[35] 4-Chlorobenzoyl chloride (0.59 mL, 0.8 g, 1 eq.) and DMAP (56 mg, 0.1 eqs.) were added in 5 ml DCM in a round bottom flask resting in an ice bath. Ethanol (0.27 mL, 0.21 g, 1 eq.) and TEA (1.27 mL, 0.92g, 2 eqs.) were added by syringe. The emulsion was then warmed slowly to RT and stirred for 1 hour. The contents of the flask were added directly to a short silica column and

eluted using DCM to yield the product as a clear liquid (500 mg, 59%) when dried under vacuum.

TLC: R_f ~ 0.75 (in 100% DCM on aluminium-backed silica gel, visualised by UV)

LRMS m/z: Calc $[M+H]^+$ C9H10ClO2+ 185.4. Found 391.3 $[2M+Na]^+$.

δ_H (400 MHz, $CDCl_3$): 7.95-7.93 (2H, d, ArH), 7.37-7.35 (2H, d, ArH), 4.37-4.31 (2H, q, CH_2), 1.37-1.34 (3H, t, CH_3)

δ_C (101 MHz, $CDCl_3$): 165.6 (C=O), 139.2 (ArC), 139.9 (ArCH), 128.9 (ArC), 128.6 (ArCH), 61.2 (CH_2), 14.3 (CH_3).

Synthesis of avobenzone/octocrylene AVOCTO compounds.

One equivalent of 2-cyano-3,3-diphenylacrylic acid **13** was dissolved in DCM (1 g/10 cm^3 solvent) under a nitrogen atmosphere in dry glassware with stirring. DCC (1.1 eq.) and DMAP (0.1 eq.) were added. The mixture was stirred at rt for 10 mins. One equivalent of the relevant alcohol was dissolved in DCM (100 mg/3 cm^3) and added in one portion. The resulting mixture was stirred overnight at rt. The mixture was filtered into a clean flask using DCM and the organic solvent is removed under vacuum. Flash chromatography was used to isolate the product from the crude residue.

Preparation of 3-(4-(3-Oxo-3-phenylpropanoyl)phenoxy)propyl 2-cyano-3,3-diphenylacrylate, AVOCTO1 10a.

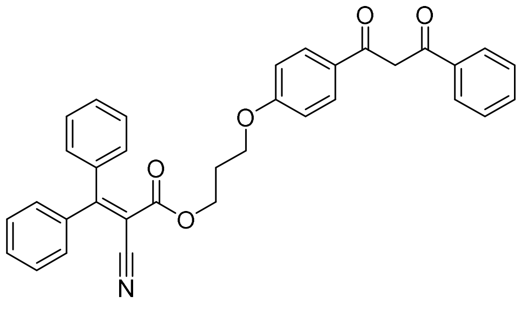

This compound is novel. 2-Cyano-3,3-diphenylacrylic acid **13** (0.33 g, 1.32 mmol) was added to DCM (5 cm^3) under nitrogen. DCC (0.30 g, 1.46 mmol, 1.1 eq.) and DMAP (16 mg, 0.13 mmol, 0.1 eq.) were added and the mixture was stirred for 10 mins at rt. 1-(4-(3-Hydroxypropoxy)phenyl)-3-phenylpropane-1,3-dione **12a** (0.40 g, 1.34 mmol) was dissolved in DCM (5 cm^3) and added to the mixture. The mixture

was stirred overnight at rt. The mixture was then passed through filter paper, and the filtrate was reduced under vacuum. A silica column with 1:1 EtOAc:Hexane as mobile phase was used to isolate the product (350 mg, 0.662 mmol, 50%) as an off-white gum. *Product elutes quickly as the 2nd spot observed on the TLC*.

TLC: R_f ~0.8. in EtOAc:Hex (1:1) on aluminium-backed silica gel; visualisation by UV, *stains brightly with $KMnO_4$*;

ν_{max} 3055 and 2928 (intramolecular C=O---H-OH bond or C-H stretching), 1715 (C=O stretch), 1592 (C=C-C=O of enol form) cm^{-1};

LRMS (ESI) m/z: Found [M+Na]+ 552.2; HRMS (ESI-TOF) m/z: [M+Na]+ Calc'd for $C_{34}H_{27}NO_5Na$ 552.1781; Found 552.1787 (error 1 ppm);

δ_H (400 MHz, $CDCl_3$): 7.98 (4H, d, *J* = 8.0 Hz, ArH), 7.54-7.16 (11H, m, ArH), 7.15 (2H, d, *J* = 7.3 Hz, ArH), 6.93 (2H, d, *J* = 8.6 Hz, ArH), 6.81 (1H, s, CH enol), 4.32 (2H, t, *J* = 6.0 Hz, CH_2), 3.94-3.91 (2H, t, *J* = 6.0 Hz, CH_2), 2.07-2.02 (2H, m ,CH_2). Singlet at 4.59 ppm is likely to be the diketo form.

δ_C (101 MHz, $CDCl_3$): 186.1 (CO or CN), 184.1 (CO or CN), 169.6 (CO or CN), 162.7 (CO or CN or Cipso or C=C), 162.3 (CO or CN or Cipso or C=C), 138.6 (Cipso or C=C), 138.2 (Cipso or C=C), 135.6 (Cipso or C=C), 132.2 (ArCH), 131.6 (ArCH), 130.6 (ArCH), 130.3 (ArCH), 129.3 (ArCH), 128.7 (ArCH), 128.6 (ArCH), 128.3 (Cipso or C=C), 127.0 (ArCH), 116.9 (Cipso or C=C), 114.5 (ArCH), 103.7 (Cipso or C=C), 92.4 (CH enol), 64.1 (CH_2), 62.6 (CH_2), 28.2 (CH_2).

Preparation of 3-(4-(3-(4-(*tert*-butyl)phenyl)-3-oxopropanoyl)phenoxy)propyl 2-cyano-3,3-diphenylacrylate, AVOCTO2 10b.

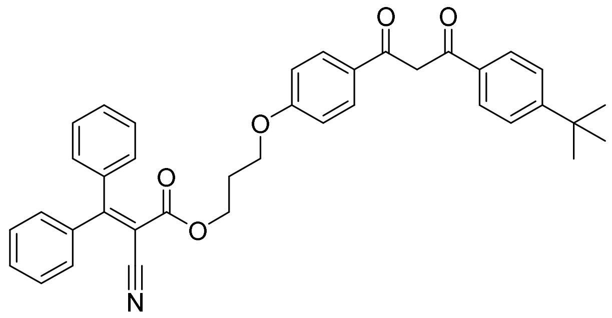

This compound is novel. 2-Cyano-3,3-diphenylacrylic acid **13** (154 mg, 0.618 mmol, 1 eq.) was added to DCM (5 cm^3) under nitrogen. DCC (141 mg, 0.684 mmol, 1.1 eq.) and DMAP (~7 mg, 0.057 mmol, 0.1 eq.) were added and the mixture was

stirred for 10 mins at rt. 1-(4-(*Tert*-butyl)phenyl)-3-(4-(3-hydroxypropoxy)phenyl)propane-1,3-dione **12b** (220 mg, 0.621 mmol, 1 eq.) was dissolved in DCM (5 cm^3) and added to the mixture. The mixture was stirred over a weekend at rt. The mixture was then passed through filter paper, and the solvent was removed under reduced pressure. A silica column with 25:75 EtOAc:Pet. ether as mobile phase was used to isolate the product in low yield (~30 mg, 0.051 mmol, 8%) as a yellow oil which on further drying formed a sticky solid residue.

TLC: R_f ~0.6; in EtOAc:Hex (1:1) on aluminium-backed silica gel; visualisation by UV, $KMnO_4$;

ν_{max} 3059, 2957 and 2865 (intramolecular C=O---H-OH bond or C-H stretching), 2215 (CN stretch), 1717 (C=O stretch), 1593 (C=C-C=O of enol form) cm^{-1};

LRMS (ESI) m/z: Found [M+Na]+ 608.26; HRMS (ESI-TOF) m/z: [M+Na]+ Calc'd for $C_{38}H_{35}NO_5Na$ 608.2407; Found 608.2406 (error 0.3 ppm);

δ_H (400 MHz, $CDCl_3$): 7.98 (2H, d, *J* = 8.1 Hz, ArH), 7.94 (2H, d, *J* = 8.1 Hz, ArH), 7.52-7.33 (10H, m, ArH), 7.15 (2H, d, *J* = 7.1 Hz, ArH), 6.94 (2H, d, *J* = 8.3 Hz, ArH), 6.81 (1H, s, enol CH), 4.32 (2H, t, *J* = 5.8 Hz, CH_2), 3.90 (2H, t, *J* = 5.8 Hz, CH_2), 2.05-2.01 (2H, m, CH_2), 1.36 (s, tBu, 9H) ppm;

δ_C (101 MHz, $CDCl_3$): 185.8 (CO or CN), 184.3 (CO or CN), 169.5 (CO or CN), 162.7 (CO or CN or Cipso or C=C), 162.3 (CO or CN or Cipso or C=C), 138.6 (Cipso or C=C), 138.3 (Cipso or C=C), 132.8 (Cipso or C=C), 131.6 (ArCH), 130.6 (ArCH), 129.4 (ArCH), 129.3 (ArCH), 128.6 (ArCH), 128.5 (ArCH), 128.3 (Cipso or C=C), 127.0 (ArCH), 125.7 (ArCH), 116.9 (Cipso or C=C), 114.5 (ArCH), 103.7 (Cipso or C=C), 92.2 (CH enol), 64.1 (CH_2), 62.7 (CH_2), 31.2 (3xCH_3), 28.2 (CH_2).

Preparation of 3-(4-(3-(4-Methoxyphenyl)-3-oxopropanoyl)phenoxy)propyl 2-cyano-3,3-diphenylacrylate, AVOCTO3 10c.

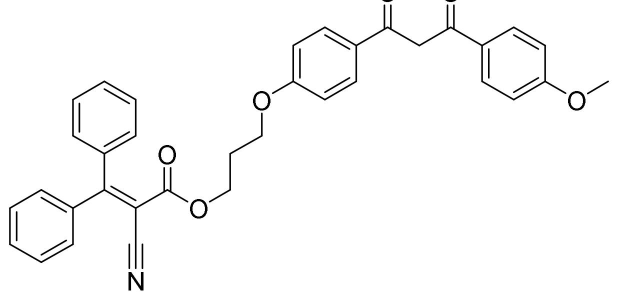

This compound is novel. 2-Cyano-3,3-diphenylacrylic acid **13** (75 mg, 0.301 mmol, 1 eq.) was added to DCM (2 cm^3) under nitrogen. DCC (68 mg, 0.33 mmol, 1.1 eq.) and DMAP (4 mg, 0.032 mmol, 0.1 eq.) were added and the mixture was stirred for 15 mins. 1-(4-Methoxyphenyl)-3-(4-(3-hydroxypropoxy)phenyl)propane-1,3-dione **12c** (100 mg, 0.305 mmol, 1 eq.) was dissolved in DCM (2 cm^3) and added to the mixture. The mixture was stirred overnight at rt under nitrogen. The mixture was then passed through filter paper, and the filtrate was reduced under vacuum to yield the crude product. A silica column with 1:1 EtOAc:Hexane as mobile phase was used to isolate the product (~15 mg, 0.027 mmol, 9%) as an oil.

TLC: R_f ~ 0.85; in EtOAc:Hex (1:1) on aluminium-backed silica gel; visualisation by UV, $KMnO_4$;

ν_{max} 3057, 2940 and 2935 (intramolecular C=O---H-OH bond or C-H stretching), 2217 (CN stretch), 1717 (C=O stretch), 1591 (C=C-C=O of enol form) cm^{-1};

LRMS (ESI) m/z: Found [M+Na]+ 582.2; HRMS (ESI-TOF) m/z: [M+Na]+ Calcd for $C_{35}H_{29}NO_6Na$ 582.1887; Found 582.1884 (error 0.6 ppm);

δ_H (400 MHz, $CDCl_3$): 7.96 (4H, 2 x d, *J* = 3.9 Hz, ArH), 7.49-7.32 (~9H, m, ArH), 7.14 (2H, d, *J* = 7.0 Hz, ArH), 6.98 (2H, d, *J* = 8.9 Hz, ArH), 6.92 (2H, d, *J* = 8.8 Hz, ArH), 6.94 (1H, s, enol), 4.32 (2H, t, *J* = 6.1 Hz, CH_2), 3.93 (2H, t, *J* = 12.1 Hz, CH_2), 3.89 (3H, s, CH_3), 2.04-2.00 (2H, m CH_2) ppm.

δ_C (101 MHz, $CDCl_3$): 186.7 (CO or CN), 184.5 (CO or CN), 163.1 (CO or CN), 162.1 (CO or CN or Cipso or C=C), 138.6 (Cipso or C=C), 131.6 (Cipso or C=C), 130.5 (Cipso or C=C), 130.3 (ArCH), 129.3 (ArCH), 129.1 (ArCH), 129.1 (ArCH), 128.6 (ArCH), 128.4 (ArCH), 128.3 (Cipso or C=C), 114.5 (Cipso), 114.0 (Cipso or C=C), 102 (C=C), 91.5 (CH enol), 64.1 (CH_2), 62.6 (CH_2), 55.5 (CH_3), 28.2 (CH_2).

Preparation of 3-(4-(3-(4-Chlorophenyl)-3-oxopropanoyl)phenoxy)propyl 2-cyano-3,3-diphenylacrylate, AVOCTO4 10d.

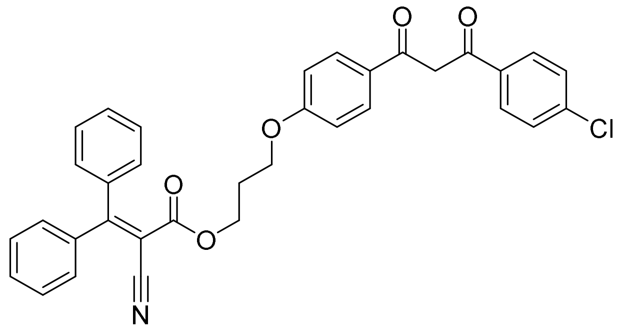

This compound is novel. 2-Cyano-3,3-diphenylacrylic acid **13** (284 mg, 1.14 mmol, 1 eq.) was added to DCM (2 cm^3) under nitrogen. DCC (258 mg, 1.25 mmol, 1.1 eq.) and DMAP (14 mg, 0.115 mmol, 0.1 eq.) were added and the mixture was stirred for 15 mins at rt. 1-(4-Chlorophenyl)-3-(4-(3-hydroxypropoxy)phenyl)propane-1,3-dione **12d** (380 mg, 1.14 mmol, 1 eq.) was dissolved in DCM (2 cm^3) and added to the mixture. The mixture was stirred overnight at rt under nitrogen. The mixture was then passed through filter paper, and the filtrate was reduced under vacuum to yield the crude product. A silica column with EtOAc:Pet. ether (25:75) as mobile phase was used to isolate the product (230 mg, 0.41 mmol, 36%) as a solid.

TLC: R_f ~0.7; in EtOAc:Hex (1:1) on aluminium-backed silica gel; visualisation by UV, $KMnO_4$;

ν_{max} 2945 and 2864 (intramolecular C=O---H-OH bond or C-H stretching), 1587 (C=C-C=O of enol form) cm^{-1};

LRMS (ESI) m/z: [M+Na]+ 586.1; HRMS (ESI-TOF) m/z: [M+Na]+ Calc'd for $C_{35}H_{22}N_5OClNa$ 586.1405; Found 586.1397 (error 1.3 ppm);

δ_H (400 MHz, $CDCl_3$): 7.97 (2H, d, J = 8.6 Hz, ArH), 7.92 (2H, d, J = 8.4 Hz, ArH), 7.50-7.32 (10H, m, ArH), 7.15 (2H, d, J = 7.4 Hz, ArH), 6.93 (2H, d, J = 8.7 Hz, ArH), 6.75 (1H, s, enol), 4.31 (2H, t, J = 6.1 Hz, CH_2), 3.94 (2H, t, J = 6.0 Hz, CH_2), 2.07-2.01 (2H, m, CH_2), 1.56 (1H, s, *possibly* H_2O);

δ_C (101 MHz, $CDCl_3$): 186.1 (CO or CN), 182.9 (CO or CN), 169.7 (CO or CN), 162.6 (CO or CN or Cipso or C=C), 162.5 (CO or CN or Cipso or C=C), 131.6 (Cipso or C=C), 130.5 (Cipso or C=C), 130.3 (Cipso or C=C), 129.4 (ArCH), 129.3 (ArCH), 129.0 (ArCH), 128.6 (ArCH), 128.4 (ArCH), 128.3 (ArCH), 128.1 (ArCH), 116.9 (Cipso or C=C), 114.6 (ArCH), 92.3 (CH enol), 64.1 (CH_2), 62.6 (CH_2), 28.2 (CH_2).

Preparation of 3-(4-(2-Methyl-3-oxo-3-phenylpropanoyl)phenoxy)propyl 2-cyano-3,3-diphenylacrylate, AVOCTO5 10e.

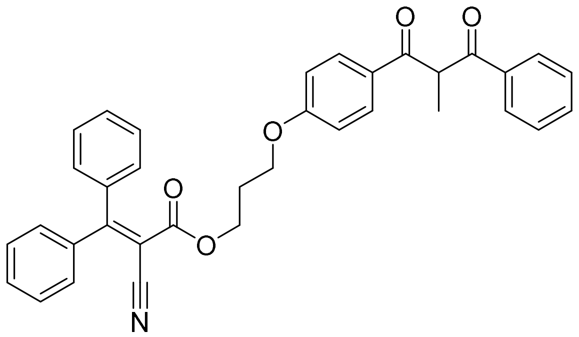

This compound is novel. 3-(4-(3-Oxo-3-phenylpropanoyl)phenoxy)propyl 2-cyano-3,3-diphenylacrylate **10** (30 mg, 0.057 mmol) was added to a suspension of K_2CO_3 (8 mg, 0.058 mmol, 1 eq.) in dry acetone (2 cm^3) and stirred for 5 mins at rt. Methyl iodide (9.6 mg, 4.2 µL, 0.068 mmol, 1.2 eqs.) was added dropwise and the mixture was stirred overnight at rt. Diethyl ether (5 cm^3) was added, and the mixture was filtered and reduced under vacuum. The product (30 mg, 0.055 mmol, 97%) was isolated as an oil by silica column using EtOAc:Hexane (1:1) as the mobile phase.

TLC: in EtOAc:Hex (1:1) on aluminium-backed silica gel: R_f of the product is slightly less than the R_f of the starting material in EtOAc:Hex (1:1), R_f ~ 0.8 (visualisation by UV/$KMnO_4$).

ν_{max} 3061, 2927 and 2856 (intramolecular C=O---H-OH bond or C-H stretching), 2216 (CN stretch), 1700 (C=O stretch), 1600, 1555 (C=C-C=O of enol form) cm^{-1};

LRMS (ESI) m/z: Found [M+Na]+ 566.2; HRMS (ESI-TOF) m/z: [M+Na]+ Calcd for $C_{35}H_{29}NO_5Na$ 566.1938; Found 566.1938 (error 0.1 ppm);

δ_H (400 MHz, $CDCl_3$) 7.95 (4H, d, *J* = 7.3 Hz, ArH), 7.56-7.30 (~10H, m, ArH), 7.13 (2H, d, *J* = 7.4 Hz, ArH), 6.88 (2H, d, *J* = 8.6 Hz, ArH), 5.21 (1H, q, *J* =7.0 Hz, C*H*CH_3), 4.30 (2H, t, *J* = 5.9 Hz, CH_2), 3.90 (2H, t, *J* = 5.9 Hz, CH_2), 2.05-1.99 (2H, m, CH_2), 1.60 (3H, d, *J* = 7.0 Hz, CH_3);

δ_C (101 MHz, $CDCl_3$): 197.3 (CO or CN), 195.8 (CO or CN), 169.6 (CO or CN), 162.9 (CO or CN or Cipso or C=C), 162.6 (CO or CN or Cipso or C=C), 138.6 (Cipso or C=C), 138.2 (Cipso or C=C), 135.8 (Cipso or C=C), 133.4 (ArCH), 131.6 (ArCH), 130.9 (ArCH), 130.5 (ArCH), 130.3 (ArCH), 129.3 (ArCH), 128.9 (ArCH), 128.6 (ArCH), 128.5 (Cipso or C=C), 128.3 (ArCH), 127.0 (ArCH), 116.8 (Cipso or C=C), 114.6 (ArCH), 103.6 (Cipso or C=C), 64.2 (CH_2), 62.5 (CH_2), 28.1 (CH_2), 14.5 (CH_3).

Preparation of 1-(4-Chlorophenyl)-3-(4-methoxyphenyl)propane-1,3-dione.

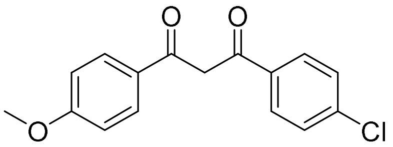

This compound has previously been characterised.[36] 4-Hydroxyacetophenone (1.00 g, 7.34 mmol) was added with K_2CO_3 (1.52 g, 11.0 mmol, 1.5 eqs.) in acetone (10 cm^3) with stirring at rt in a sealed RBF. Methyl iodide (0.68 cm^3, 1.56 g, 11.0 mmol, 1.5 eq.) was added via syringe. After 19 hours, the reaction mixture was filtered and the filtrate was concentrated under reduced pressure to yield the intermediate 1-(4-methoxyphenyl)ethan-1-one (800 mg, 5.3 mmol, 73%). A portion of the dried solid (100 mg, 0.67 mmol) was added to NaH (60% suspension in mineral oil, 72 mg, 1.8 mmol, 3 eqs.) in dry THF (4 cm^3) at rt and stirred for 10 minutes. Previously prepared ethyl 4-chlorobenzoate (222 mg, 1.2 mmol, 2 eqs.) was dissolved in dry THF (~3 cm^3) and added. The mixture was heated under a findenser to 66 °C overnight. The mixture was cooled to rt and a few mLs of distilled water were added. The resulting suspension was extracted with excess ethyl acetate (15 cm^3), washed once with brine and dried to a crude residue under reduced pressure. The product (60 mg, 0.21 mmol, 31%, off-white, slightly pink solid) was isolated on a packed silica column using 1:1 EtOAc:Hex as eluent.

TLC: R_f ~0.7; in EtOAc:Hex (1:1) on aluminium-backed silica gel; visualisation by UV, $KMnO_4$;

ν_{max} 3258 (O-H stretch), 2951, 2865 (C-H stretches), 1624 (C=O stretch), 1586 (enol C=C-C=O stretch) cm^{-1};

LRMS m/z: [M+H]+ Not detected;

δ_H (400 MHz, $CDCl_3$) 7.97 (2H, d, J = 8.6 Hz, ArH), 7.90 (2H, d, J= 8.4 Hz, ArH), 7.45 (2H, J = 8.3 Hz, ArH), 6.98 (2H, d, J = 8.6 Hz, ArH), 6.74 (1H, s, CH enol), 3.88 (3H, s, CH_3), ppm. Peak at 1.26 is attributed to H_2O;

δ_C (101 MHz, $CDCl_3$) (*literature*.[14]) 186.3 (CO), 183.0 (CO), 163.5 (C), 138.5 (C), 134.2 (C), 129.5 (CH), 129.1 (CH), 128.5 (CH), 128.1 (CH), 114.2 (CH), 92.4, 55.7 (CH_3).

Preparation of 1-(4-(Tert-butyl)phenyl)-3-(4-methoxyphenyl)-2-methylpropane-1,3-dione

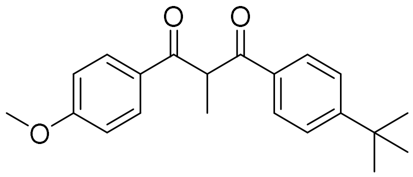

This compound has been reported and fully characterised.[37] Avobenzone **1** (200 mg, 0.64 mmol) was added with K_2CO_3 (177 mg, 1.28 mmol, 2 eq.) in acetone (10 cm^3) under nitrogen. Methyl iodide (364 mg, 165 µL, 2.56 mmol, 4 eq.) was added dropwise at room temperature. The solution was heated to 45 °C overnight. When cooled the solution was filtered through filter paper and the organic layer was concentrated under reduced pressure. A packed silica column with Hex:EtOAc (60:40) was used as eluent to isolate the product (30 mg, 0.09 mol, 14% yield) as a colourless oil.

LRMS m/z: [M+H]+ Found 325.1

δ_H (400 MHz, $CDCl_3$) 7.95 (2H, d, J = 8.7 Hz, ArH), 7.90 (2H, d, J= 8.3 Hz, ArH), 7.44 (2H, J = 8.3 Hz, ArH), 6.92 (2H, d, J = 8.7 Hz, ArH), 5.22-5.16 (1H, m, CH), 3.84 (3H, s, OCH_3), 1.57 (3H, d, J = 7.0 Hz), 1.31 (9H, s, ^tBu) ppm;

δ_C (101 MHz, $CDCl_3$) 196.9 (CO), 195.9 (CO), 163.8 (C), 157.2 (C), 133.1 (C), 130.9 (CH), 128.7 (C), 128.5 (CH), 125.8 (CH), 114.1 (CH), 55.5 (CH_3), 51.0 (CH), 35.1 (C), 31.0 (CH_3), 14.5 (CH_3).

Preparation of (E)-3-(4-acetoxy-3,5-dimethoxyphenyl)acrylic acid 16.

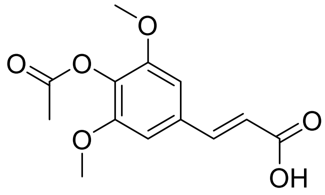

This compound has been reported and characterised.[38] Sinapinic acid (1.00 g, 4.46 mmol) was added to pyridine (1.0 mL, 0.98 g, 12.4 mmol, 2.8 eqs.) and acetic anhydride (0.99 mL, 1.07 g, 10.47 mmol, 2.3 eqs.) at rt in an RBF. The mixture was stirred at rt for 3 hours and then poured onto an ice/water mixture (10 mL) in a beaker. The resultant white precipitate was filtered and washed with H_2O (10 mL) and dried to give the product as a white solid (1.12 g, 4.21 mmol, 94%). This

product, confirmed by NMR, was used in the following steps without further purification.

δ_H (400 MHz, $CDCl_3$): 7.71 (1H, d, J= 15.9 Hz, =CH), 6.79 (2H, s, ArH), 6.40 (1H, d, J=15.9 Hz,=CH), 3.86 (6H, s, OCH_3), 2.35 (3H, s, CH_3).

Preparation of 3-(4-(3-oxo-3-phenylpropanoyl)phenoxy)propyl (E)-3-(4-acetoxy-3,5-dimethoxyphenyl)acrylate (OAc-AVOCINN) 14b.

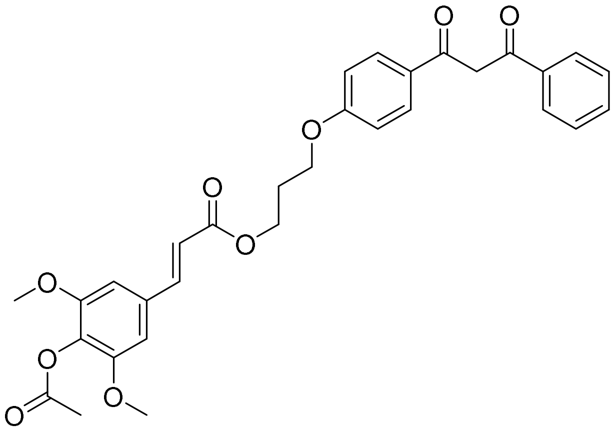

This compound is novel. 1-(4-(3-Hydroxypropoxy)phenyl)-3-phenylpropane-1,3-dione **12a** (50 mg, 0.17 mmol, 1 eq.) and (E)-3-(4-acetoxy-3,5-dimethoxyphenyl)acrylic acid **16** (45 mg, 0.17 mmol, 1 eq.) were combined in a mixture of DCC (39 mg, 0.91 mmol, 1.1 eqs.), DMAP (2 mg, 0.017 mmol, 0.1 eqs.) and DCM (5 cm^3), sealed and stirred for 3 days at rt. The solution was passed through filter paper using excess DCM and concentrated under reduced pressure. The product (<10 mg, 11%) was isolated in reasonable purity from the complex mixture as the 3rd spot on the TLC using a packed silica column with EtOAc:Hex (1:1) as eluent.

TLC: R_f ~0.6; in EtOAc:Hex (1:1) on aluminium-backed silica gel; visualisation by UV, $KMnO_4$;

LRMS m/z: [M+H]+ Found 547.1, [M+Na]+ Found 569.1;

δ_H (400 MHz, $CDCl_3$): 7.99 (4H, m, ArH), 7.62 (1H, d, J = 15.9 Hz, =CH), 7.56-7.47 (3H, m, ArH), 7.01-6.98 (2H, m, ArH), 6.80 (1H, s, =CH), 6.76-6.73 (2H, m, ArH), 6.39 (1H,

d, J= 15.9 Hz, =CH, 4.44 (2H, t, J=6.2 Hz, CH_2), 4.19 (2H, t, J = 6.2 Hz, CH_2), 3.85 (6H, s, OCH_3), 2.34 (3H, s, CH_3), 2.28-2.22 (2H, m, CH_2).

Deprotection (Deacetylation) of 3-(4-(3-oxo-3-phenylpropanoyl)phenoxy)propyl (E)-3-(4-acetoxy-3,5-dimethoxyphenyl)acrylate (AVOCINN) 14a (mixture with 14b).

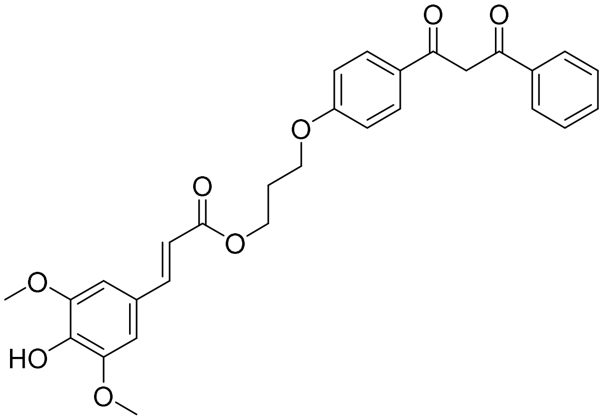

A procedure for an analogous compound was adopted.[38] A sample of 3-(4-(3-oxo-3-phenylpropanoyl)phenoxy)propyl (E)-3-(4-acetoxy-3,5-dimethoxyphenyl)acrylate **14b** (~ 20 mg) was refluxed under a findenser and positive nitrogen pressure first for 3 hours and then overnight in a 1:1 solution of 3M HCl:acetone. The progress of the reaction was monitored by the relative integrals of the peaks on the NMR spectrum corresponding to the acetyl group and the neighbouring multiplet (i.e., 2.34 (s, 3H), 2.28-2.22 (m, 2H), see above). At both time points the deprotection had produced a ratio of approximately 2 parts product **14a** to 1 part protected starting material **14b**.

NMR spectra of synthetic compounds.

^{1}H NMR (400 MHz, $CDCl_3$) of **4-(3-hydroxypropoxy)phenylethan-1-one 11.**

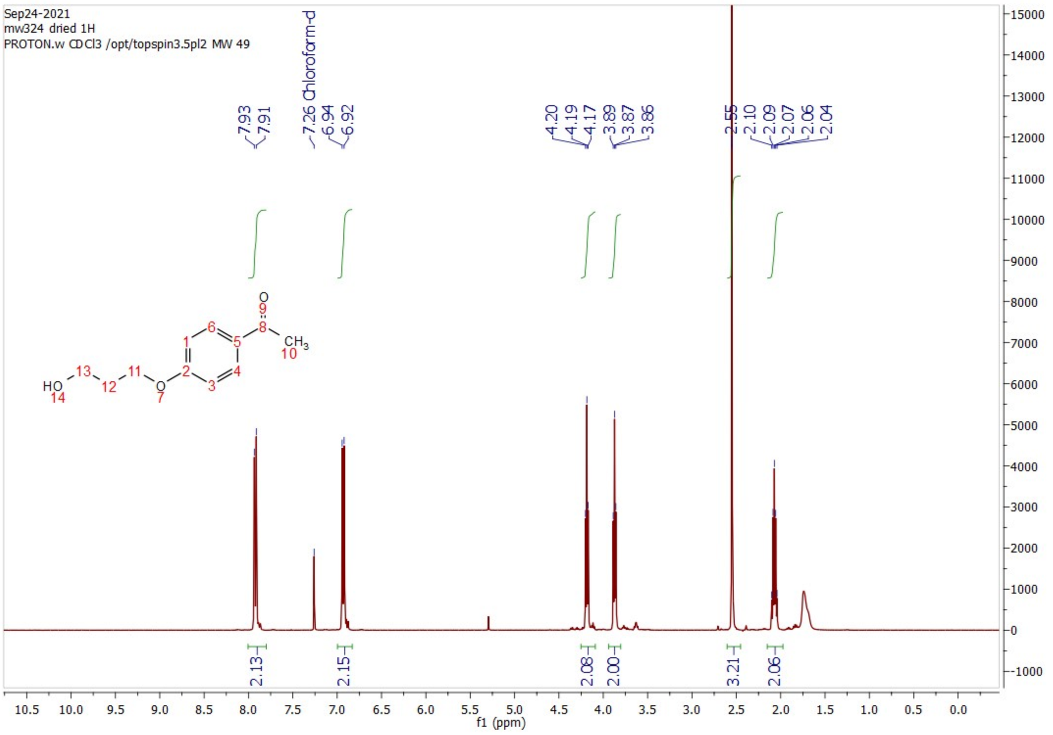

^{1}H NMR (400 MHz, $CDCl_3$) of **4-(3-hydroxypropoxy)phenyl-3-phenylpropane-1,3-dione 12a.**

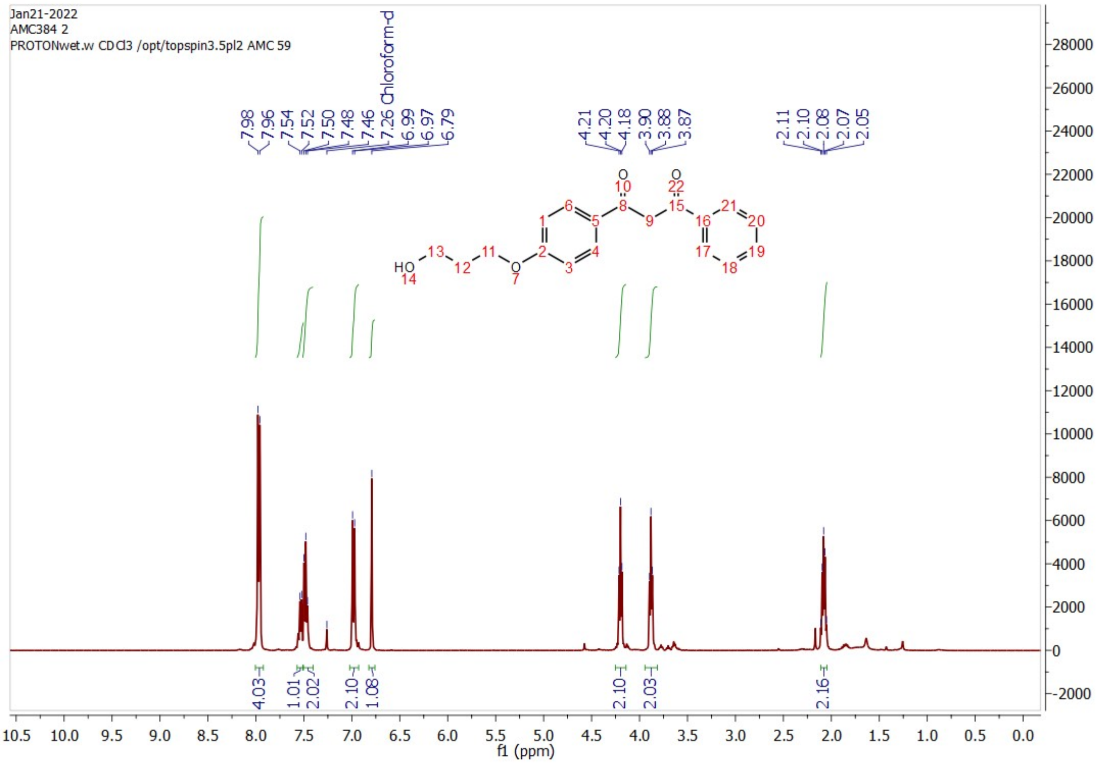

^{1}H NMR (400 MHz, $CDCl_3$) of **1-(4-(*tert*-butyl)phenyl)-3-(4-(3-hydroxypropoxy)phenyl)propane-1,3-dione 12b.**

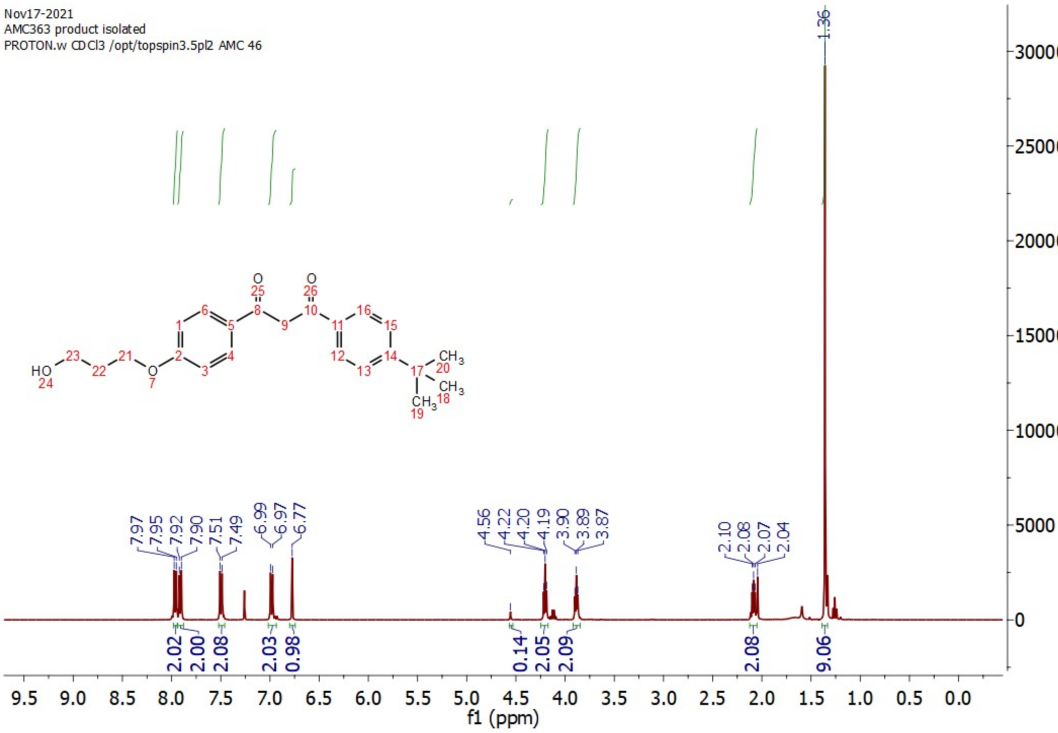

^{13}C NMR (101 MHz, $CDCl_3$) of **1-(4-(*tert*-butyl)phenyl)-3-(4-(3-hydroxypropoxy)phenyl)propane-1,3-dione 12b.**

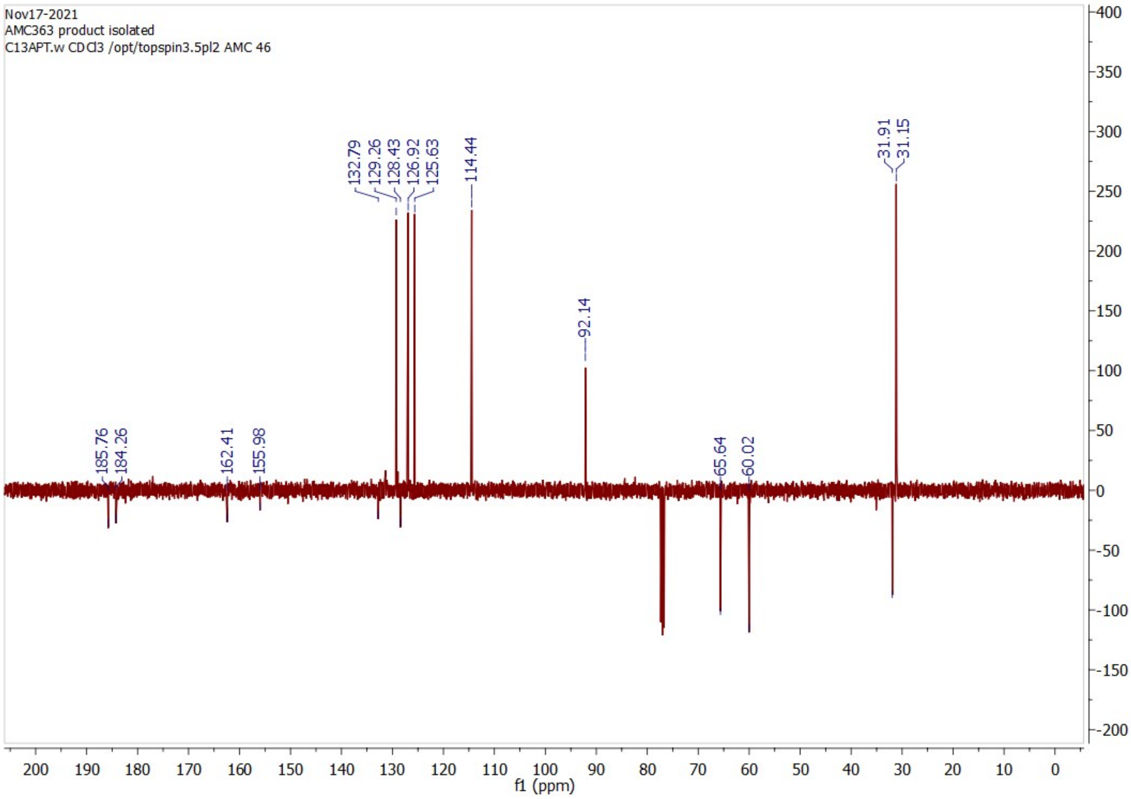

COSY of **1-(4-(*tert*-butyl)phenyl)-3-(4-(3-hydroxypropoxy)phenyl)propane-1,3-dione 12b.**

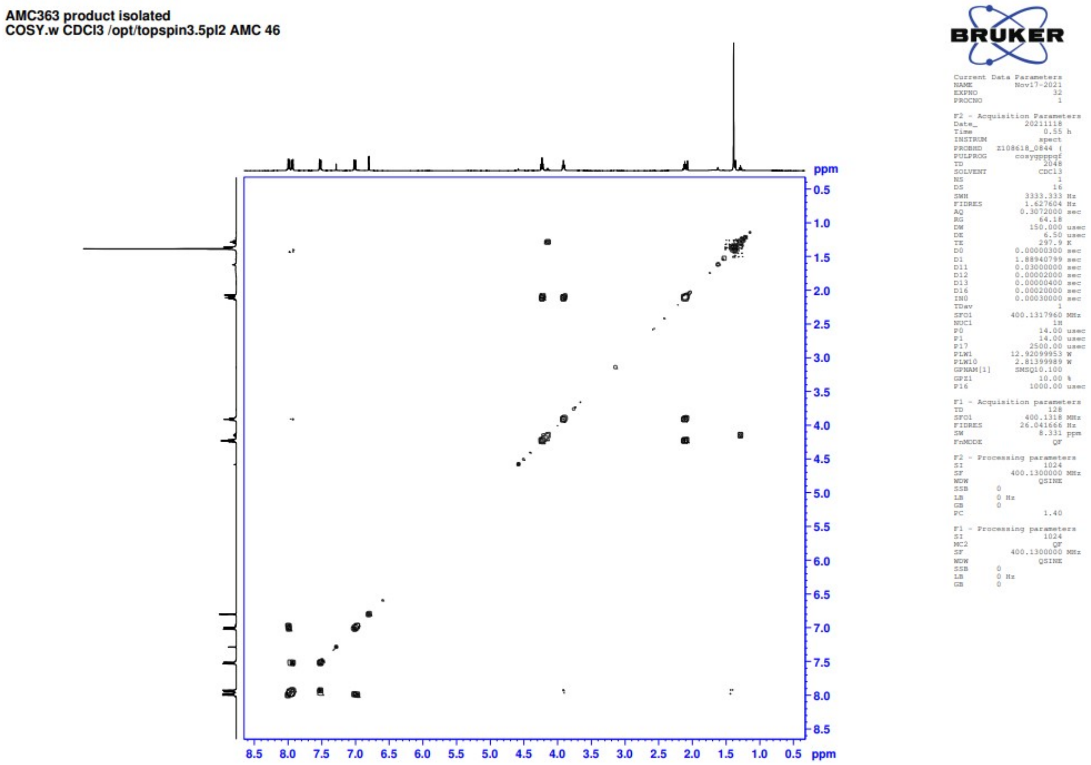

^{1}H NMR (400 MHz, $CDCl_3$) of **1-(4-(3-hydroxypropoxy)phenyl)-3-(4-methoxyphenyl)propane-1,3-dione 12c.**

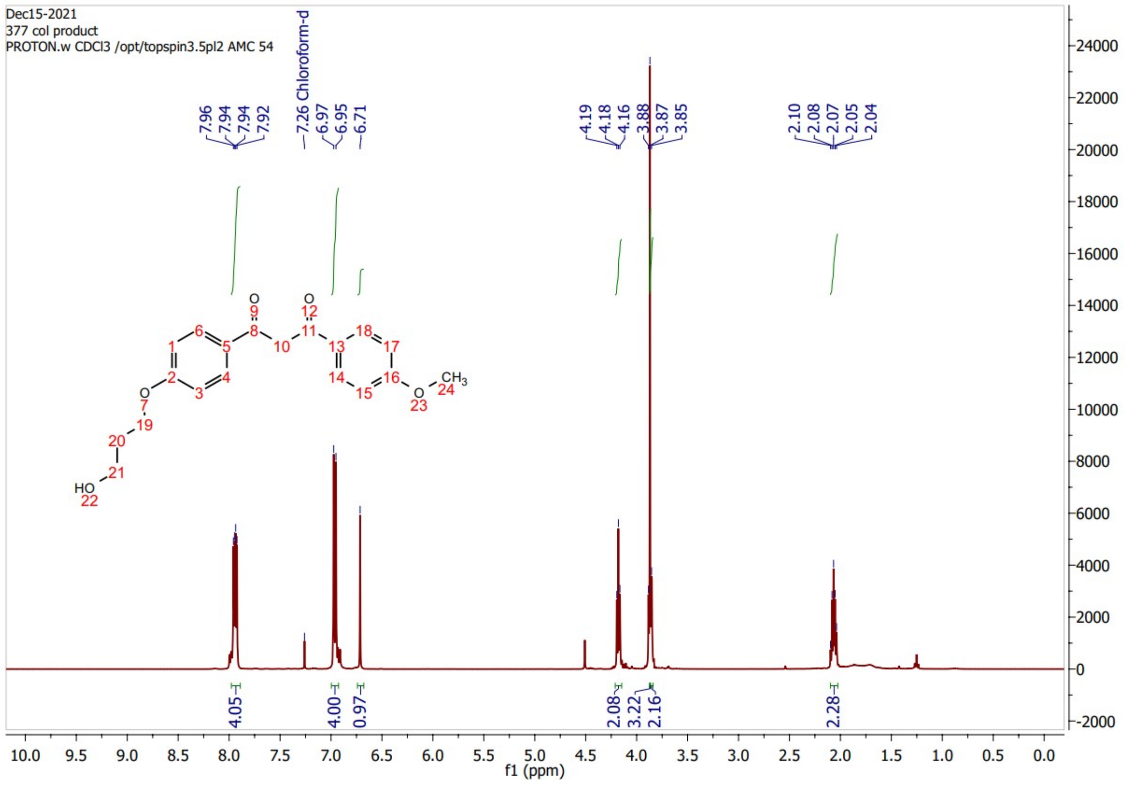

^{13}C NMR (101 MHz, $CDCl_3$) of **of 1-(4-(3-hydroxypropoxy)phenyl)-3-(4-methoxyphenyl)propane-1,3-dione 12c.**

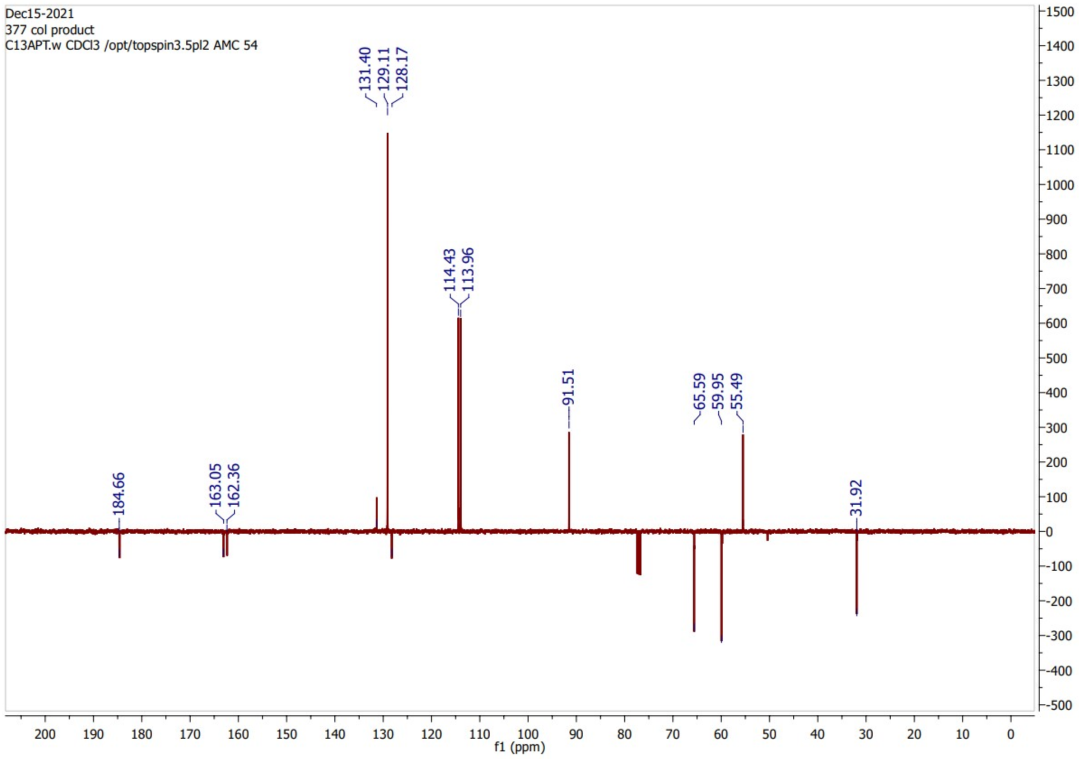

COSY of **1-(4-(3-hydroxypropoxy)phenyl)-3-(4-methoxyphenyl)propane-1,3-dione 12c.**

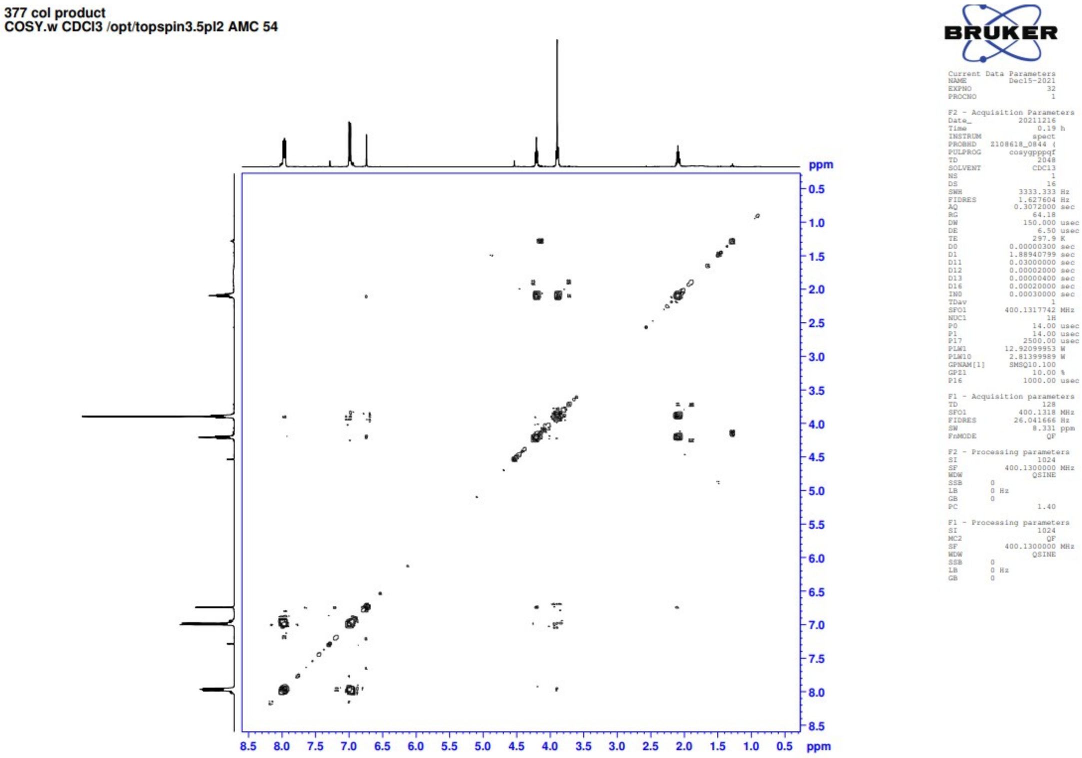

^{1}H NMR (400 MHz, $CDCl_3$) of **1-(4-Chlorophenyl)-3-(4-(3-hydroxypropoxy)phenyl)propane-1,3-dione 12d.**

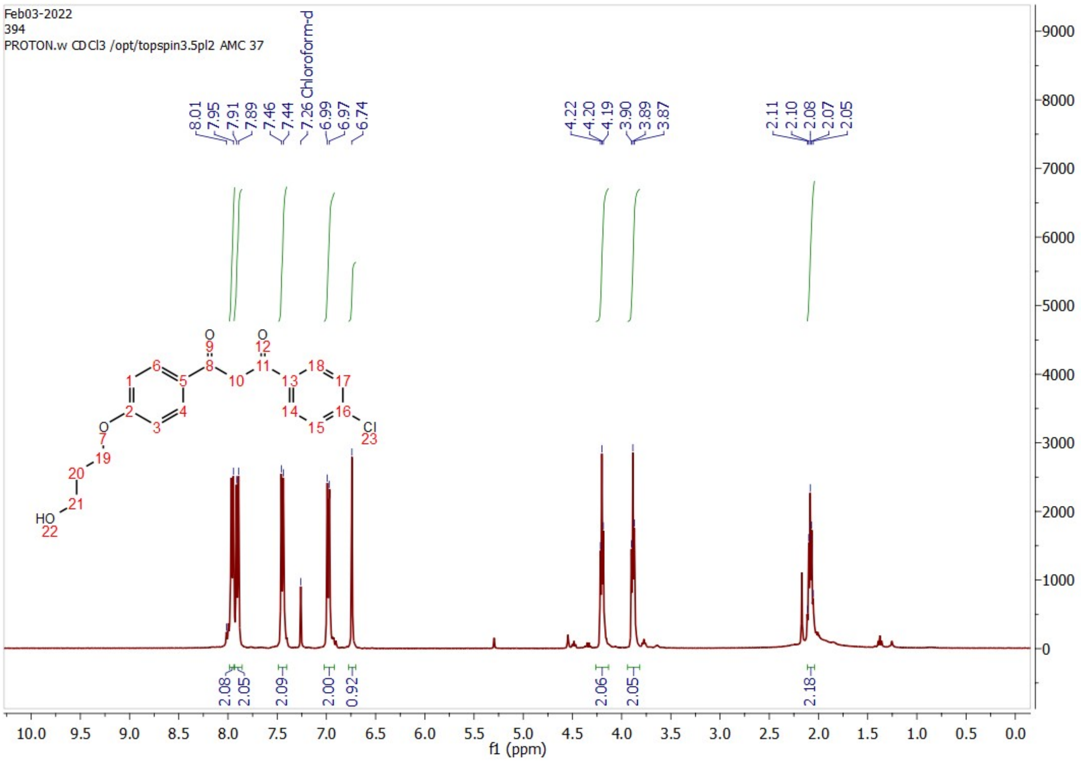

^{13}C NMR (101 MHz, $CDCl_3$) of **1-(4-Chlorophenyl)-3-(4-(3-hydroxypropoxy)phenyl)propane-1,3-dione 12d.**

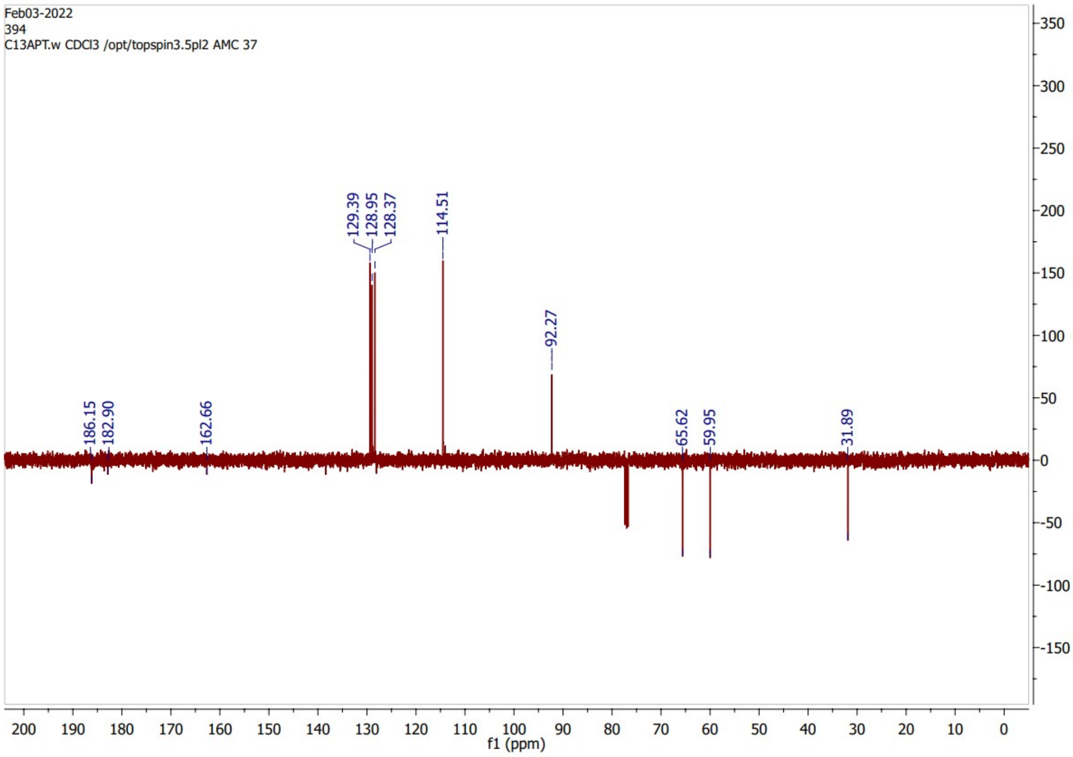

COSY of **1-(4-Chlorophenyl)-3-(4-(3-hydroxypropoxy)phenyl)propane-1,3-dione 12d.**

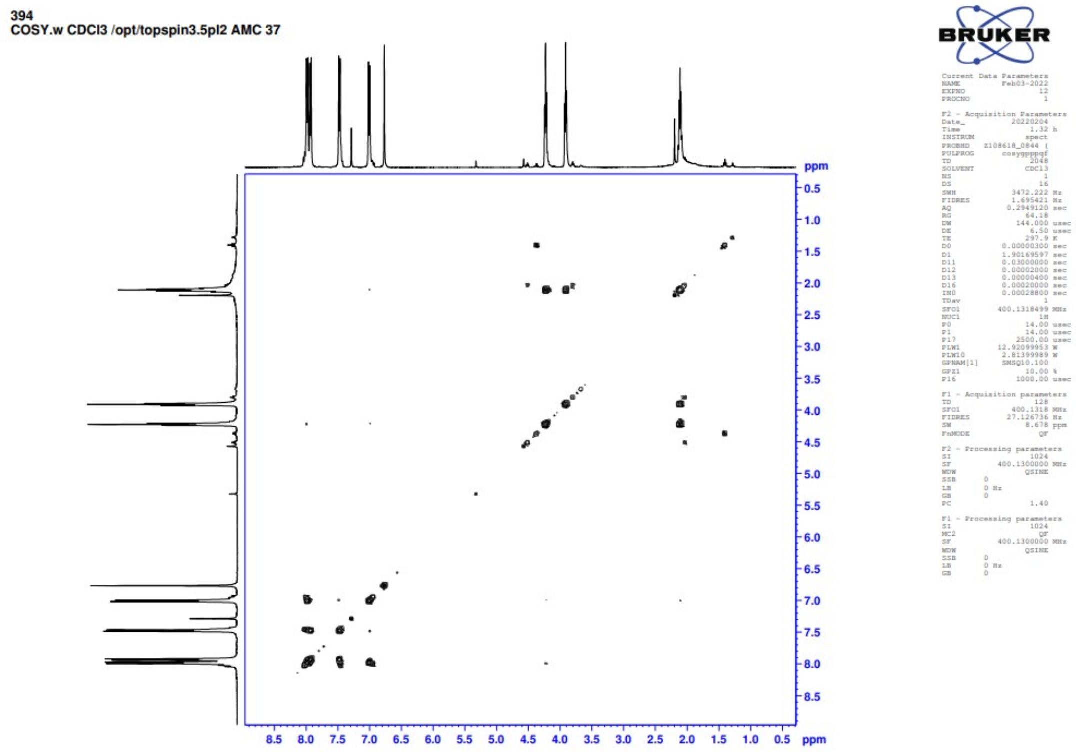

^{1}H NMR (500 MHz, $CDCl_3$) of **2-Cyano-3,3-diphenylacrylic acid 13.**

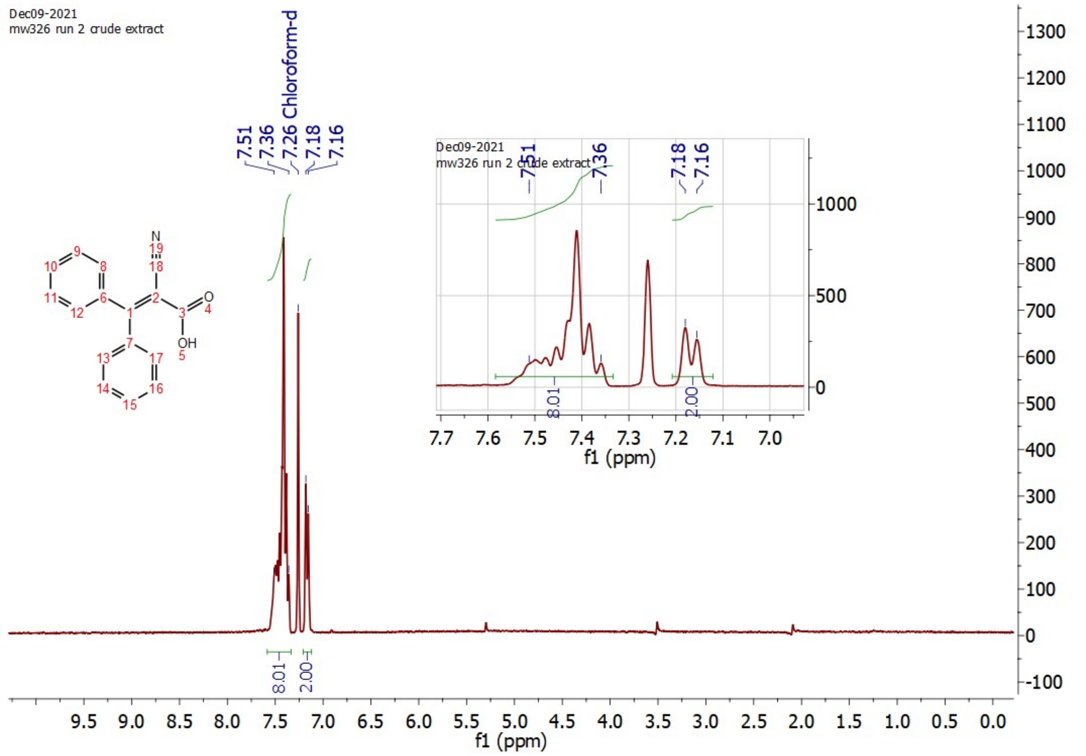

^{13}C NMR (126 MHz, $CDCl_3$) of **2-Cyano-3,3-diphenylacrylic acid 13.**

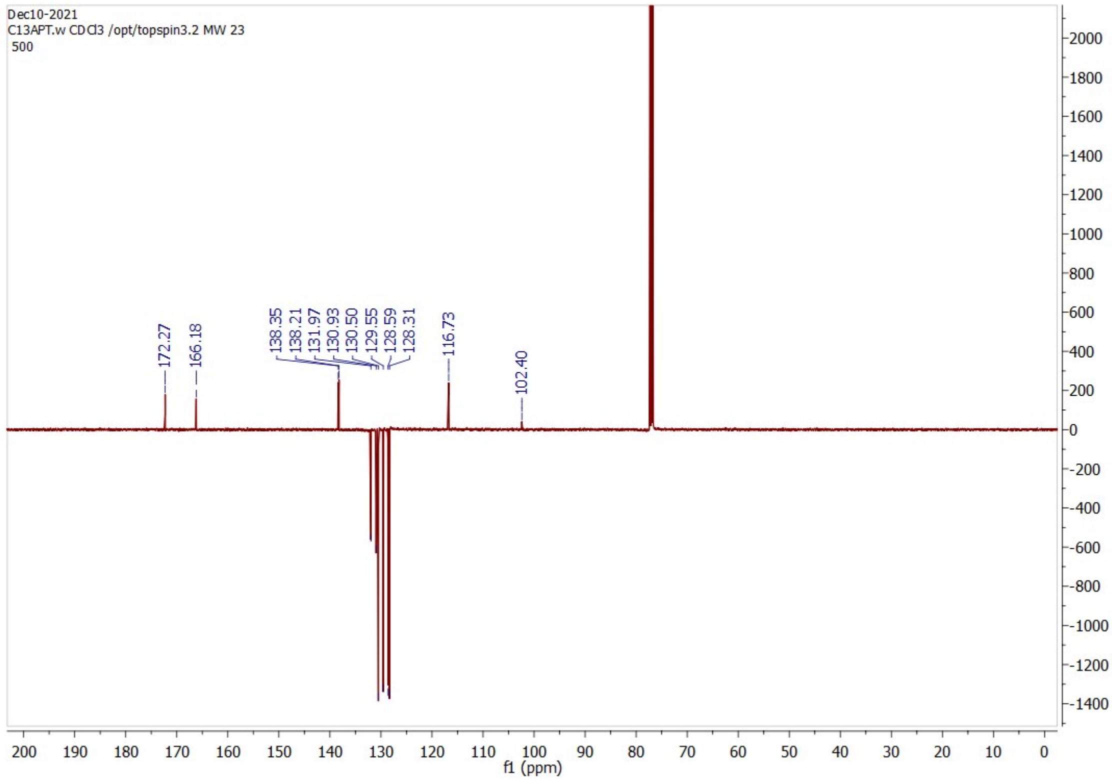

^{1}H NMR (400 MHz, $CDCl_3$) of **methyl 4-methoxybenzoate.**

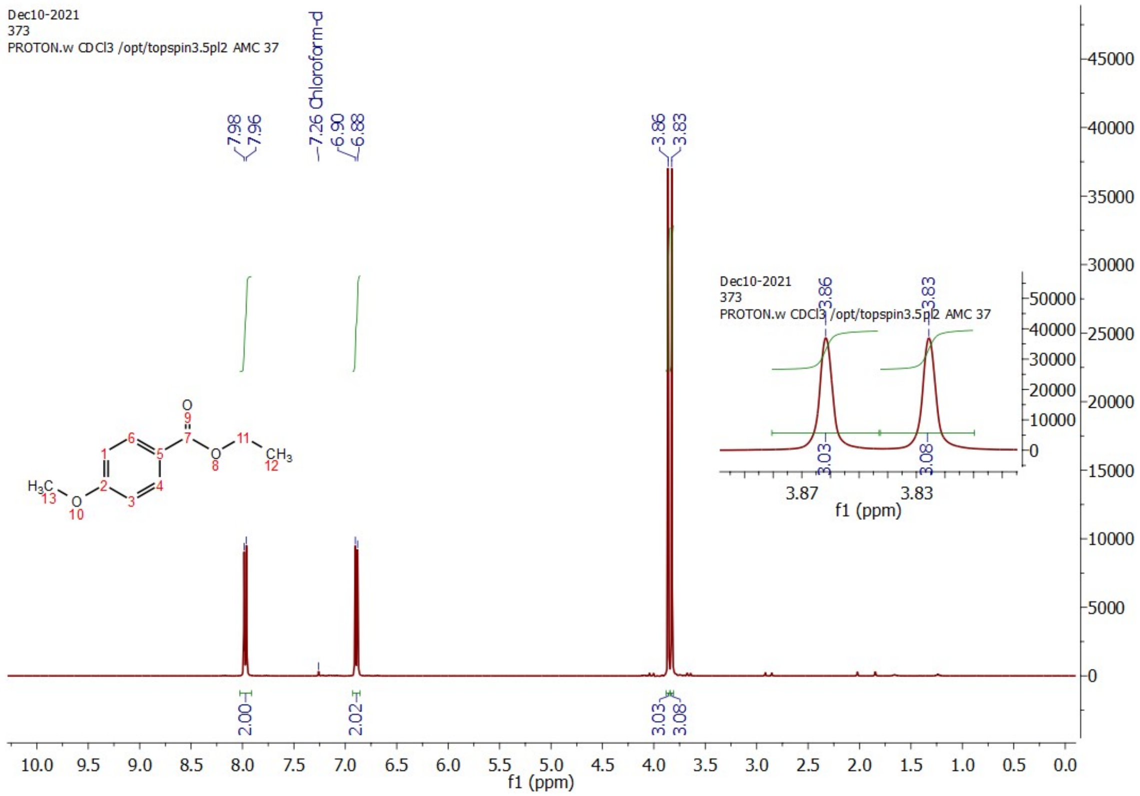

^{1}H NMR (400 MHz, $CDCl_3$) of **3-(4-(3-Oxo-3-phenylpropanoyl)phenoxy)propyl 2-cyano-3,3-diphenylacrylate, 10a.**

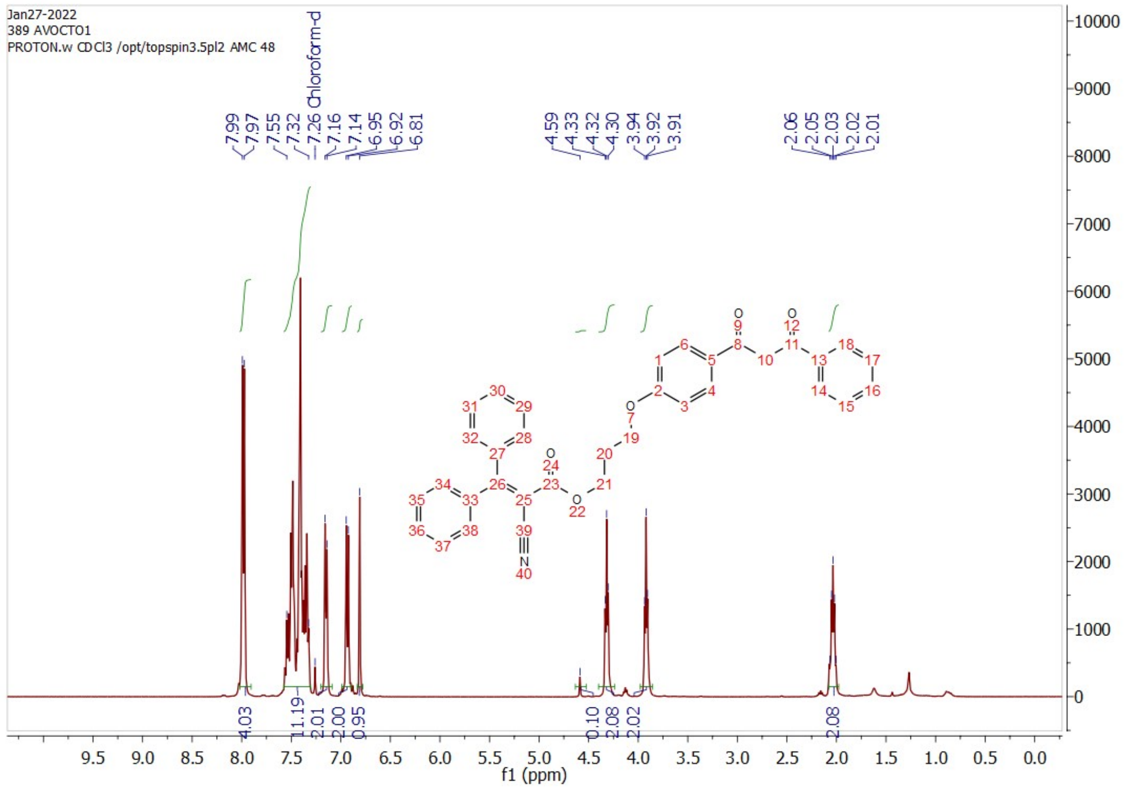

^{13}C NMR (101 MHz, $CDCl_3$) of **3-(4-(3-Oxo-3-phenylpropanoyl)phenoxy)propyl 2-cyano-3,3-diphenylacrylate, 10a**

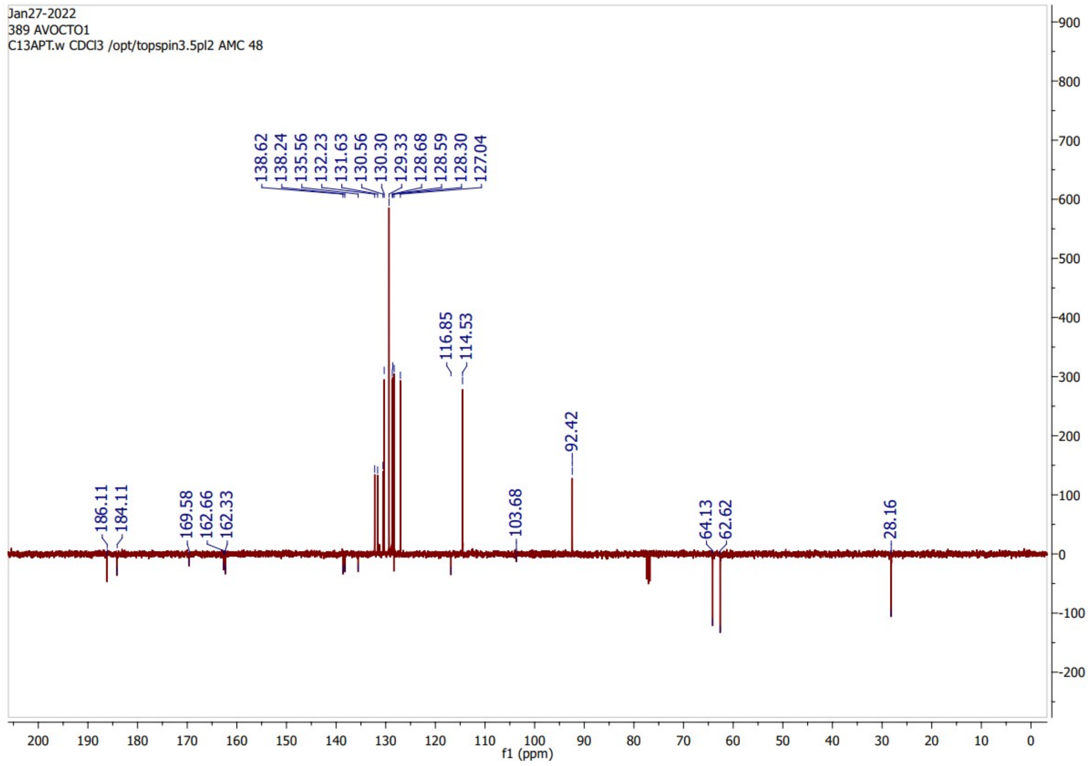

COSY of **3-(4-(3-Oxo-3-phenylpropanoyl)phenoxy)propyl 2-cyano-3,3-diphenylacrylate, 10a**

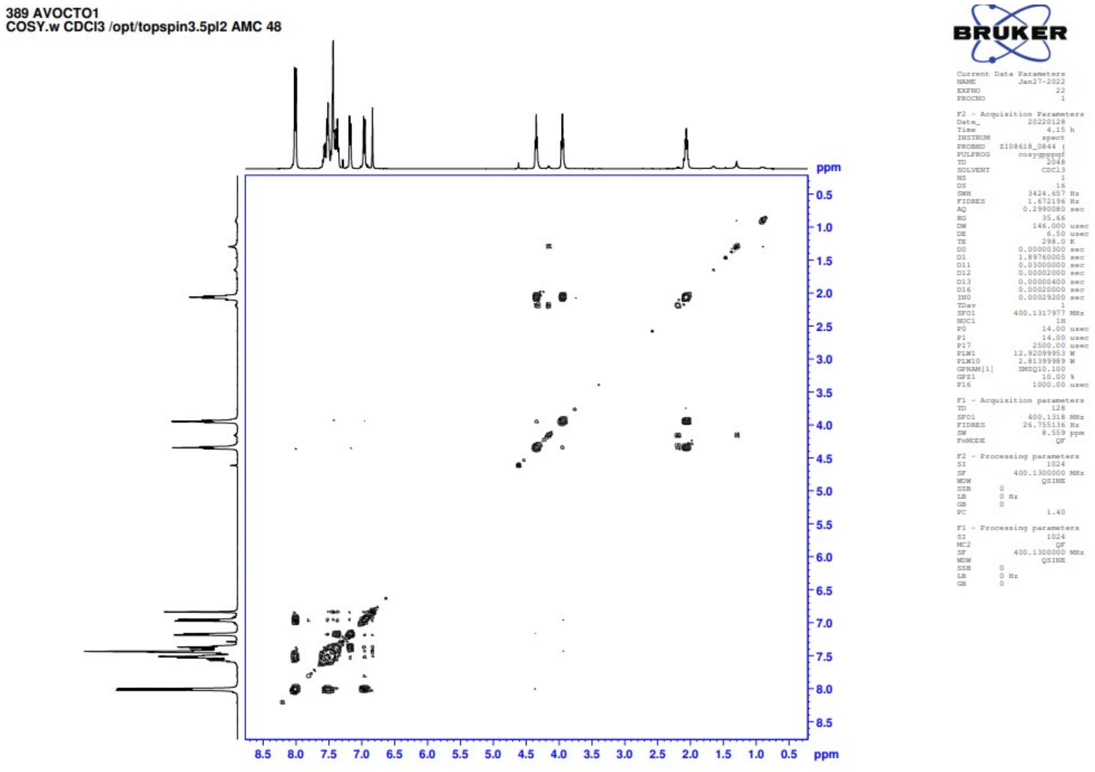

^{1}H NMR (400 MHz, $CDCl_3$) of **3-(4-(3-(4-(*tert*-butyl)phenyl)-3-oxopropanoyl)phenoxy)propyl 2-cyano-3,3-diphenylacrylate, 10b.**

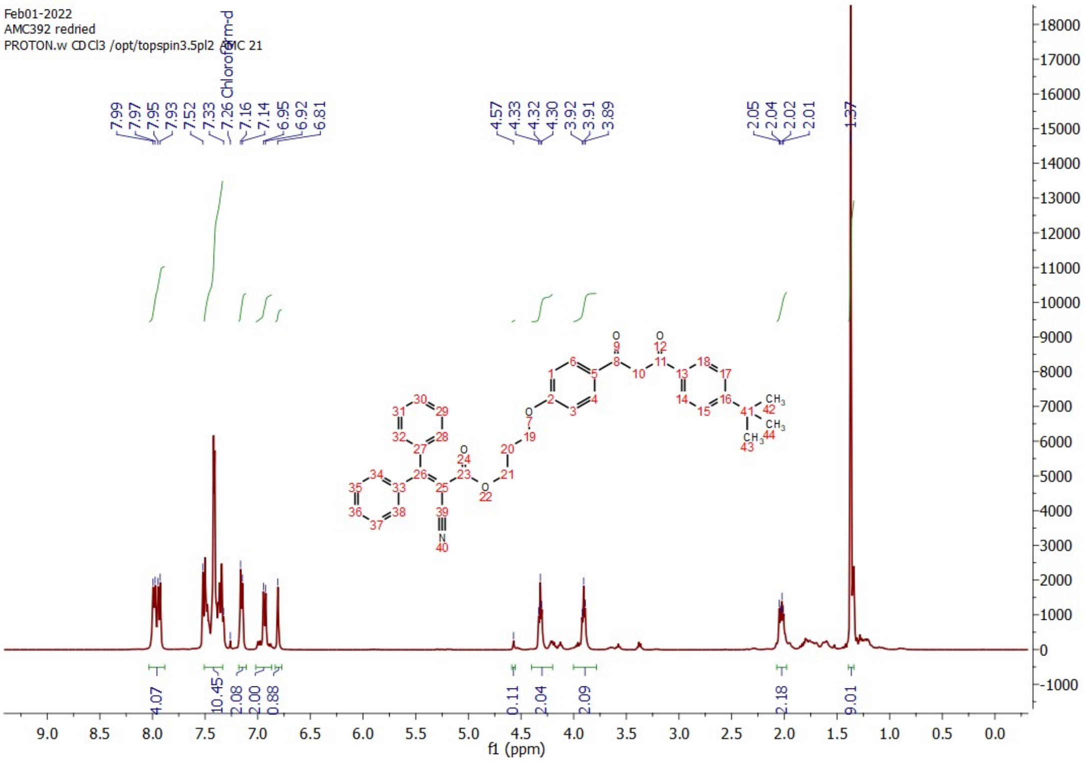

^{13}C NMR (101 MHz, $CDCl_3$) of **3-(4-(3-(4-(*tert*-butyl)phenyl)-3-oxopropanoyl)phenoxy)propyl 2-cyano-3,3-diphenylacrylate, 10b**

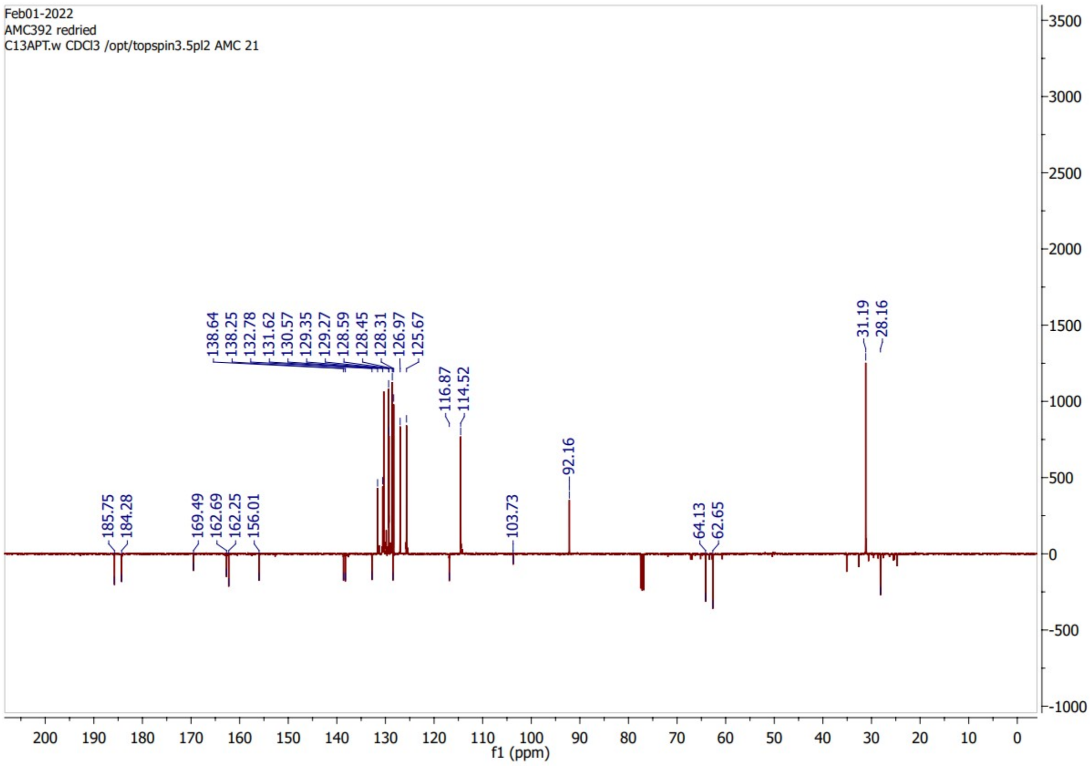

COSY of **3-(4-(3-(4-(*tert*-butyl)phenyl)-3-oxopropanoyl)phenoxy)propyl 2-cyano-3,3-diphenylacrylate, 10b.**

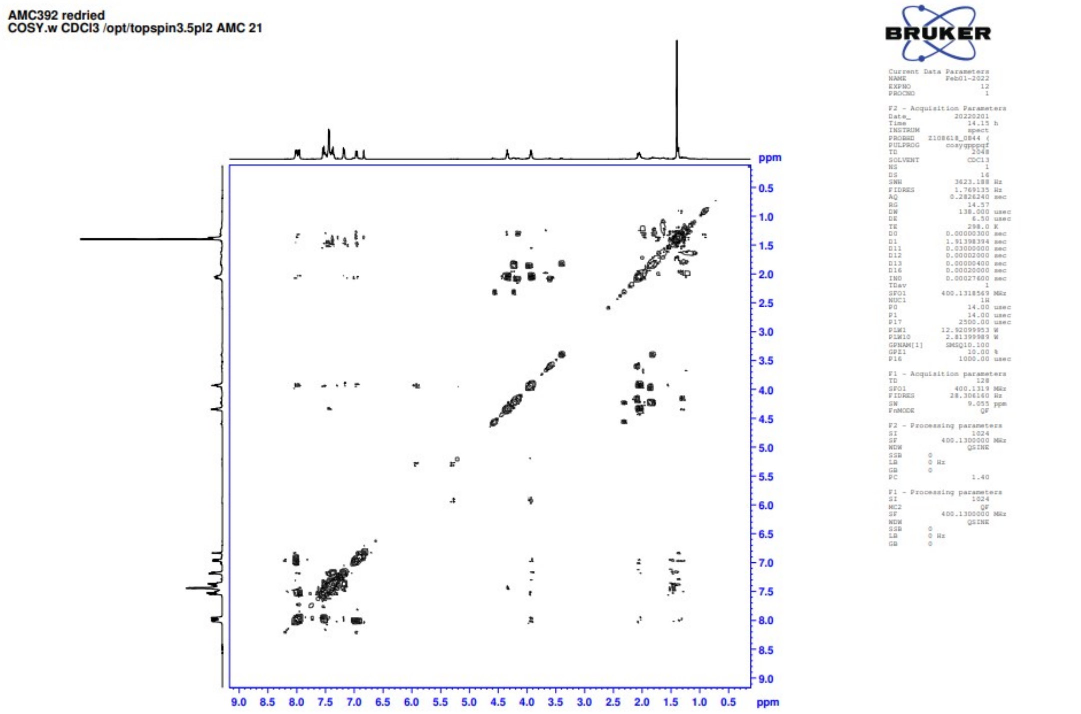

^{1}H NMR (400 MHz, $CDCl_3$) of **3-(4-(3-(4-Methoxyphenyl)-3-oxopropanoyl)phenoxy)propyl 2-cyano-3,3-diphenylacrylate 10c.**

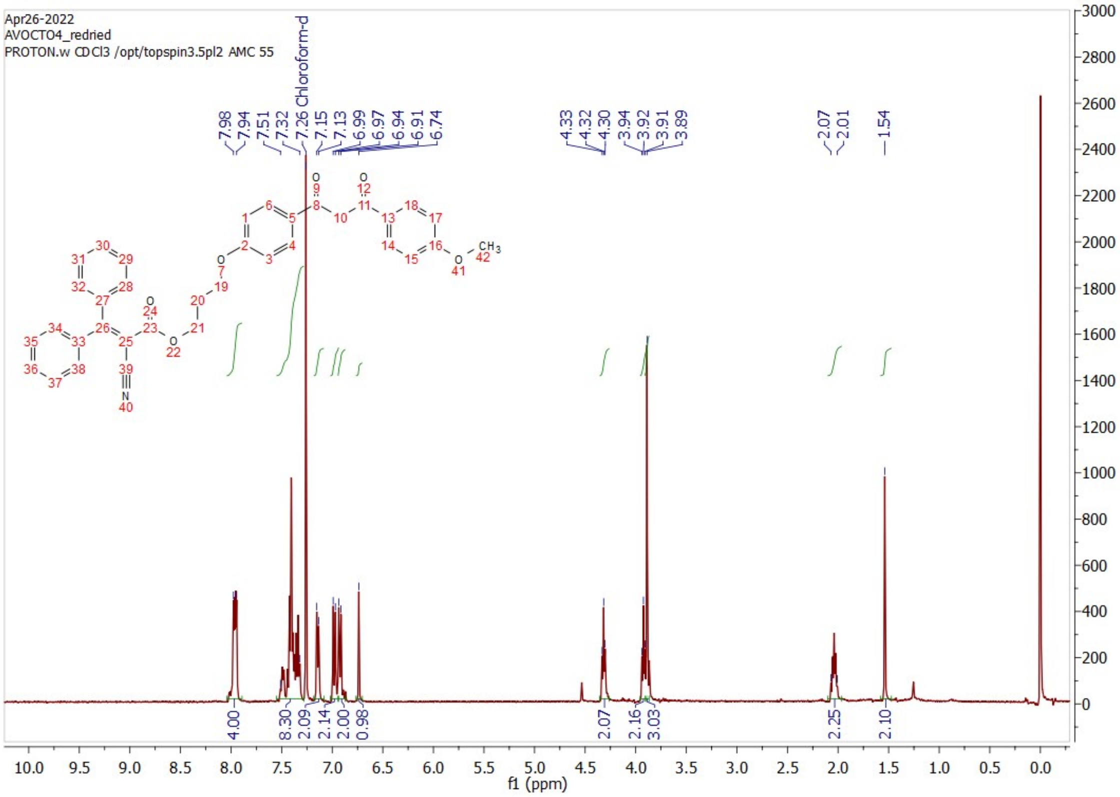

^{13}C NMR (101 MHz, $CDCl_3$) of **3-(4-(3-(4-Methoxyphenyl)-3-oxopropanoyl)phenoxy)propyl 2-cyano-3,3-diphenylacrylate 10c**

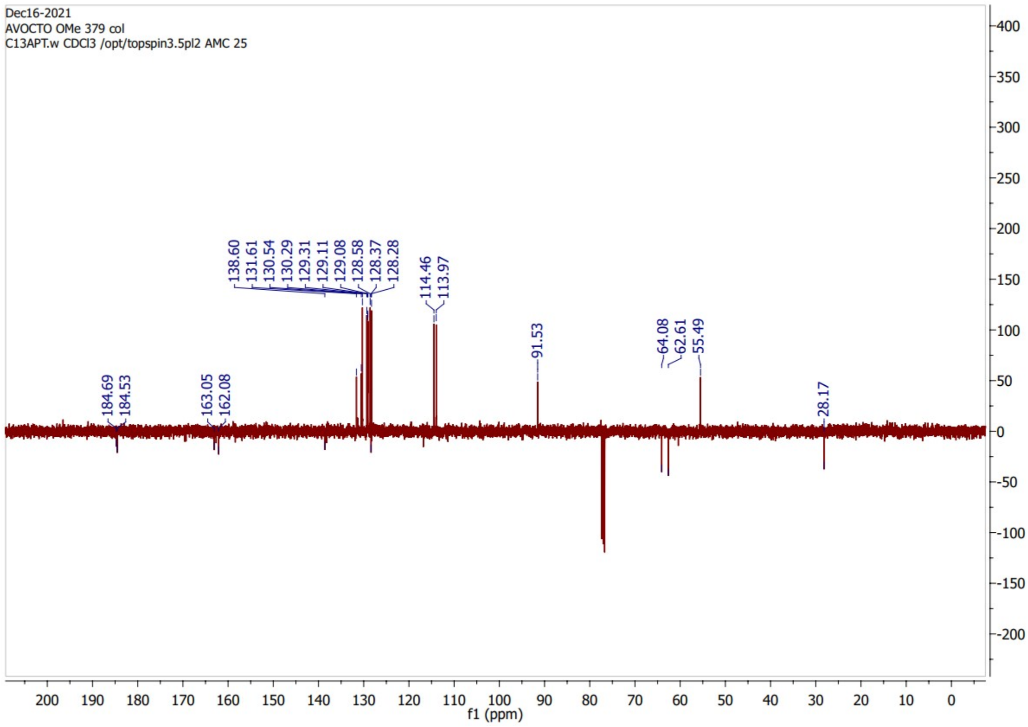

COSY of **3-(4-(3-(4-Methoxyphenyl)-3-oxopropanoyl)phenoxy)propyl 2-cyano-3,3-diphenylacrylate 10c**

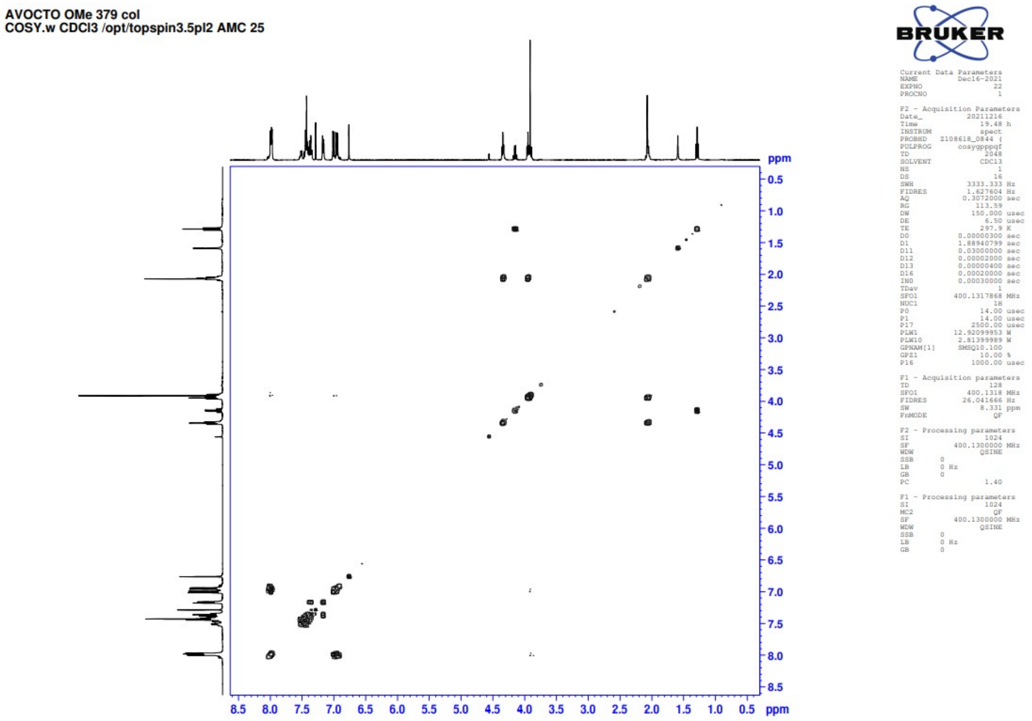

^1H NMR (400 MHz, $CDCl_3$) of **3-(4-(3-(4-Chlorophenyl)-3-oxopropanoyl)phenoxy)propyl 2-cyano-3,3-diphenylacrylate, 10d.**

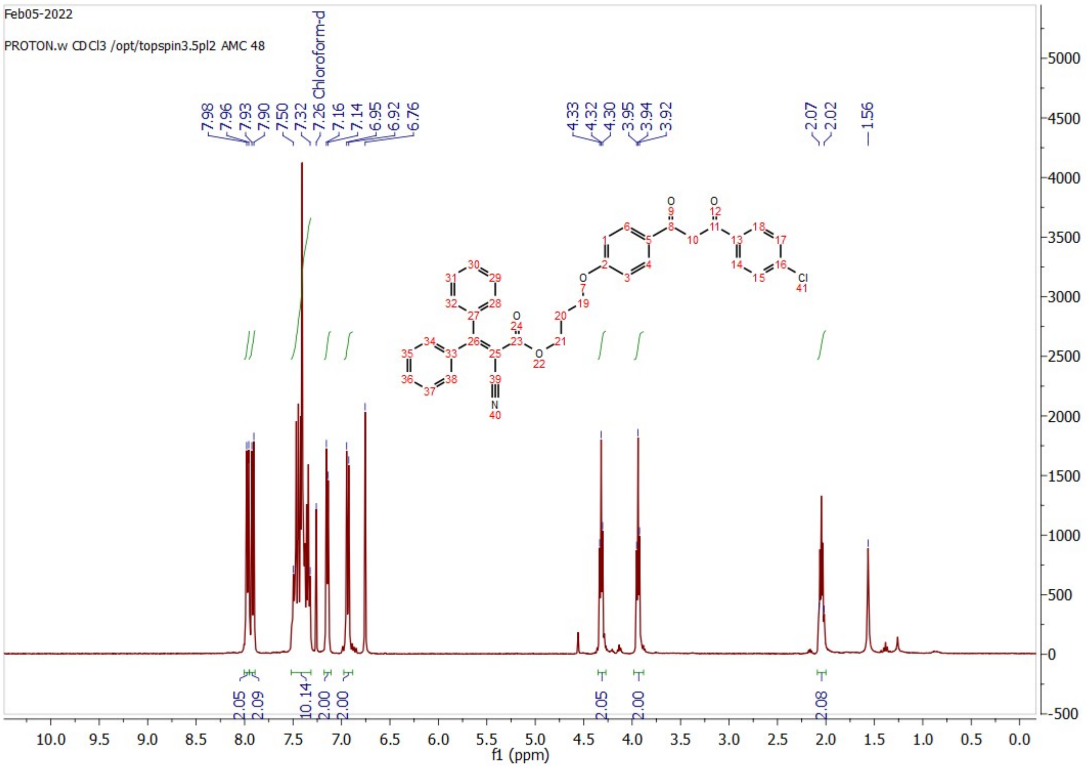

^{13}C NMR (101 MHz, $CDCl_3$) of **3-(4-(3-(4-Chlorophenyl)-3-oxopropanoyl)phenoxy)propyl 2-cyano-3,3-diphenylacrylate, 10d.**

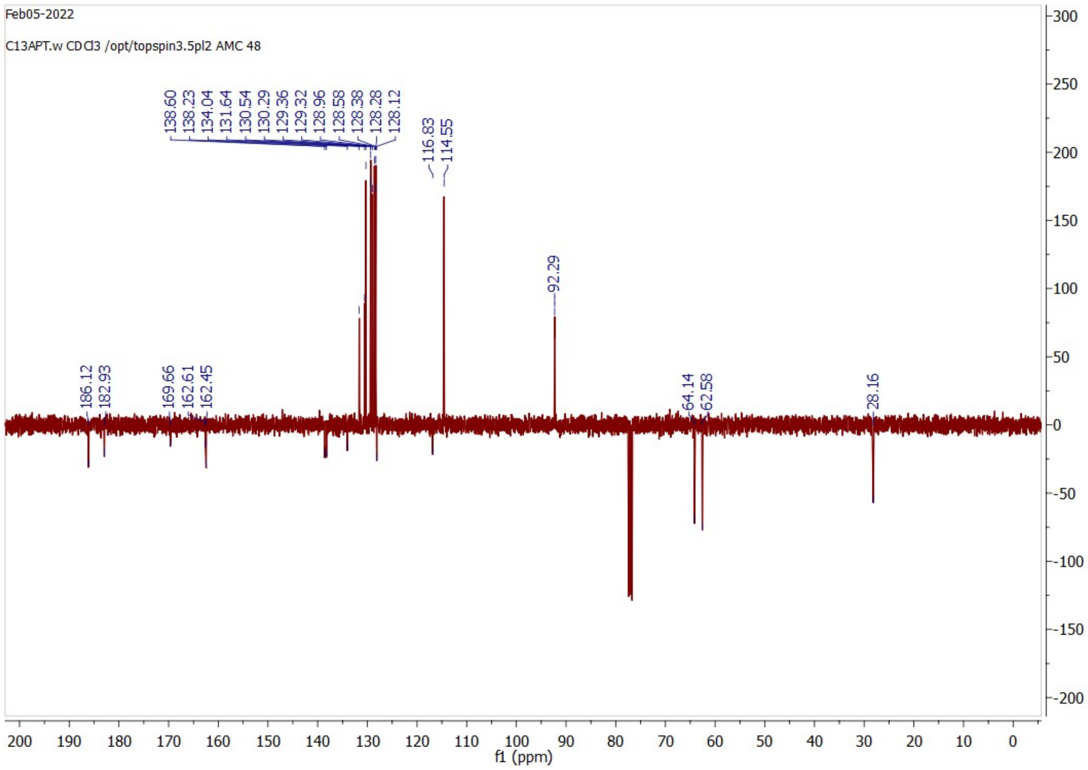

COSY of **3-(4-(3-(4-Chlorophenyl)-3-oxopropanoyl)phenoxy)propyl 2-cyano-3,3-diphenylacrylate, 10d.**

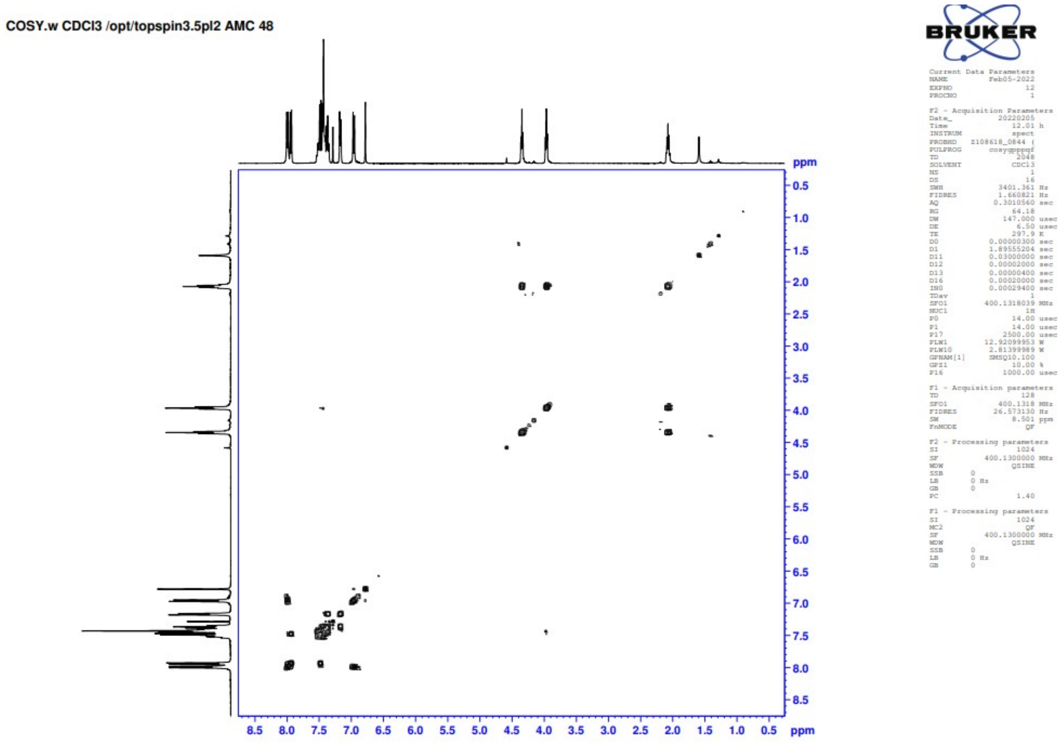

^{1}H NMR (400 MHz, $CDCl_3$) of **3-(4-(2-Methyl-3-oxo-3-phenylpropanoyl)phenoxy)propyl 2-cyano-3,3-diphenylacrylate, 10e.**

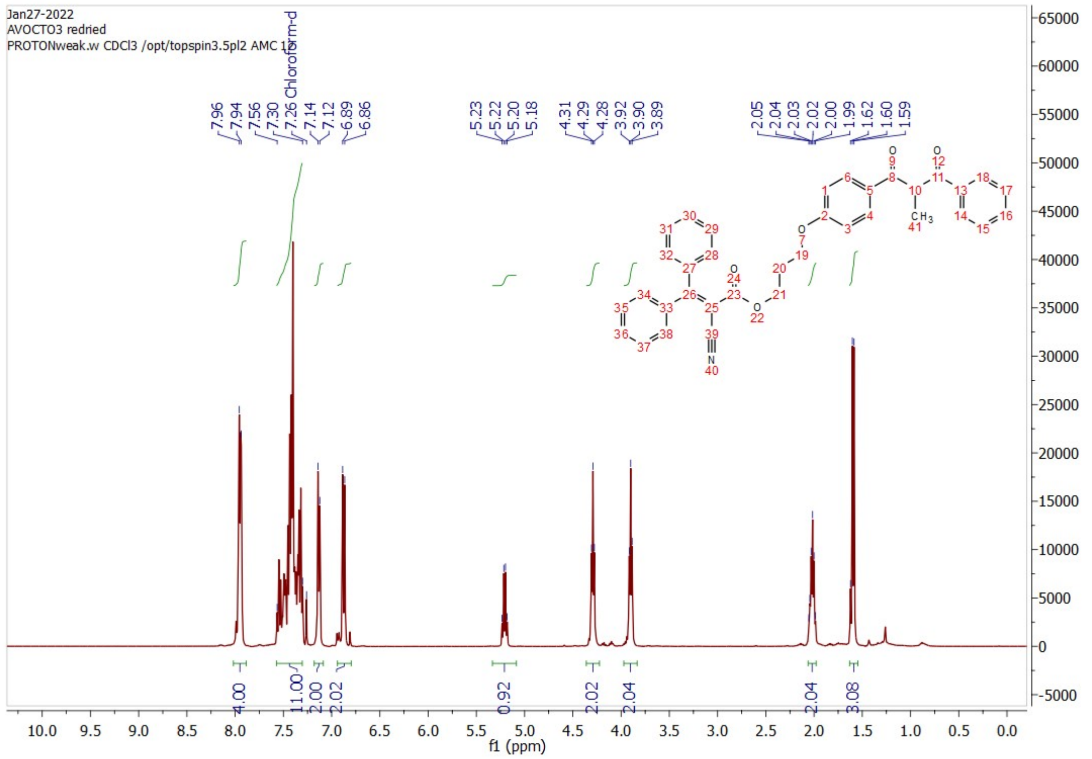

^{13}C **NMR** (101 MHz, $CDCl_3$) of **3-(4-(2-Methyl-3-oxo-3-phenylpropanoyl)phenoxy)propyl 2-cyano-3,3-diphenylacrylate, 10e.**

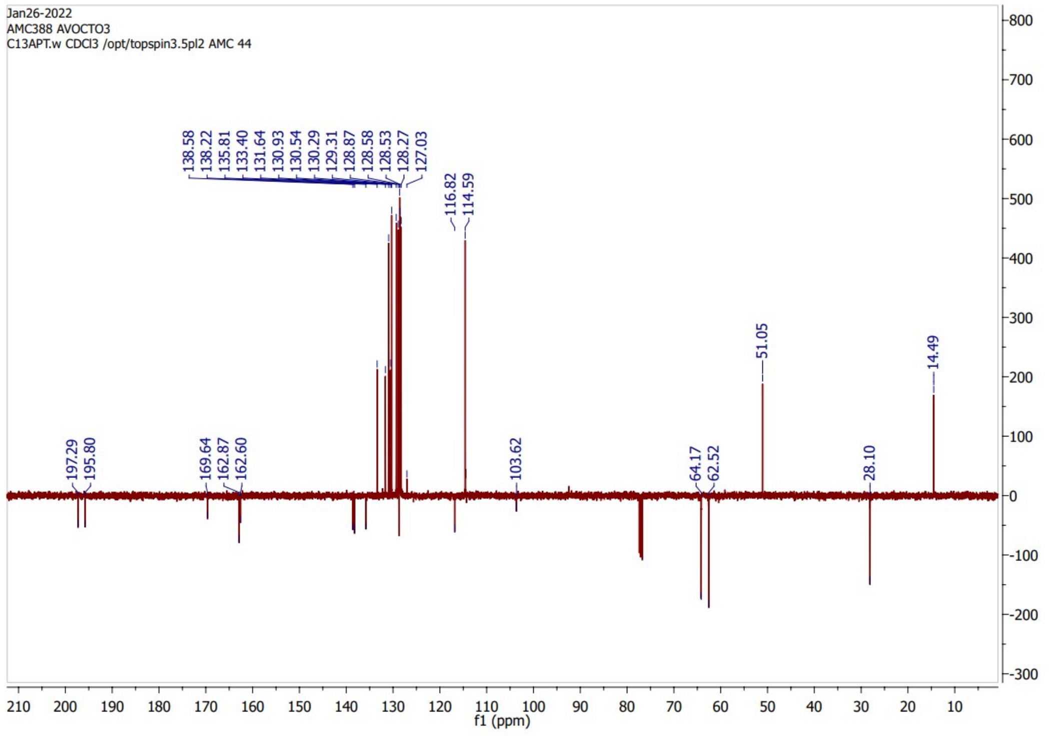

COSY of **3-(4-(2-Methyl-3-oxo-3-phenylpropanoyl)phenoxy)propyl 2-cyano-3,3-diphenylacrylate, 10e.**

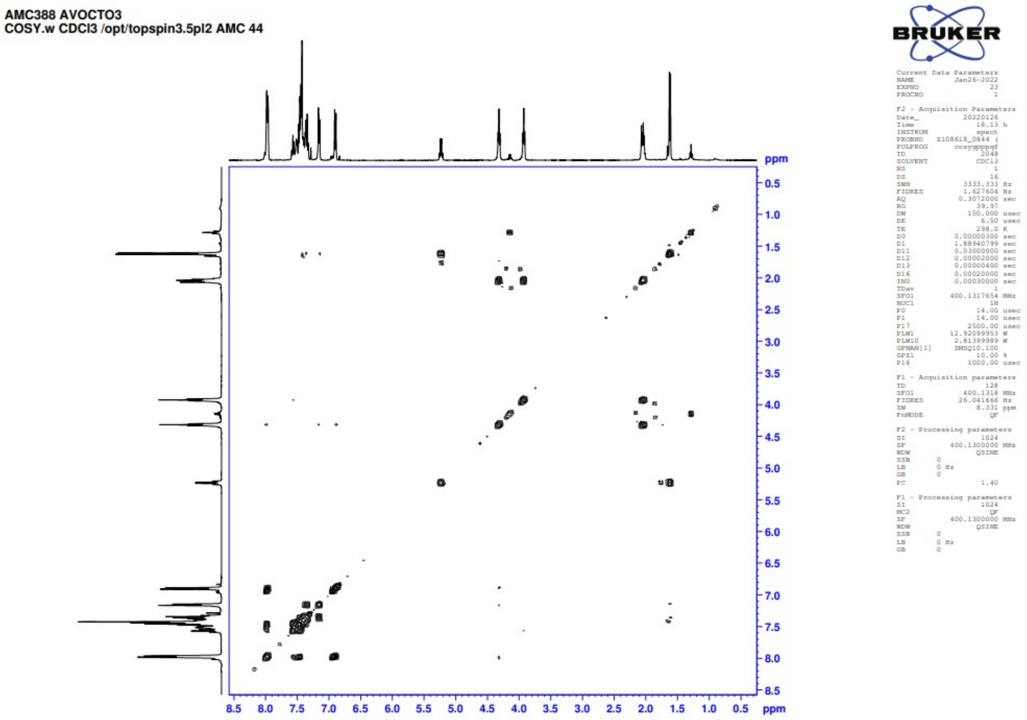

^{1}H NMR (400 MHz, $CDCl_3$) of **1-(4-Chlorophenyl)-3-(4-methoxyphenyl)propane-1,3-dione.**

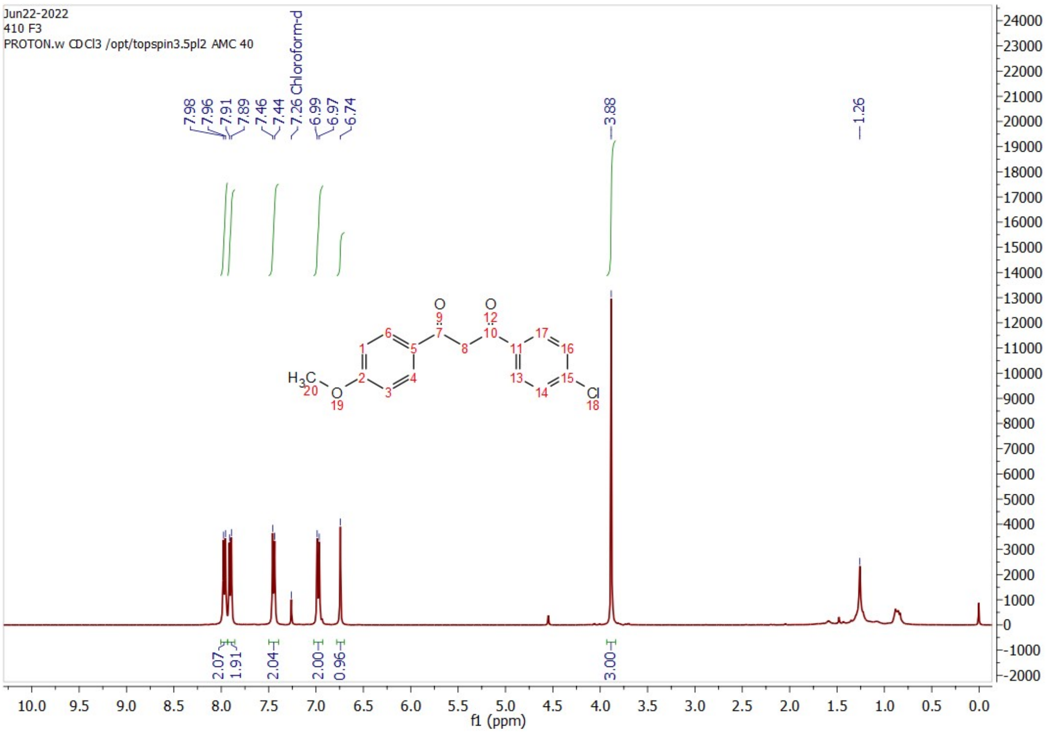

^{1}H NMR (400 MHz, $CDCl_3$) of **1-(4-(Tert-butyl)phenyl)-3-(4-methoxyphenyl)-2-methylpropane-1,3-dione.**

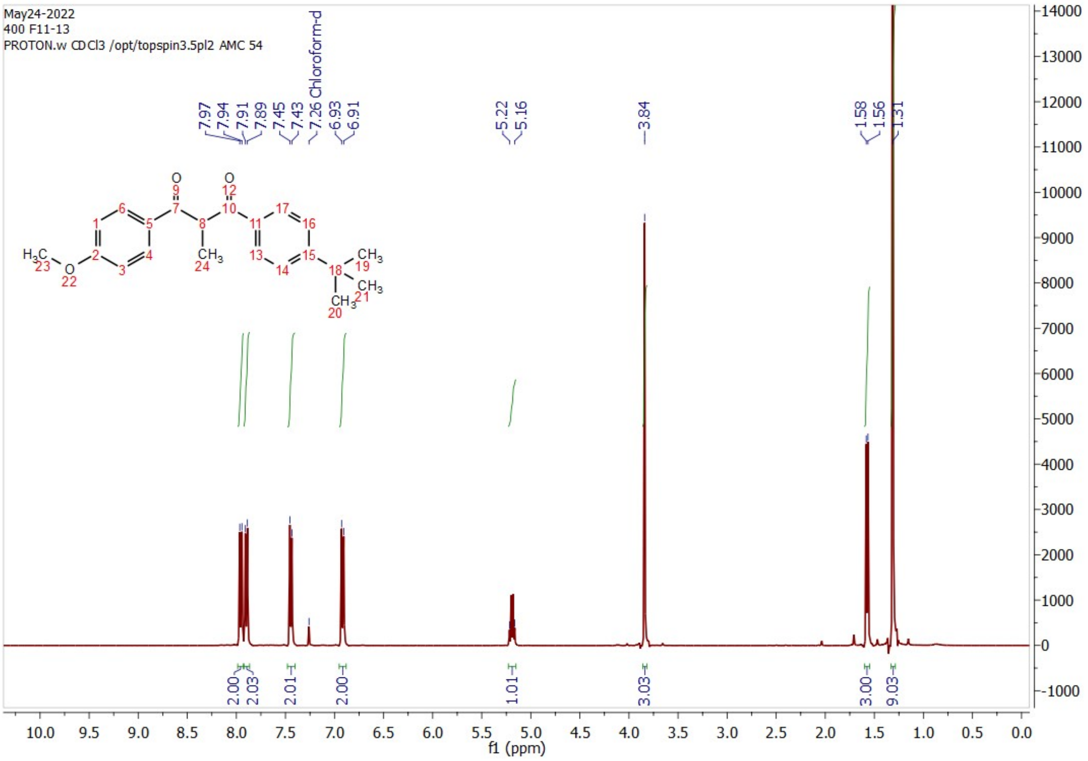

^{13}C NMR (101 MHz, $CDCl_3$) of **1-(4-(*tert*-butyl)phenyl)-3-(4-methoxyphenyl)-2-methylpropane-1,3-dione.**

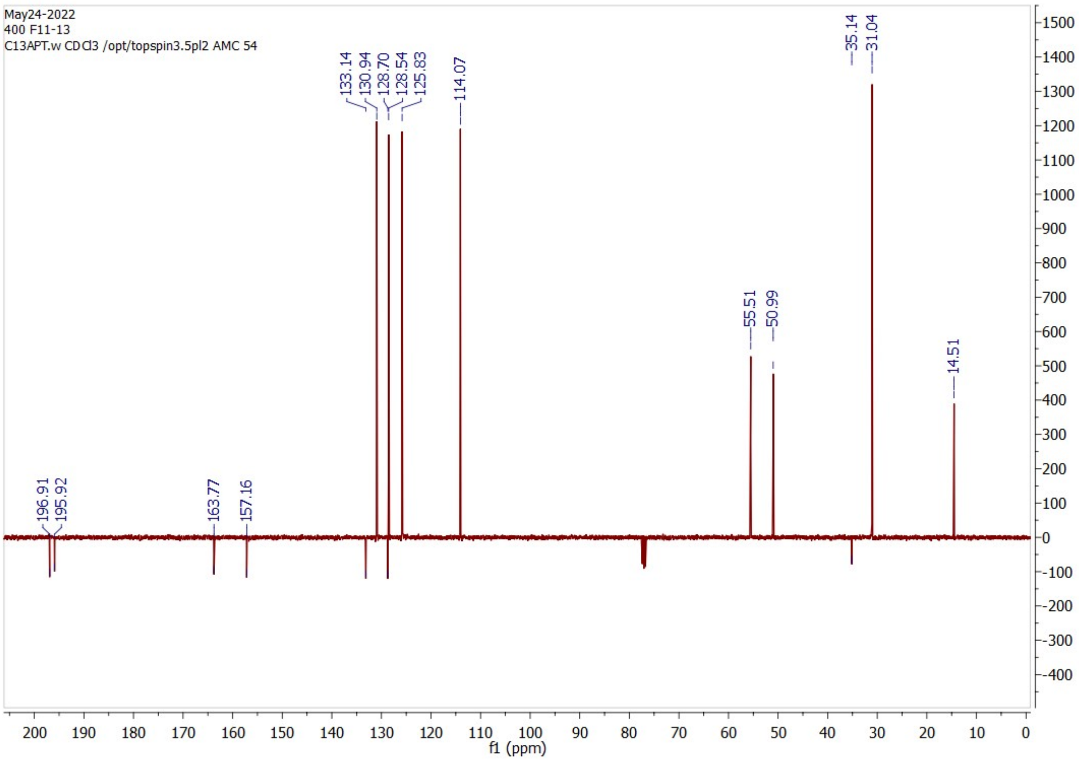

^{1}H NMR (400 MHz, $CDCl_3$) of **(E)-3-(4-acetoxy-3,5-dimethoxyphenyl)acrylic acid 13.**

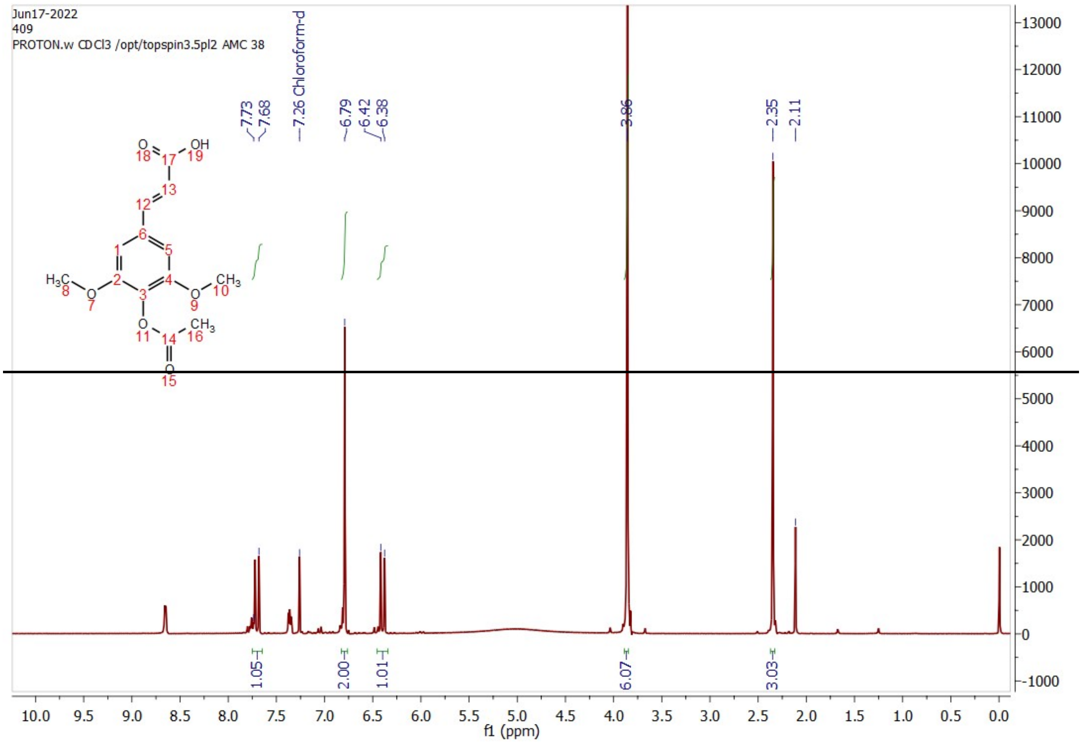

^{1}H NMR (400 MHz, $CDCl_3$) of **3-(4-(3-oxo-3-phenylpropanoyl)phenoxy)propyl (E)-3-(4-acetoxy-3,5-dimethoxyphenyl)acrylate 14b.**

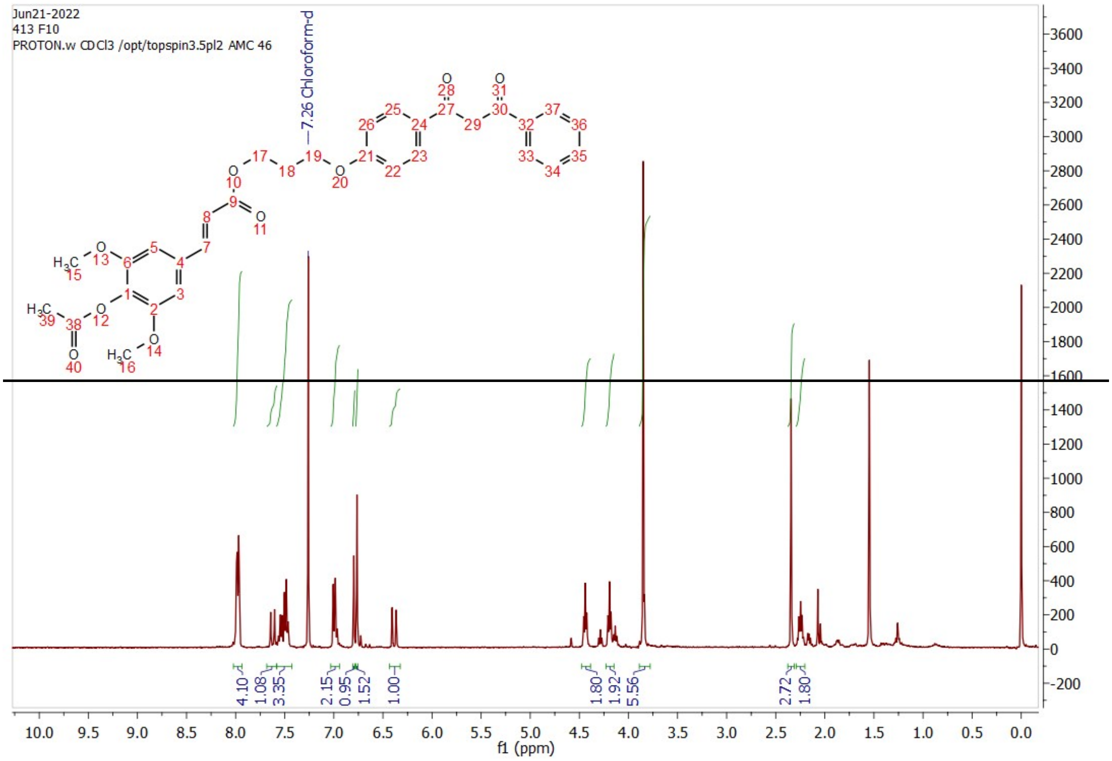

Detail of ^{1}H NMR (400 MHz, $CDCl_3$) of **3-(4-(3-oxo-3-phenylpropanoyl)phenoxy)propyl (E)-3-(4-acetoxy-3,5-dimethoxyphenyl)acrylate 14a:14b 2:1.**

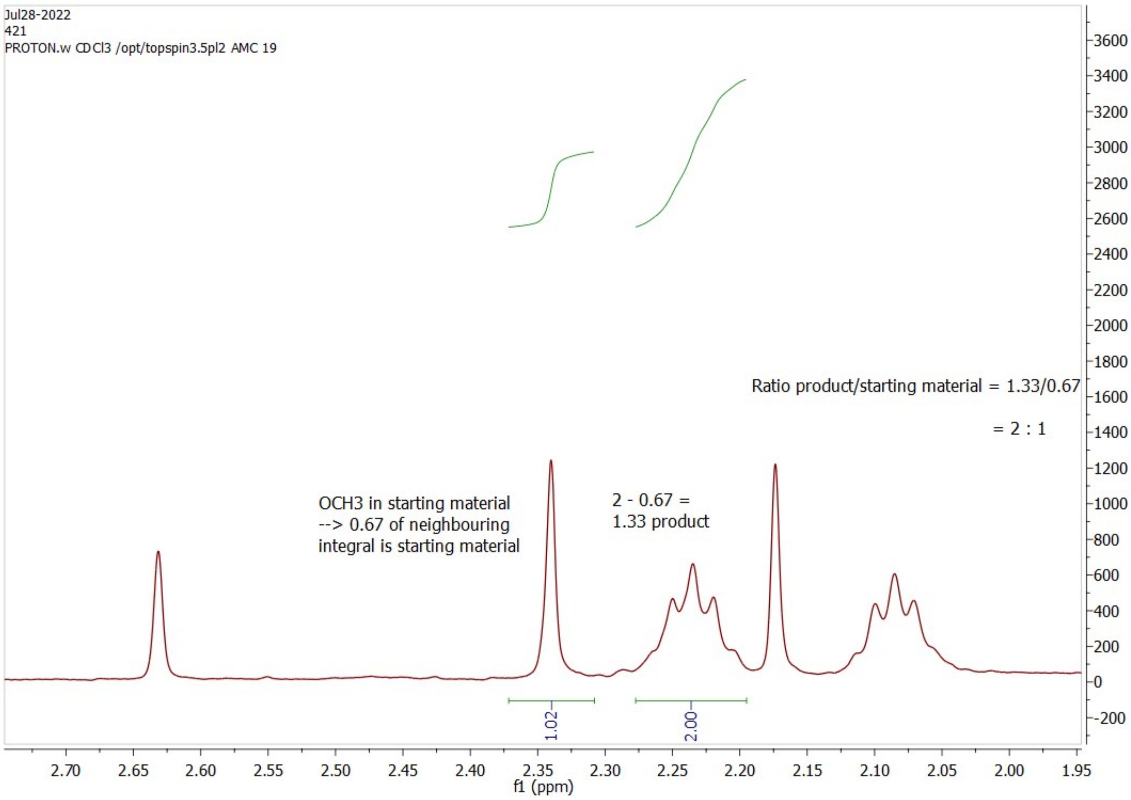

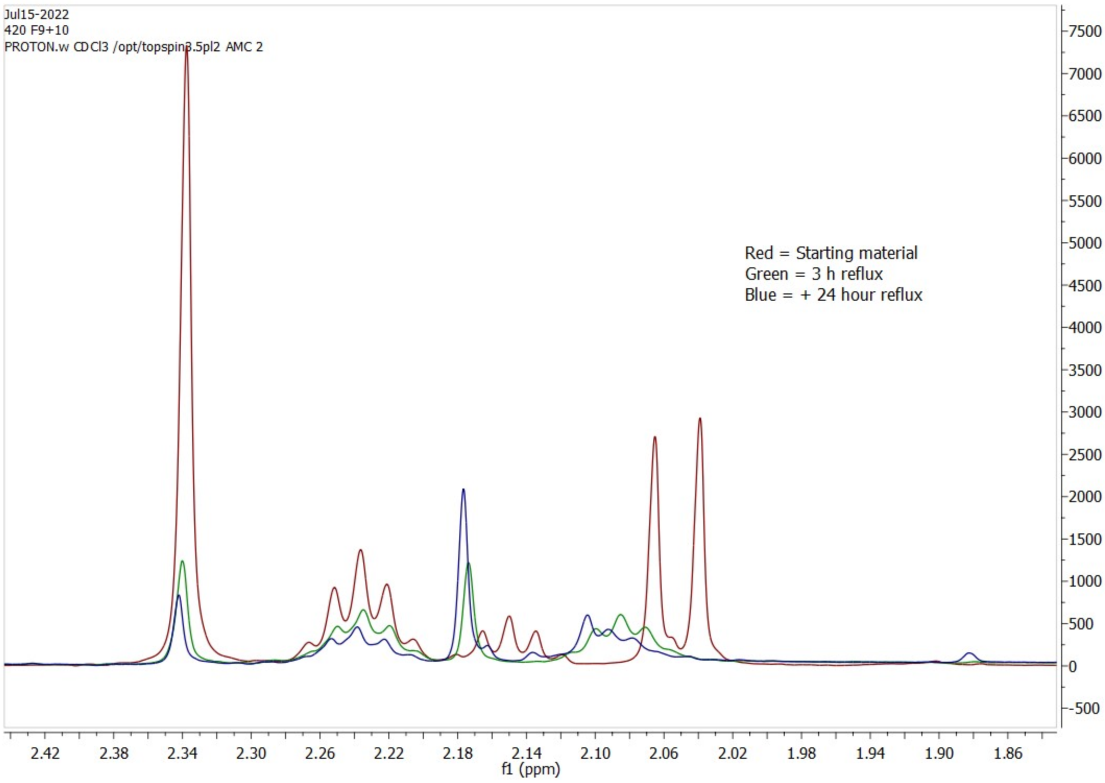

Comparison of 3 hour reflux and 24 hour reflux to starting material ^{1}H NMR

3) Experimental Details: Spectroscopy.

'Ultraslow' Spectroscopies

Long term (5 mins < t < 2 hours) and steady-state i.e., static photostability measurements (UV-vis) were obtained using samples of ~µM concentrations in HPLC grade solvent (i.e., absolute ethanol (VMR chemicals) or HPLC grade acetonitrile). Samples were prepared in High Precision quartz cuvettes (Hellma Analytics) of 10 x 10 mm pathlength containing 2 mLs of each solution. For photostability studies, concentrations were adjusted in the relevant solvent to give a stable absorption between 0.6-0.8 before irradiation. To simulate the response under near-to-life conditions, solar simulator irradiations were carried out using a solar simulator (LCS-100, Newport) with an AM1.5G spectral correction filter to generate 1 SUN irradiance i.e. the distance of the cuvette from the lamp was adjusted to equate to 1,000 W/m^2 (100 mW/cm^2) sustained over 2 hours which is equivalent to the Sun's energy at Earth's surface (see Figure S6). Spectra were recorded on a UV-vis spectrometer (Cary 60 UV-vis, Agilent Technologies) at specified intervals up to 2 hours at a scan rate of 600 nm/min at 1 nm intervals with baseline correction.

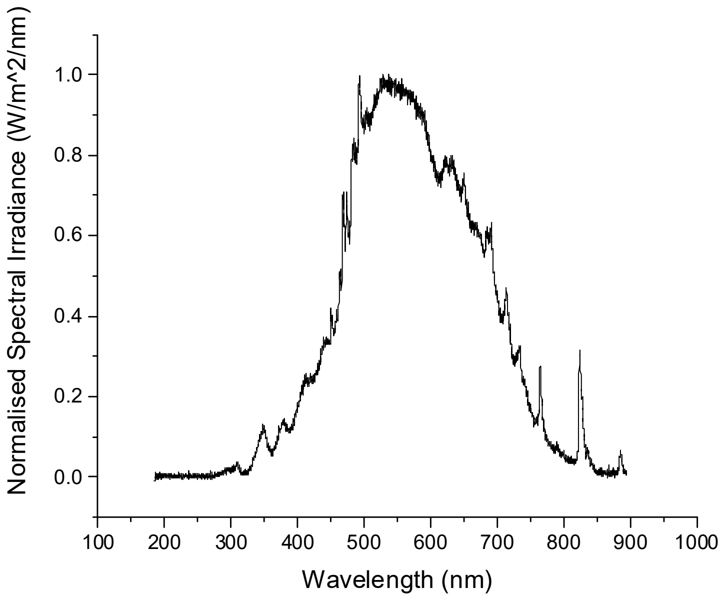

Figure S6. The normalised spectrum obtained of the LCS-100 solar simulator used in the irradiation studies.

Quantifying photostability

A key measurement of photostability is the percentage degradation of a molecule under the action of UV or solar light, and this can be quantified by equation (5):

$$\frac{C(I) - C(F)}{C(I)} x\,100 \qquad (5)$$

where *C*(*I*) is the initial concentration before irradiation, *C*(*F*) is the concentration after irradiation. The concentration can be directly converted into absorption by Beer's law and therefore the difference in concentration is a direct measure of the change in absorbance. In terms of sunscreens, a low percentage degradation (<10% change in activity) is preferred and ideally complete photostability is approached in all solvents. In one study with photoprotective lipsticks by Maier et al, over 5% loss of activity for a UV exposure of 12.5 standard erythema doses (SED) was determined to be a threshold below which a sunscreen can be considered photostable.[2] Percentage recovery, i.e. once the sample is returned to the dark, in the case of avobenzone would indicate re-appearance of the enol form after irradiation has ceased and the sample had been moved to the dark. Recovery of the UV absorption could indicate that the molecular integrity of the chemical filter has recovered by re-formation of the original molecule; while incomplete recovery indicates that irreversible processes have taken place that have led to permanent loss of the parent molecule. An area-under-curve ratio (*AUC R*), which is a ratio of the final area (AUC_F) and the initial area (AUC_I) for the irradiation can be calculated by equation (6):

$$AUCI = \frac{AUC_F}{AUC_I} \qquad (6)$$

An *AUC R* > 0.80 has previously been used as a criterion for a photostable molecule.[39] The AUC R ratio has been converted into a percentage for the Tables in the main paper.

Transient Electronic Absorption Spectroscopy (TEAS).

The transient electronic absorption setup at the Warwick Centre for Ultrafast Spectroscopy has been described previously[40] and so only a brief description is given here with details pertaining to the experiments within this work. 1 mM of

AVOCTO1 **10a**, AVOCTO4 **10d**, AVOCTO5 **10e** and avobenzone in acetonitrile were continuously circulated through a demountable liquid cell (Harrick's Scientific) using a diaphragm pump (SIMDOS). The pathlength of the sample was 100 μm which was achieved by sandwiching 100 μm spacers between CaF_2 windows (front 1 mm and back 2 mm). As described in the main manuscript, two pump wavelengths were used for AVOCTO1 (1: 290 nm 2: 350 nm) and AVOCTO4 **10d** (1: 285 nm 2: 355 nm) and one pump wavelength was used for AVOCTO5 **10e** (280 nm) and avobenzone (355 nm). The power at all pump wavelengths was ~500 μW and the beam diameter at the sample was ~400 μm. The probe pulse, a white light continuum spanning 320-720 nm, was generated by focussing the fundamental 800 nm onto a vertically translating CaF_2 window. The pathlength of the probe pulse was varied by a gold retroreflector mounted onto a delay stage resulting in time delays between -1 ps and 3 ns. Every other pump pulse was blocked by a chopper spinning at 500 Hz. This enabled the pump-on and pump-off absorbance to be directly compared with a resultant output of difference in optical density (ΔOD).

Solvent alone transients were also acquired by pumping acetonitrile at 355 nm set to a higher power of 1 mW. This was done to enable our instrument response to be determined which is the limiting time resolution of our experiment. The acquired transient absorption spectra (TAS) of each molecule were chirp-corrected using the software package KOALA and the data were fit using a global parallel kinetic model using the software package Glotaran.[41]

4) Stabilisation results of avobenzone and binary mixtures with octocrylene.

Avobenzone (Eusolex® 9020 from Merck) was tested for stability in ethanol, acetonitrile, cyclohexane and DMSO at µM concentrations using UV-vis spectroscopy as described in the below. A range of solvents was chosen to reflect the complex environment in which sunscreen active ingredients are found. Ethanol was chosen as it is a typical solvent in the cosmetics industry, where it is used to solubilise ingredients with aqueous solubility. Acetonitrile is a polar solvent and in contrast to ethanol is non-protic. Cyclohexane is chosen as it is nonpolar and can reflect the environment of the oil-phase of a sunscreen formulation in terms of polarity.

UV-visible properties of avobenzone in solution

The UV-vis properties of avobenzone in the absence of any other additives are shown in Figure S7. Two major absorption bands are visible in the spectrum. The higher energy band centred around 275_nm is due to the diketo form, as in other open-chain 1,3-diketones. The lower energy band centred around 355_nm is due to the chelated enol forms that are in rapid thermal equilibrium.

There is a small shift to shorter wavelengths in the less polar solvent cyclohexane versus acetonitrile and ethanol. The lower energy absorbance of the enol form relative to the diketo form can be explained by increased conjugation, the stabilising effect of the intramolecular hydrogen bond and π delocalisation in the enol form. The more intense absorption of the enol band is due to a symmetry allowed transition – a bonding electron in a π-orbital is excited to an anti-bonding π*-orbital giving rise to the high intensity (ππ*) band.[42]

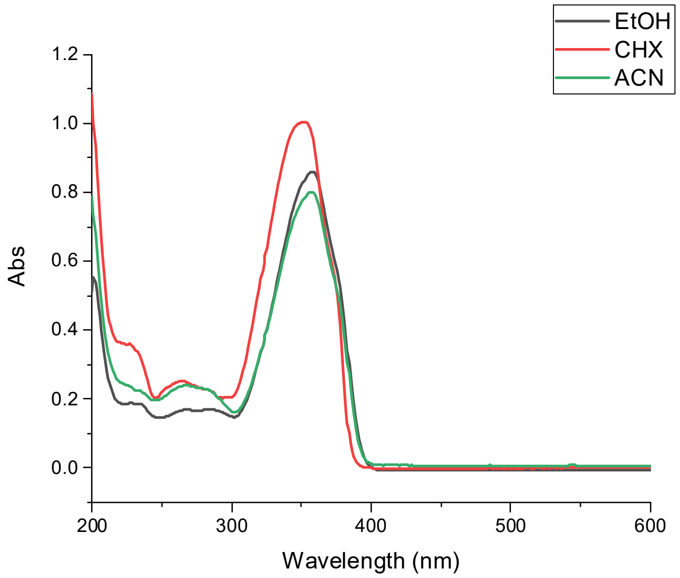

Figure S7. UV-vis spectra of avobenzone **1** in three solvents of varying polarity:

a) EtOH:Ethanol (λ_{max} = 358 nm); b) CHX: cyclohexane (λ_{max} = 352 nm); c) ACN: acetonitrile (λ_{max} = 357 nm). Concentration of avobenzone ~2 x 10^{-5} mol dm^{-3}.

Assessing stabilisation effects in binary mixtures in ethanol

Binary mixtures of avobenzone and octocrylene (Merck) were prepared in ethanol and acetonitrile to observe any synergistic effects between the compounds. Octocrylene is often found in formulations with avobenzone. The absorbance change in the system was measured both at the absorbance peak and across the entire UVA range (area under the curve, AUC). In the binary mixtures in ethanol there was a reduced loss of activity at the UVA peak absorbance when avobenzone was combined with octocrylene (-3%). This is also the case across the entire UVA range. A stoichiometric ratio of 1:3 (avobenzone: octocrylene) was used as this is typical of a sunscreen product and results in an approximately equal absorbance at both peak maxima. From these data alone, it is difficult to distinguish the effect of spectral overlap and photon shielding from that of a true stabilising effect, especially when the overall loss of activity is low. When modelled with linear rates of photodegradation (**y = a + b*x)** the rate constants (**b**) are within error of each other and therefore these data do not appear to show any significant stabilisation at these concentrations in ethanol. Experimental data are recorded in Table 2 (main paper) and displayed in Figure S8.

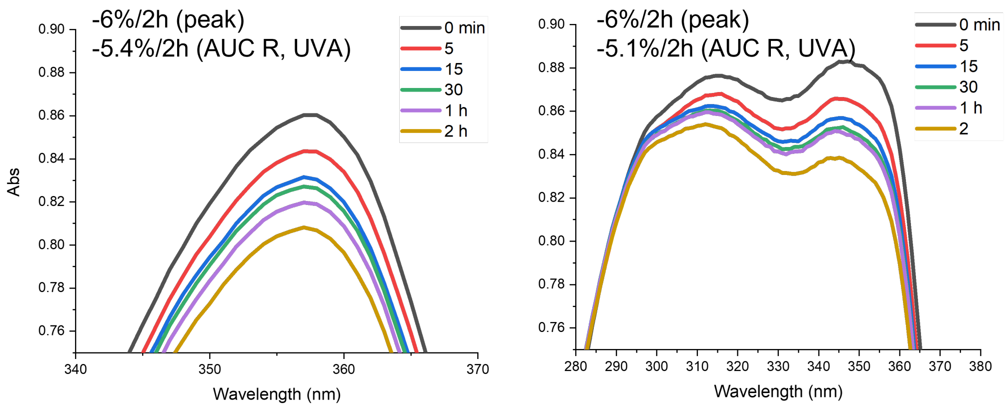

Wavelength (nm) Wavelength (nm)

Figure S8. A) Irradiation of avobenzone alone; B) Irradiation of an avobenzone and octocrylene mixture (1:3 molar ratio) in ethanol. Graphs are cropped. Inset are the loss of activity where 'max' indicates that the measurement is taken at the λ_{max} and UVA indicates that the change across the entire UVA range is considered. NB: Octocrylene was irradiated alone separately and showed no degradation.

Assessing stabilisation effects in binary mixtures in acetonitrile.

When assessed for stability under the same conditions used in the ethanol experiments, the results for acetonitrile (data in Table 4, main paper) show that the avobenzone UVA activity loss is significantly greater in the polar, non-protic solvent than in polar protic, with an increase to ~70% loss of activity during an hour of irradiation. In acetonitrile, the data show significant difference between avobenzone in the two-component mixture versus avobenzone alone. As a control, one cuvette containing only avobenzone was not irradiated and the loss of activity was considerably less (~7% vs. ~70%). This indicates that the major factor in driving the loss of the UVA activity is a light-driven process and there may be a minor effect of tautomerisation that occurs without irradiation and is due to the solvent environment alone. In any case in the two-component mixture the decrease in the UVA absorbance activity is reduced in line with the average degradation at peak absorbance but whether there is a direct stabilisation mechanism, or this is simply a

result of spectral overlap is unclear. Details of each experimental result are presented in Figure S9.

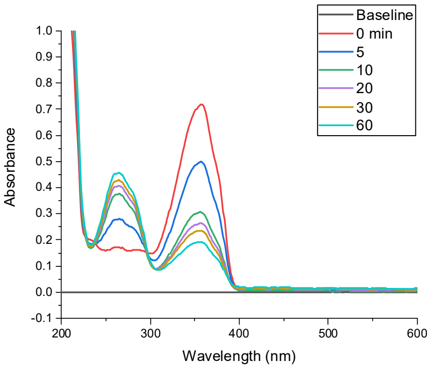

a)

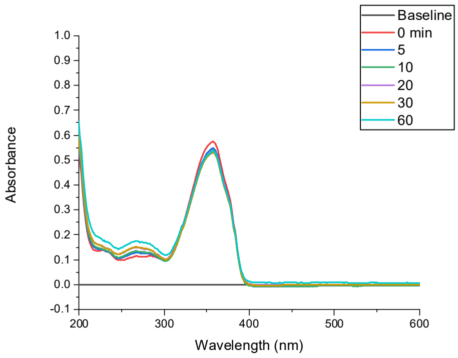

b)

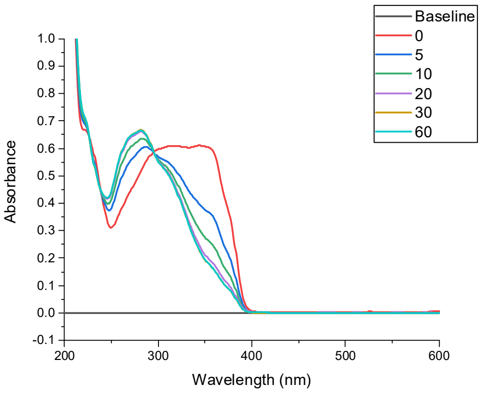

c)

Figure S9. Absorbance vs wavelength plots of binary mixtures in acetonitrile, room temperature. a) Irradiation of avobenzone alone; -73%/1h (max) -71% (UVA range/AUC), b) Avobenzone in ambient light (no artificial irradiation); -6.8%/1h (max), -4.5% (UVA range/AUC), c) Irradiation of an avobenzone and octocrylene mixture; -72%/1h (max) -58%/1h (UVA range/AUC AUC) .

.

Plots of the UV-vis activity of AVOCTO compounds 10a-10e in acetonitrile (main paper, Table 3) are shown in Figure S10:

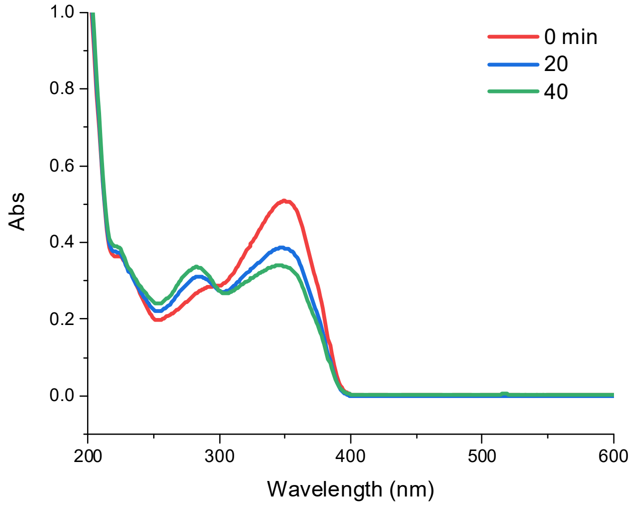

a) AVOCTO1 **10a** AUC R for in ACN 0.693, degradation per 40 minutes = 30.7%.

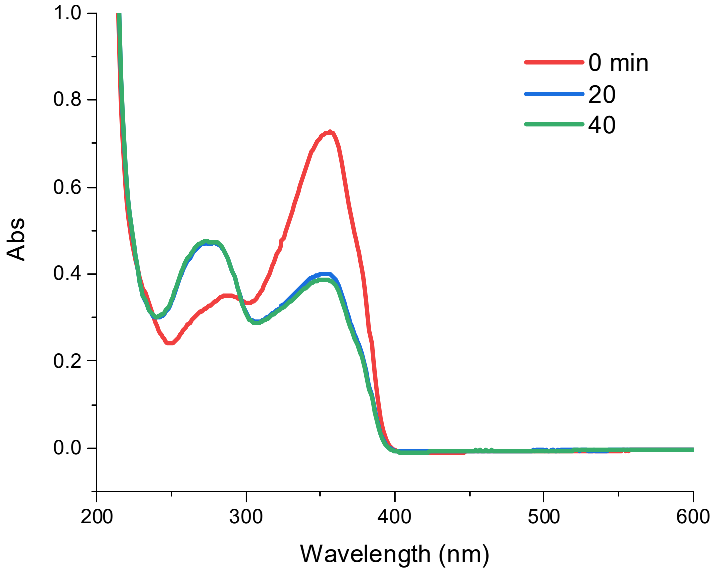

b) AVOCTO2 **10b** AUC R for in ACN 0.567, degradation per 40 minutes = 43.3%.

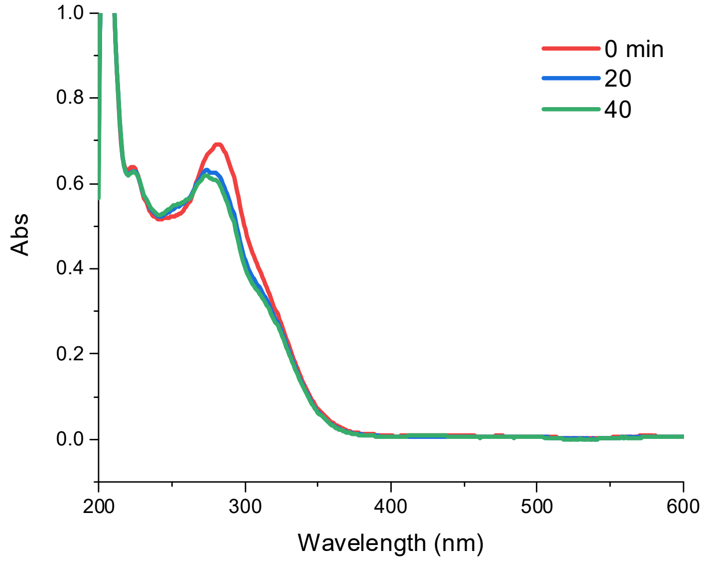

c) AVOCTO5 **10e** AUC R for in ACN 0.894, degradation per 40 minutes = 10.6%.

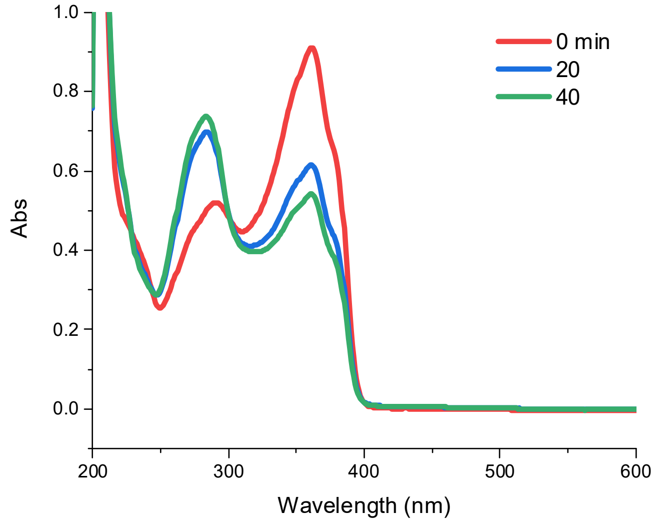

d) AVOCTO3 **10c** AUC R ACN 0.643, degradation per 40 minutes = 35.7%.

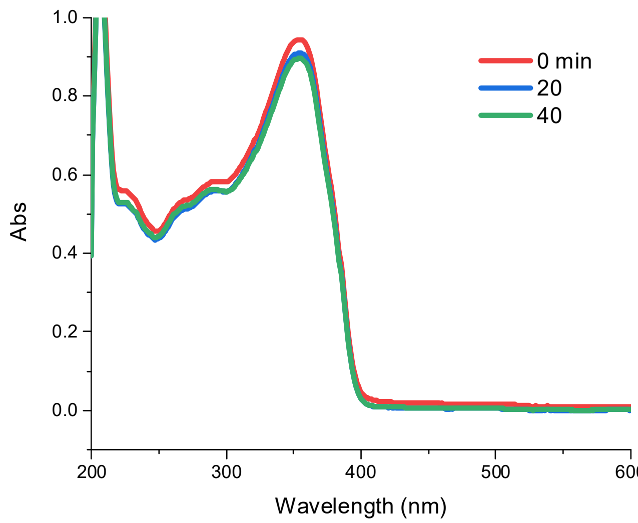

e) AVOCTO4 **10d** ACN AUC R 1.1058214, <1% degradation per 40 minutes.

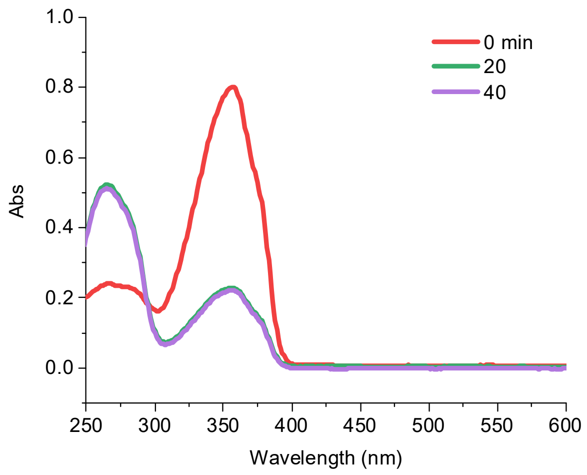

f) Avobenzone AUC R 0.278, -72.2% degradation by AUC R in 40 mins.

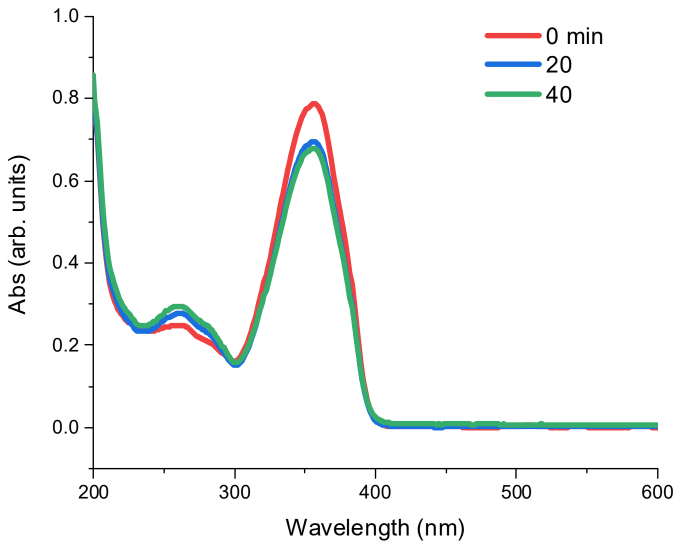

g) Chloroavobenzone AUC R 0.869, 13.1% degradation by AUC R.

Figure S10. Plots of the degradation of the UV-vis activity of AVOCTO compounds 10a-10e, avobenzone and chloroavobenzone in acetonitrile (main paper, Table 3).

5) Further ultrafast laser spectroscopy results.

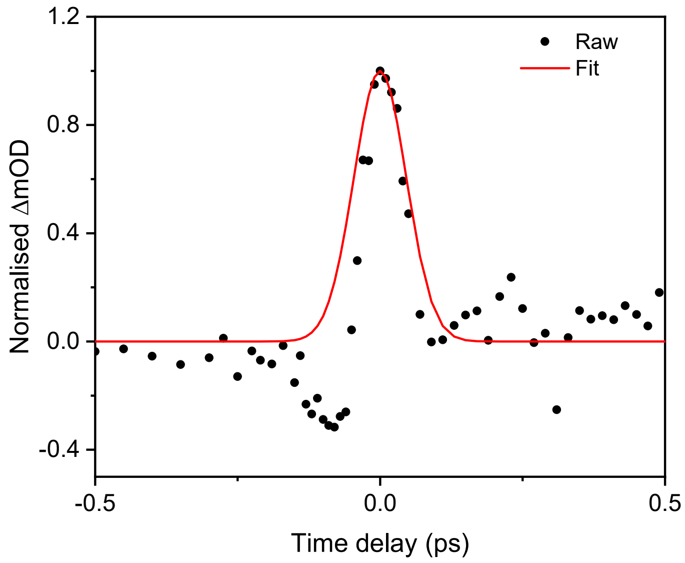

Figure S11. Solvent alone (acetonitrile) transient for instrument response function determination. Pump = 355 nm, Power = 1 mW and raw data were averaged between 400-405 nm (black circles). The pump-probe cross-correlation (F_{cc}) is overlaid (red line) and the returned FWHM of the F_{cc} is ~100 fs which corresponds to the FWHM of the instrument response function. The F_{CC} was calculated following a published analysis.[43]

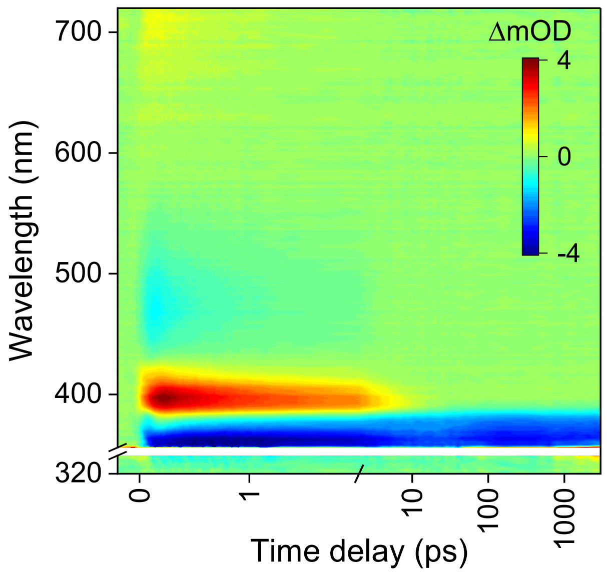

Figure S12. TAS for avobenzone presented as a false colour heat map in acetonitrile photoexcited at the pump wavelength 355 nm.

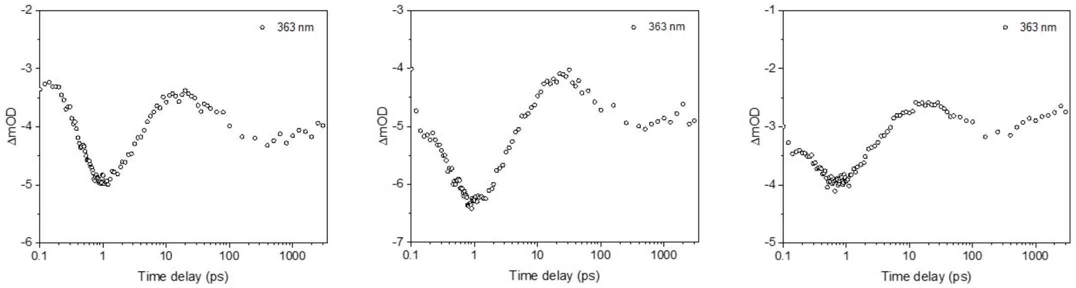

Figure S13. Transient at 363 nm for AVOCTO1 **10a** (left), AVOCTO**4 10d** (middle) and avobenzone (right) in acetonitrile after photoexcitation at 355 nm (350 nm for AVOCTO1 **10a**). An approximate ground state bleach recovery for AVOCTO1 **10a** and AVOCTO**4 10d** was ~20% and for avobenzone it was ~30%.

NMR study of AVOCTO4 10d photostability during TEAS analysis.

A complementary study that was carried out to confirm the stability of AVOCTO4 **10d** involved taking an NMR spectrum before and then immediately after TEAS analysis, in deuterated acetonitrile. The two spectra produced are displayed below (Figure S14). There was no significant change in the ratio of the peaks that correspond to the relative amount of the enol and keto forms (marked with dashed blue lines on the spectra). This study suggests that the same molecular entity is present before and after irradiation; but does not preclude that other molecules are formed that were not detected. Combined with the ultrafast studies this seems to confirm that the processes that produce a loss of activity in the UV are ultrafast and then, at longer times, the parent molecule is recovered.

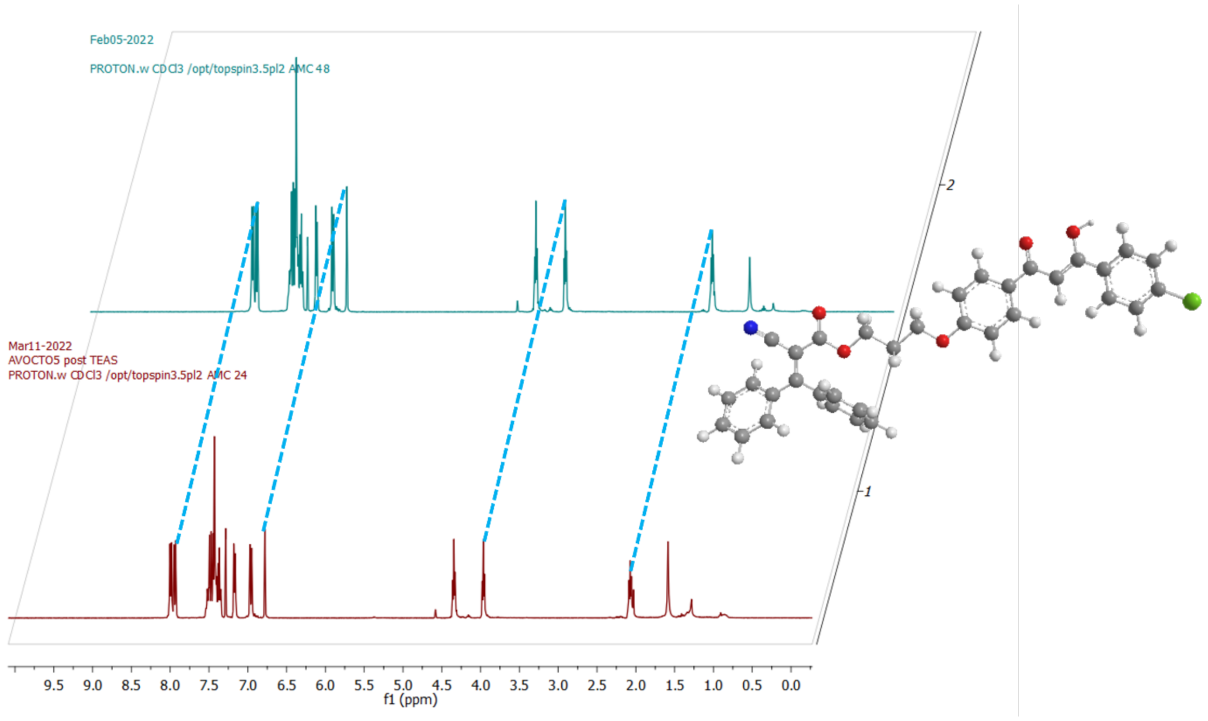

Figure S14. NMR spectra of AVOCTO4 **10d** before and after 60 minutes irradiation (same conditions as before in the longer pump wavelength scheme) in acetonitrile.

6) Computational AVOCTO calculations.

All calculations were performed using the NWChem software package.[44] Due to the large number of atoms and single bonds in the full avobenzone-octocrylene molecules, it was not possible to achieve convergence at the lower level of theory, hence only the truncated forms were studied. Density functional theory (DFT) geometry optimisations for the chelated enol and diketo structures of the avobenzone section of each molecule were studied only. These geometry optimisations were carried out to determine the most stable, lowest energy conformations in the ground state. These calculations were conducted in implicitly modelled acetonitrile, using the conductor-like screening model (COSMO, with SMD) built into NWChem.[45,46] The relaxation of the initial enol and diketo structures of each truncated molecule was initially carried out using DFT at the PBE0/6-31g* level of theory. This initial structure was then further optimized by increasing the basis set to 6-311++g**, before arriving at the final structure, which was calculated at the PBE0/6-311++g** level of theory.

Once the six optimised structures were attained, time-dependent DFT (TD-DFT) was carried out to attain the vertical excitation energies of the singlet (S1-5) states of each species in acetonitrile using the same COSMO model, using TD-DFT at the PBE0/6-311++g** level of theory. The state characters were also calculated during these TD-DFT calculations and assigned manually.

Table S1. Predicted singlet excited state vertical excitation energies for AVOCTO1 **10a** in its diketo and enol form calculated at the TD-DFT/PBE0/6-311++g** level of theory in implicitly modelled acetonitrile.

AVOCTO1 **10a**	**S_n**	**E (eV)**	**E (nm)**	**State character**	**Total oscillator strength**
Diketo form	S_1	3.84	322	nπ*	0.0003
	S_2	4.06	306	nπ*	0.0001
	S_3	4.34	285	ππ*	0.0142
	S_4	4.60	269	ππ*	0.4963
	S_5	4.74	262	nπ*	0.0143
Enol form	S_1	3.62	342	ππ*	0.8873
	S_2	3.97	312	nπ*	0.0002
	S_3	4.46	278	ππ*	0.0229
	S_4	4.50	275	ππ*	0.0227
	S_5	4.63	267	ππ*	0.0633

Table S2. Predicted singlet excited state vertical excitation energies for AVOCTO4 **10d** in its diketo and enol form calculated at the TD-DFT/PBE0/6-311++g** level of theory in implicitly modelled acetonitrile.

AVOCTO4 **10d**	**S_n**	**E (eV)**	**E (nm)**	**State character**	**Total oscillator strength**
Diketo form	S_1	3.83	324	ππ*	0.0004
	S_2	4.09	303	ππ*	(3.0802E-5)
	S_3	4.26	291	ππ*	0.0028
	S_4	4.60	270	ππ*	0.5655
	S_5	4.67	266	nπ*	0.0033
Enol form	S_1	3.57	347	ππ*	0.9422
	S_2	3.97	312	nπ*	0.0004
	S_3	4.40	281	ππ*	0.0732
	S_4	4.56	272	ππ*	0.0804
	S_5	4.62	268	ππ*	0.0080

Table S3. Predicted singlet excited state vertical excitation energies for AVOCTO5 **10e** in its diketo and enol form calculated at the TD-DFT/PBE0/6-311++g** level of theory in implicitly modelled acetonitrile.

AVOCTO5 **10e**	**State**	**E (eV)**	**E (nm)**	**State character**	**Total oscillator strength**
Diketo form	S_1	3.89	324	nπ*	0.0013
	S_2	4.00	310	nπ*	0.0018
	S_3	4.46	278	ππ*	0.0022
	S_4	4.59	270	ππ*	0.5159
	S_5	4.75	261	ππ*	0.0176
Enol form	S_1	3.81	326	ππ*	0.6110
	S_2	4.07	305	ππ*	0.1109
	S_3	4.57	271	nπ*	0.0203
	S_4	4.70	263	ππ*	(1E-5)
	S_5	4.76	260	ππ*	0.0069

7) EADS of AVOCTO1 10a and AVOCTO4 10d.

The evolution-associated difference spectra are included here to supplement Figure 7 and Table 4 of the main paper. These traces are effectively fits to the experimental data and are summarised by the time constants in Table 4. NB: Second trace τ_2 has amplitude in the SE region (500 nm) while the fourth trace τ_4 is purely a GSB containing no contribution from either the ESA (400 nm) or SE regions.

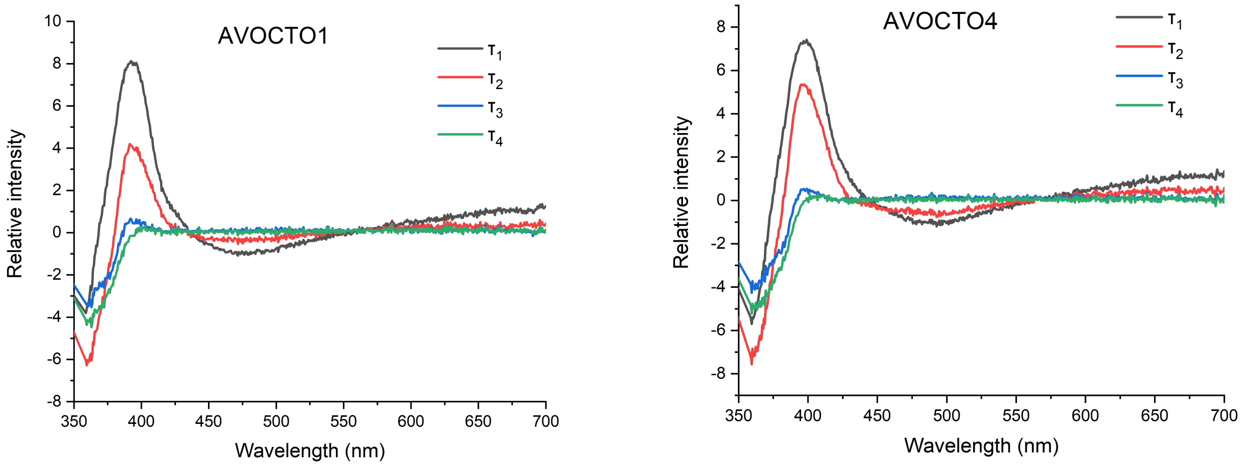

Figure S15. EADS of AVOCTO1 **10a** and AVOCTO4 **10d** presented as traces with associated time constants τ_1 to τ_4.

8) References.

(1) W. Schwack and T. Rudolph, Photochemistry of Dibenzoyl Methane UVA Filters Part 1. *J. Photochem. Photobiol. B: Biol.* 1995, **28**, 229–234.

(2) N. M. Roscher, M. K. O. Lindemann, S. Bin Kong, C. G. Cho and P. Jiang, Photodecomposition of Several Compounds Commonly Used as Sunscreen Agents. *J. Photochem. Photobiol. A: Chem.* 1994, **80**, 417–421.

(3) R. M. Sayre, J. C. Dowdy, A. J. Gerwig, W. J. Shields and R. V. Lloyd, Unexpected Photolysis of the Sunscreen Octinoxate in the Presence of the Sunscreen Avobenzone. *Photochem. Photobiol.* 2007, **81**, 452–456.

(4) M. Yamaji and M. Kida, M. Photothermal Tautomerization of a UV Sunscreen (4- *tert* -Butyl-4′-Methoxydibenzoylmethane) in Acetonitrile Studied by Steady-State and Laser Flash Photolysis. *J. Phys. Chem. A* 2013, **117**, 1946–1951.

(5) M. Dubois, P. Gilard, P. Tiercet, A. Deflandre and M. A. Lefebvre, Photoisomerisation of the Sunscreen Filter Parsol© 1789. *J. Chim. Phys*. 1998, **95**, 388–394.

(6) A. Cantrell and D. J. McGarvey, Photochemical Studies of 4-*tert*-Butyl-4′-Methoxydibenzoylmethane (BM-DBM). *J. Photochem. Photobiol. B: Biol.* 2001, **64**, 117–122.

(7) G. St. Nikolov and P. Markov, Photochemical Hydrogen Abstraction as a Radiationless Transition in the Photoketonization of β-Dicarbonyl Compounds*. J. Photochem*. 1981, **16**, 93–104.

(8) K. M. Hanson, M. Cutuli, T. Rivas, M. Antuna, J. Saoub, N. T. Tierce and C. J. Bardeen, Effects of Solvent and Micellar Encapsulation on the Photostability of Avobenzone. *Photochem. Photobiol. Sci.* 2020, **19**, 390–398.

(9) M. E. Burnett and S. Q. Wang, Current Sunscreen Controversies: A Critical Review: Sunscreen Controversies. *Photoderm. Photoimmunol. Photomed.* 2011, **27**, 58–67.

(10) S. Scalia, M. Mezzena and D. Ramaccini, Encapsulation of the UV Filters Ethylhexyl Methoxycinnamate and Butyl Methoxydibenzoylmethane in Lipid Microparticles: Effect on in Vivo Human Skin Permeation. Skin *Pharmacol. Physiol.* 2011, **24**, 182–189.

(11) G. Niculae, I. Lacatusu, A. Bors and R. Stan, Photostability Enhancement by Encapsulation of α-Tocopherol into Lipid-Based Nanoparticles Loaded with a UV Filter. *Comptes Rendus Chimie* 2014, **17**, 1028–1033.

(12) P. C. V. Govindu, B. Hosamani, S. Moi, D. Venkatachalam, S. Asha, V. N. John, V. Sandeep and K. H. Gowd, Glutathione as a Photo-Stabilizer of Avobenzone: An Evaluation under Glass-Filtered Sunlight Using UV-Spectroscopy. *Photochem. Photobiol. Sci*. 2019, **18**, 198–207.

13) T. Armeni, E. Damiani, M. Battino, L. Greci and G. Principato, Lack of in Vitro Protection by a Common Sunscreen Ingredient on UVA-Induced Cytotoxicity in Keratinocytes. *Toxicology* 2004, **203**, 165–178.

(14) E. Damiani, W. Baschong and L. Greci, UV-Filter Combinations under UV-A Exposure: Concomitant Quantification of over-All Spectral Stability and Molecular Integrity. *J. Photochem. Photobiol. B.* 2007, **87**, 95–104.

(15) E. Damiani, L. Greci, R. Parsons and J. Knowland, Nitroxide Radicals Protect DNA from Damage When Illuminated in Vitro in the Presence of Dibenzoylmethane and a Common Sunscreen Ingredient. *Free Radic. Biol. Med.* 1999, **26**, 809–816.

(16) D. Dondi, A. Albini and N. Serpone, Interactions between Different Solar UVB/UVA Filters Contained in Commercial Suncreams and Consequent Loss of UV Protection. *Photochem. Photobiol. Sci.* 2006, **5**, 835.

(17) G. Kornis and P. D. Mayo, Photochemical syntheses: 9. The conversion of dibenzoylmethane to tribenzoylethane. *Can. J. Chem*. 1964, **42**, 2822–2827.

(18) L. A. Baker, M. D. Horbury and V. G. Stavros, Ultrafast Photoprotective Properties of the Sunscreening Agent Octocrylene. *Opt. Express* 2016, **24**, 10700.

(19) S. Forestier, Rationale for Sunscreen Development. *J. Am. Acad. Dermatol.* 2008, **58**, 133-138.

(20) C. A. Bonda and D. Lott, Sunscreen Photostability. In Principles and Practice of Photoprotection; S. Q. Wang and H. W. Lim, Eds.; Springer International Publishing: Cham, 2016, 247–273.

(21) K. Sandros, Transfer of Triplet State Energy in Fluid Solutions. III. Reversible Energy Transfer. *Acta Chem. Scand*. 1964, **18**, 2355–2374.

(22) D. F. Evans, 257. Perturbation of Singlet–Triplet Transitions of Aromatic Molecules by Oxygen under Pressure. *J. Chem. Soc*. 1957, 1351–1357.

(23) H. Gonzenbach, T. J. Hill and T. G. Truscott, The Triplet Energy Levels of UVA and UVB Sunscreens. *J. Photochem. Photobiol. B: Biol*. 1992, **16**, 377–379.

(24) Kikuchi, A.; Hata, Y.; Kumasaka, R.; Nanbu, Y.; Yagi, M. Photoexcited Singlet and Triplet States of a UV Absorber Ethylhexyl Methoxycrylene. *Photochem. Photobiol.* 2013, **89**, 523–528.

(25) A. Kikuchi, N. Oguchi and M. Yagi, Optical and Electron Paramagnetic Resonance Studies of the Excited States of 4- *tert*-Butyl-4′-Methoxydibenzoylmethane and 4- *tert* -Butyl-4′-Methoxydibenzoylpropane. *J. Phys. Chem. A.* 2009, **113**, 13492–13497.

(26) M. A. El-Sayed, Double Resonance and the Properties of the Lowest Excited Triplet State of Organic Molecules. *An. Rev. Phys. Chem.* 1975, **26**, 235–258.

(27) B. Wardle, Principles and Applications of Photochemistry; Wiley, 2009.

(28) B. Herzog, J. Giesinger and V. Settels, Insights into the Stabilization of Photolabile UV-Absorbers in Sunscreens. *Photochem. Photobiol. Sci.* 2020, **19**, 1636–1649.

(29) J. J. Sacco, J. Botten, F. Macbeth, A. Bagust and P. Clark, The Average Body Surface Area of Adult Cancer Patients in the UK: A Multicentre Retrospective Study. *PLoS ONE* 2010, **5**, e8933.

(30) H. Hayashi and S. Nagakura, The Lowest Nπ* and Ππ* Triplet Levels of Benzaldehydes and Their Correlation with the Zero-Field Splittings. *Mol. Physics* 1974, **27**, 969–979.

(31) J. L Besombes, G. Cheminat, G. Mousset and C. Mousty, Synthesis of Electroreducible Amphiphilic Molecules - Electrochemical Study of Omega-(4-acetylphenoxy)alkyl Sulfates in an Aprotic Medium. *Bull. Soc. Chem. Fr.* 1992, **129**, 513–522..

(32) H. Jin, W. Zhang, D. Wang, Z. Chu, Z. Shen, D. Zou, X. Fan, Q. Zhou, Dendron-Jacketed Electrophosphorescent Copolymers: Improved Efficiency and Tuneable Emission Colour by Partial Energy Transfer. *Macromolecules* 2011, **44**, 9556–9564.

(33) J. Li, H. Chen, D. Zhang-Negrerie, Y. Du and K. Zhao, Synthesis of Coumarins via PIDA/I_2-Mediated Oxidative Cyclization of Substituted Phenylacrylic Acids. *RSC Adv.* 2013, **3**, 4311-4320.

(34) K. Subramanian, S. L. Yedage and B. M. Bhanage, An Electrochemical Method for Carboxylic Ester Synthesis from *N* -Alkoxyamides. *J. Org. Chem.* 2017, **82**, 10025–10032.

(35) V. Rysak, R. Dixit, X. Trivelli, N. Merle, F. Agbossou-Niedercorn, K. Vanka and C. Michon, Catalytic reductive deoxygenation of esters to ethers driven by hydrosilane activation through non-covalent interactions with a fluorinated borate salt. *Catal. Sci. Technol*., 2020, **10**, 4586-4592.

(36) Z. He, X. Qi, S. Li, Y. Zhao, G. Gao, Y. Lan, Y. Wu, J. Lan and J. You, Transition-Metal-Free Formal Decarboxylative Coupling of α-Oxocarboxylates with α-Bromoketones under Neutral Conditions: A Simple Access to 1,3-Diketones. *Angew. Chem. Int. Ed.* 2015, **54**, 855–859.

(37) C. Paris, V. Lhiaubet-Vallet, O. Jiménez, C. Trullas and A. Miranda, A Blocked Diketo Form of Avobenzone: Photostability, Photosensitizing Properties and Triplet Quenching by a Triazine-Derived UVB-Filter. *Photochem. Photobiol.* 2009, **85**, 178–184.

(38) F. Allais, S. Martinet and P.-H. Ducrot, Straightforward Total Synthesis of 2-O-Feruloyl-l-Malate, 2-O-Sinapoyl-l-Malate and 2-O-5-Hydroxyferuloyl-l-Malate. *Synthesis* 2009, 3571–3578.

(39) (a) T. Wang, G. Chen, Y. Lu, Q. Chen, Y. Huo and X. Li, Intermolecular Multiple Dehydrogenative Cross-Couplings of Ketones with Boronic Acids and Amines via Copper Catalysis. *Adv. Synth. Catal.* 2019, **361**, 3886–3892. (b) J.-C. Gramain, H.-P. Husson and Y. Troin, Synthese Stereospecifique Par Voie Photochimique de Trans Hexahydro-1,2,3,4,4a,9a Carbazolones-4 Substituees En 4a1. *Tetrahedron Lett.* 1985, **26**, 2323–2326.

(40) J. M. Woolley, M. Staniforth, M. D. Horbury, G. W. Richings, M. Wills and V. G. Stavros, Unravelling the Photoprotection Properties of Mycosporine Amino Acid Motifs. *J. Phys. Chem. Lett.*, 2018, **9**, 3043-3048.

(41) a) M. P. Grubb, A. J. Orr-Ewing and M. N. R. Ashfold, KOALA: A Program for the Processing and Decomposition of Transient Spectra. *Rev. Sci. Instrum*. 2014, **85**, 064104. b) J. J. Snellenburg, S. Laptenok, R. Seger, K. M. Mullen and I. H. M. Van Stokkum, Glotaran: A Java-Based Graphical User Interface for the R Package TIMP. *J. Stat. Softw*., 2012, **49**, 1-22.

(42) E. L. Holt, N. d. N. Rodrigues, J. Cebrián and V. G. Stavros, Determining the Photostability of Avobenzone in Sunscreen Formulation Models Using Ultrafast Spectroscopy. *Phys. Chem. Chem. Phys.* 2021, **23**, 24439–24448.

(43) S. A. Kovalenko, A. L. Dobryakov, J. Ruthmann, and N. P. Ernsting, Femtosecond spectroscopy of condensed phases with chirped supercontinuum probing. *Phys. Rev. A*, 1999, **59**, 2369.

(44) M. Valiev, E. J. Bylaska, N. Govind, K. Kowalski, T. P. Straatsma, H. J. J. Van Dam, D. Wang, J. Nieplocha, E. Apra, T. L. Windus and W. A. de Jong, NWChem: A Comprehensive and Scalable Open-Source Solution for Large Scale Molecular Simulations. *Computer Physics Communications* 2010, **181**, 1477–1489.

(45) A. Klamt and G. Schüürmann, COSMO: a new approach to dielectric screening in solvents with explicit expressions for the screening energy and its gradient. *J. Chem. Soc. Perkin Trans. 2*, 1993, 799-805.

(46) A. V. Marenich, C. J. Cramer and D. G. Truhlar, Universal Solvation Model Based on Solute Electron Density and on a Continuum Model of the Solvent Defined by the Bulk Dielectric Constant and Atomic Surface Tensions. *J. Phys. Chem B*, 2009, **113**, 6378-6396.
